# Supplementary material for: Implementation and staff perceptions of a quality assurance system in a Finnish private hospital during the COVID-19 Pandemic: A qualitative study
Source: PLoS One. 2025 Dec 4;20(12):e0327271. doi: 10.1371/journal.pone.0327271 (PMC12677441; doi:10.1371/journal.pone.0327271)
Supplement: S2 Dataset — (PDF) [file pone.0327271.s002.pdf]

Name of recording: Haastattelu1.mp3

Length of recording: 00:50:16

Information:

[?] = Word was not understood completely but meaning is almost correct. Recording point is written to the text e.g. [word? 00:15:44]

[??] = Word could not be understood and therefore could not be written. Recording point is written to the text e.g. [?? 00:15:44]

[text] = Sounds or not transcribed parts are written with square brackets e.g. [laughs] or [interview paused due to a phone call]

[recording starts]

Haastattelija [00:00:01]: ...mä laitan tän nauhoituksen päälle, niin alotetaanko siitä, että miten te ootte osallistunu tähän laatujärjestelmän käyttöönottoon ja mitä te tiedätte tässä vaiheessa siitä?

Puhuja 1 [00:00:11]: Mä olin alussa yhessä ryhmässä, mut sit se ryhmä tossa tavallaan hajoskin, ja sitten mä jäin pois, koska mä oon niin... mä oon vaan puoltoista päivää viikossa, oon todella.

Haastattelija [00:00:23]: Joo.

Puhuja 1 [00:00:24]: Ja et aika vähän tiiän.

Haastattelija [00:00:28]: No niin. Mites muut?

Puhuja 2 [00:00:30]: Sama juttu, ei mekään oikeastaan. Vaikka me olemme ihan kokopäiväsiä ja ollaan jatkuvasti täällä. Ei kauheasti olla perillä tästä asiasta.

Haastattelija [00:00:39]: Okei. Ootteko te kuullu sen JCI...

Puhuja 2 [00:00:43]: Joo, no se nimi on kuultu kyllä, mutta ei paljon mitään muuta siitä sen kummempaa.

Puhuja 3 [00:00:47]: Mä oon joutunu osallistumaan, en tosin niissä ryhmissä, mutta etsimällä tietoa, mitä kaikkea löytyy, liittyen tietysti omiin näihin alueisiin. Sitten joudun, tiedän sen, et joudun rupeen rakentaa yhtä osiota, joka sitten toivon mukaan pystytään käyttään sitte siinä JCI:ssä hyväksi.

Haastattelija [00:01:16]: Joo, mahtavaa.

Puhuja 3 [00:01:17]: Silleen niinku taustatyötä tekemään.

Haastattelija [00:01:20]: Hei, hyvä kun pääsit. Mä oon siis (…) ja mä oon tutkijana tässä hankkeessa. Sul on varmaan mulle semmonen lomake, et saatasko me nauhottaa tää.

Puhuja 4 [00:01:27]: Joo. Kyllä.

Haastattelija [00:01:28]: Hyvä. Ja tätä käytetään kehittämistarkoituksiin, ja just selitin muille, että kehittämis- ja tutkimustarkooituksiin näitä vastauksia, ja tämän saa keskeyttää milloin tahansa, ja tää on täysin vapaaehtoista.

Puhuja 4 [00:01:38]: Kyllä.

Haastattelija [00:01:40]: Mut me alotettiin jo hiljalleen, et mitä te tiedätte tästä JCI-järjestelmästä.

Puhuja 1 [00:01:42]: Sen verran mä tiedän sanoo, että mulla on kyllä täällä kokoaikainen työsuhde olemassa, mut nyt oon tässä väliaikaisesti ainakin. Saa nähdä mitä sit tulevaisuudessa. Mut nyt teen vähemmän.

Puhuja 3 [00:01:56]: Ja varmaan rupee sit kun niit saadaan tehtyä, kaikki ne kriteerit, niin varmasti sitten helpottaa tavallaan omaa työtä, että tulee yhtenäiset tavallaan ohjeistukset kaikkeen. Et kyl mä uskon, et se on varmaan ihan tarpeellinen.

Haastattelija [00:02:16]: Miten […], niin mites ooksä osallistunu näihin, laatujärjestelmän käyttöönottoon tai näihin orientaatioihi?

Puhuja 4 [00:02:24]: No, mä luulen et siit on niin kauan aikaa, kun oon ollu niis mukana, että... mähän oon nyt kato aika vanha jo, että... joo. Täs helmikuus 70.

Haastattelija [00:02:36]: Niin. Mutta teil ei ollu alkuvuodesta, ei ollu semmosii, tai oli varmaan semmosii...

Puhuja 3 [00:02:41]: On ollu. En ollu talossa kyllä...

Puhuja 4 [00:02:43]: Joo, kyllä me ollaan niitä, oikeestaan kaikki tehty.

[Puhujat ja Haastattelija myöntelevät 00:02:46]

Puhuja 3 [00:02:47]: Kaikki ei oo varmaan niissä työryhmissä, ne on valittu henkilöt niihin, ni...

Haastattelija [00:02:53]: Mut ootteks te osallistunu niihin ty... Kuka teistä osallistu niihin työryhmiin tai niihin koulutuksiin, mitä oli sillon alkuvuodesta ennen korona-aikaa?

Puhuja 3 [00:03:01]: Kyl mä olin kuuntelemassa jonkun intron siitä. Mutta tota, työryhmissä en oo varsinaisesti työryhmän jäsen missään niissä, mut sitte joudun tekemään totta kai niihin liittyviä asioita.

Haastattelija [00:03:16]: Mm.

Puhuja 1 [00:03:17]: No mä olin varmaan kans kuuntelemassa jonkun infon, ja sit tosiaan olin semmosessa ryhmässä, mut sitte tää ryhmänvetäjä halus siirtyä pois siitä, niin sitten mäki siirryin pois. Jotenki se meni [silleen? 00:03:30].

Haastattelija [00:03:30]: Joo.

Puhuja 1 [00:03:31]: Ja, ja en tiedä nykysellä työajalla, että en varmaan pystyis kamalasti siihen osallistumaan, ja.

Haastattelija [00:03:38]: Joo. No onks teillä kuitenkin, kun te ootte kuulleet, puhun täst, niin onks teillä jonkunnäkösiä odotuksia? Ira jo alotit siitä, että voi olla, et helpottaa omaa työtä ja yhteiset ohjeistukset auttaa. Onks muut, mitä te muut ajattelette, että jos tulee tämmönen, tai kun tulee tämmönen laatujärjestelmä käyttöön, että miten se vaikuttaa teiän arjen työhön?

Puhuja 1 [00:04:03]: No mä aattelisin jotenkin niin, että tiettyihin, että mä oikeesti toivon, että tää on hyvä juttu ja kehittävä juttu, ja sellanen, että tulee niinku toimintaperi-, että ruodittas jotenki niin, et se tulis siihen käytäntöön, et ihan oikeesti näkyis sit siinä käytännössä ne muutokset, ja ne parannusehdotukset ja... ja ihan siinä asiakastyössäkin. Että miten toimitaan esimerkiks, mä nyt oon kuntoutuksen puolella töissä, miten toimitaan, kun joku pitäis kotiuttaa, mut oikeesti sitä ei voikaan kotiuttaa, kun sil on jotain, ja kuka tekee, mitä tekee.

Haastattelija [00:04:47]: Joo.

Puhuja 1 [00:04:48]: Et ihan niinku se, tää nyt oli yks esimerkki, mut sellanen ihan tarkasti, kuka tekee, mitä tekee, se prosessi jotenki. Et tavallaan kun ei voi kotiuttaa, jos on ihminen siinä tilanteessa, että sehän on meiän vastuulla kuitenkin. Tai lääkärihän sen aina viimeks päättää, että mitä tehään.

Puhuja 2 [00:05:09]: Niin, sitä mäkin toivon, että ne yhteiset ohjeistukset tulis selvemmiks, että...

Puhuja 3 [00:05:15]: Ja nythän ne kanssa tän myötä...

Puhuja 2 [00:05:17]: Infektiosiivousten ihan tarkat siivousohjeet, selkeät, selkeät ohjeet. Ettei siellä jokainen sovella omiaan. Ja aineet, millä aineella pestään mitäkin, mikäkin patteri siel on, mitäkin tehdään. Ihan se. Nyt on vähän...

Puhuja 3 [00:05:34]: Tätä myötä nyt on pakko päivittää kaikki ohjeistukset.

[Puhujat ja Haastattelija myöntelevät 00:05:39]

Puhuja 3 [00:05:41]: Tähän päivään ajan tasalle.

Haastattelija [00:05:48]: No oot-, aatteletteksen, et tämmösestä laatujärjestelmän käyttöönotosta vois olla jotain haittaa teiän työyhteisölle tai työn tekemiselle?

Puhuja 2 [00:05:56]: Ei tuu ainakaan mieleen mitään haittoja siitä.

Puhuja 3 [00:05:58]: Kuhan se saadaan tehtyä!

Puhuja 2 [00:05:59]: Niin varmaan sit käytäntö...

Puhuja 3 [00:06:01]: Ni sit käytäntö ehkä sit osottaa...

Puhuja ? [00:06:04]: Joo, et on työ.

Puhuja 1 [00:06:08]: Kyl se varmaan sitte on helpompaa, sitte päivitellä niitä, kun on kerran käyty kaikki läpi ohjeistukset, ni.

Puhuja 2 [00:06:17]: Ja kyl ku on nähny sitä taulukkoo, tai jotenki mitä sinne pitää laittaa, ni ei se mikään yksinkertanen oo.

Haastattelija [00:06:22]: Niin et iso työ kuitenki?

Puhuja 2 [00:06:23]: Nii, ja sisäistää sen, et kyl ne tarvii aikaa siihen, jotka sitä... on niissä työryhmissä.

Haastattelija [00:06:32]: Mm. Te puhuitte noista yhteisistä ohjeistuksista, niin aatteletteko, et mitä muita seurauksia sillä vois olla tän toiminnan laatuun tai teiän työn laatuun? Että se, että kaikki tekee samalla tavalla ja ohjeistusten mukaisesti, mutta...?

Puhuja 1 [00:06:44]: Kyl mä uskon, et se parantaisi.

Puhuja 2 [00:06:50]: Kyllä.

Puhuja 3 [00:06:50]: Ehkä mä uskon liian paljon [naurua 00:06:51]. Mut kyl mä luotan jotenki siihen, että... kyl siinä on...

Puhuja 2 [00:06:57]: Niin, no kyl se selkeyttää ainakin.

Puhuja 1 [00:06:59]: Selkeyttä, ja varmaan tulee semmost työilmapiiriinki vaikutusta, koska kaikki tietää, että näin toimitaan ja näin tehään. Sit ei tarvi niinku, että no höh, taas se jätti sen tekemättä, miksei tätä tai...

Puhuja 2 [00:07:13]: Ja sit jos tulee joku uus ihminen, tai opiskelija tai joku, ni sit sä voit opettaa, antaa sille ohjeet käteen, et näin meillä tehään, tällä tavalla ja näin, et sä voit johonki vähän niinku nojaa sit, että. Kaikki sitte, kaikki ketkä on työvuorossa, meitäki on kolme, ni jokainen ku on työvuorossa sen uuden ihmisen kanssa, ni sit jokainen samalla tavalla, ni sit se uus ihminen ei oo, että toi teki näin ja nyt toi tekeekin noin, ja sit kolmaski tekee vielä eri tavalla. Sit on ihan niinku, et miten se pitääkään tehä.

Puhuja ? [00:07:42]: Kyllä.

Puhuja 2 [00:07:45]: Vaikka on siivouksest kyse, mut se on tosi tärkeetä.

[Puhujat ja Haastattelija myöntelevät 00:07:47]

Puhuja 1 [00:07:48]: On, on, se on erittäin tärkeetä.

Puhuja 2 [00:07:49]: Se on tosi tärkeetä.

Haastattelija [00:07:52]: Entäs lääkärin työssä?

Puhuja 4 [00:07:55]: No se on tietysti tämmöstä yksinäistä työtä, että no. Tietysti hyvä käytös ja sellanen, et saa potilaan luottamuksen heräämään, ja sit tietysti vastuu näistä, taloudesta, et se on kans semmonen mun mielest hyvin tärkee pointti, että jokainen potilas täytyy saada tänne ja hoitaa hyvin, ja sitte että se hänen, mitä hän kertoo ympärilleen, ni se vaikuttaa siihen, et kuinka paljon meille tulee tänne potilaita. Se on tietysti. Ja miten pienestä nää on loppujen lopuks kiinni nää, et voi jäädä hyvä tai huono muisto tästä tapaamisesta tai sairaalassa olosta. Ja ehkä just sit se, että meidän, kun me ollaan palveluammatissa, ni me pyritään sit kuitenkin aina, meidän täytys tuntea se henkilö hyvin ennen kun jotain invasiivista, tai jotain muutakin ihan, tehdään. Ni mitä paremmin me tunnetaan potilasta, ni se on jo usein puoli voittoa, että se koskee tietysti kaikkia henkilöitä, jotka kuntoutuksessa tai sairaalassa tai poliklinikalla tai missä ammatissa tahansa toimivat.

Puhuja ? [00:09:18]: Kyllä.

Haastattelija [00:09:21]: Aatteleks, et tällä laatujärjestelmän käyttöönotolla vois olla jotain vaikutuksia työturvallisuuteen, tai...?

Puhuja 3 [00:09:28]: Kyllä mä uskoisin. Kun on yhteneväiset ohjeistukset kaikilla, ja kaikki huomioi ne asiat, niin kyl mä uskon.

Haastattelija [00:09:43]: No entäs sitten työmotivaatio?

Puhuja ? [00:09:46]: Sekin.

Puhuja 3 [00:09:47]: Varmaan se vaikuttaa siihenkin jollain lailla, mutta... Mä ehkä enempi uskon, et se motivaatio tulee itsestä.

Haastattelija [00:09:57]: [naurahtaa] Nii.

Puhuja 3 [00:09:59]: Ja samalla semmoinen työhyvinvointi niinku...

Puhuja 2 [00:10:01]: Niin, totta kai. Itestähän se kumpuaa.

Puhuja 1 [00:10:04]: Itestä se kumpuaa, mut sitte ne puitteet. Kylhän ne vaikuttaa siihen.

Haastattelija [00:10:14]: No mitä te, (…] puhuit tosta tän talon taloudesta, et jokainen potilas pitää saada tänne, niin miten te muuten aattelette, et onks tämmösellä laatujärjestelmän käyttöönotolla muita vaikutuksia sairaalan tulevaisuuteen?

Puhuja 3 [00:10:27]: Varmasti se antaa positiivisen kuvan ulkopuolelle, et meil on käytössä laatukriteerit, laadunvalvonta, ni kyllähän se mun mielestä kertoo, että me välitetään siitä, mitä me tehdään. Ja kyllähän usein kuulee sitä, et potilaat on hyvin tarkkaan tutkinu meidän talon ja henkilöt ja näin ennen ku ne tulee tänne, että...

Puhuja 1 [00:11:03]: Kyllähän meillä kuntoutuksessa nytte joka viikko, kun meil on eri kuntoutujat, ni tulee, otetaan palaute. Siis kirjallinen palaute, ja se on must erittäin hyvä. Tai tietysti on parasta, jos sen huononkin palautteen - pääosin se on erittäin hyvää palautetta - jos ne epäkohdatkin pystyy heti tarttumaan, jo siinä vaiheessa, ku se ihminen on täällä, että mihin voi vaikuttaa.

Puhuja 3 [00:11:27]: Sairaalan puolel sama juttu. Jokasel potilaalla on palautuslomake siellä yöpöydän laatikossa.

Haastattelija [00:11:33]: Miten paljon ne palauttaa niitä lomakkeita, onks ne niinku...?

Puhuja 1 [00:11:38]: No melko hyvin kyllä.

Haastattelija [00:11:39]: Joo.

Puhuja 1 [00:11:40]: Et niinku... ja kyl se on mun mielest, mulle se ainakin tuo sitä myös, sitä omaa motivaatioo siihen työhön, ja sitä sellasta... Emmä tiedä. Aluks mä koin vuosia, vuosia

sitten sen hankalana, et apua, aina palaute, mut se on erittäin hyvä. Että mehän ollaan täällä potilaita, asiakkaita, kuntoutujia varten, että...

Puhuja 3 [00:12:02]: Niin sit ei voi ikinä tietää, jos tekee ite jotain potilaan mielestä väärin, koska jos ei kukaan kerro sitä... Se on ihan hyvä, et silt potilaalt tulee joku palaute, että...

Haastattelija [00:12:12]: Miten te käsittelette niitä palautteita sitte? Käsitteletteks te jotenki yhteisesti, vai tuleeks se aina sille työntekijälle?

Puhuja 3 [00:12:18]: Yhteisesti kyllä meillä.

Puhuja 2 [00:12:19]: Yhteisesti, joo.

Puhuja 1 [00:12:22]: Ja sitten se, että ne on näkyvillä, henkilökunta voi mennä kattomaan aina viikon jälkeen.

Haastattelija [00:12:26]: Niin justiinsa, tulee kaikille.

Puhuja 1 [00:12:29]: Ja sitten jossain ehkä kokouksessa sitte käyään pidemmän ajan, aikavälin. Ja tietysti esimies sitte, jos on negatiivista, ni sitten niinku et mitä vois tehä, ja onks siihen jotain, että onko... tai onks se jostain johtuvaa. Mut kyl se aina käydään läpi.

Puhuja 4 [00:12:51]: Siis onks positiivinen palautekin? Esim mä oon tos Eirassa myöskin, ni siellä mun mielestä työntekijöitä motivoi hyvin se, että myöskin johto antaa sen positiivisen palautteen, et taas tuli sulle kiitoksia, ja se on hyvin tärkeää.

Puhuja 1 [00:13:08]: Kyllä, se on erittäin tärkee, kyllä joo. Kyllä se positiivinenkin.

[Puhujat myöntelevät 00:13:11]

Puhuja 4 [00:13:14]: Ja sit tietysti tän laatujärjestelmän seurannan kannalta on hyvä, että käydään läpi sitten, et mitä me ollaan päätetty aikanaan, ja onks se toteutunu. Jos on jotain

tapahtunu sellasta, mikä ei... mikä ei ehkä... ni se auttaa selvittään, et miten me parannetaan tässä.

[Puhujat myöntelevät 00:13:32]

Haastattelija [00:13:37]: Aatteletteks te, et se palautejärjestelmä on osa tätä laatujärjestelmää, tai aatteletteks te jotenki, et se on erikseen? Erillisenä tästä?

Puhuja 1 [00:13:47]: Nii, kyllähän se kuuluu... Mutta se on tavallaan se, mitä on nyt ollu käytössä.

Haastattelija [00:13:52]: Mitä on ollu jo aikasemmin käytössä, kyllä. No miten tää laatujärjestelmä teidän mielestä liittyy leikkauksiin tai potilasturvallisuuteen tai tämmösiin, että aatteletteko, että sillä on tällä laatujärjestelmän käyttöönotolla vaikutuksia näihin leikkauksiin liittyviin tekijöihin? Leikkaussalitoimintaan?

Puhuja 3 [00:14:10]: Kyllä mä uskon, et sil on tosi vahva, iso vaikutus siihen.

Haastattelija [00:14:14]: Ja minkälaisia vaikutuksia?

Puhuja 3 [00:14:16]: Siis ihan et potilasturvallisuus, et siinä varmaan tulee tämmösiä... onhan nytkin jo checkilistoja ja tällasia, mut se, että huomataan, että kaikki on käyty läpi mitä pitää huomioida. Kyllä mä uskon siihen, et se on. Ja jos kaikki tuntee sitten ne kriteerit, ja mitä on tehty ja mitä pitäisi tehdä, ni kyllähän se sillon pitäis toimia. Näin mä ajattelen. Et ilman muuta siihen turvallisuuteen liittyy.

Haastattelija [00:14:54]: Mm. No aatteletteko, että se leikkaussalitoiminta saattas nopeutua tai jotenkin tehostua tämmösen myötä, vai onks sillä sen kans mitään tekemistä?

Puhuja 3 [00:15:06]: En mä osaa sanoo. Voisin kuvitella, et varmaan vois auttaa jossain kohdissa prosessia.

Puhuja 4 [00:15:15]: Varmaan just näihin odotusaikoihin, että ne pitäis paikkansa paremmin, ettei tulis semmosia, että siellä iso joukko odottaa. Ku potilasta ei tuoda esimerkiks

osastolle, tai... en mä nyt tarkota tätä tasoa, taloa välttämättä, mut semmosii tilanteita, jossa se aika kuluu ja ei päästä siihen toivottuun tulokseen, ni se on varmaan aika pitkälle tästä riippuvaista. Ja tässä just, koska nää, niinku sanoit, nää potilaat on meiän, tai nää kuntoutujat on meiän vastuulla, että niiden ominaisuuksien hyvä tunteminen ja... tietysti myöskin sen niiden luonteen ja kaikki, jotka hoitohenkilökunta tekee, ni laittaa ne oikeisiin huoneisiin, et pärjää toistensa kanssa. Ja ikä- ja kieliasiat huomioiden, kaikki tämmöset.

Haastattelija [00:16:21]: Miten te ootte jakanu sitä, ku sanoit että on iso työ tehä niitä taulukoita ja kerätä niitä tietoja, niin ootteks te paljon jakanu siihen laatujärjestelmään liittyvää työntekoo, niinku tasasesti tai tälleen...

Puhuja 3 [00:16:32]: Mun kohdalla se tuli yhden tämmösen työryhmän jäsenen tai vetäjän myötä, et hän sitte seuraavaan instanssiin ilmotti, et kaivakaa kaikki mahdolliset tiedot tästä, jokainen omalta alueeltanne, ja nyt sitte seuraavaks tulee tässä lääkehoitosuunnitelmassa, kun se pitää nyt tehdä uusiks, niin siinä sitten, et se voidaan käyttää pohjana sitten laatukriteereissä, kun jokainen yksikkö käy ne läpi.

Haastattelija [00:17:07]: Joo.

Puhuja 3 [00:17:09]: Ni se on sit sitä pohjatyötä siihen. Et vaikka en varsinaisesti tosiaan kuulu niihin työryhmiin, ni... mut sitte omalta alueelta.

Haastattelija [00:17:21]: Onks se sun työhön vaikuttanu, tää nyt tällä erää tämä laatujärjestelmä?

Puhuja 4 [00:17:28]: No en mä oikeen osaa sanoo, että musta tuntuu että kun mä en oo kokoaikaisesti täällä, niin... ja sitten en ehkä seuraa nyt tätä, näitä infojakaan, kyllä tietysti ton sähköpostin kautta, mutta jos mä oisin täällä tosiaan viitenä päivänä viikossa, niin sillon se ehkä vähän eri lailla. Mut kyllä mä kuulen niistä sitte, et kyllä sillä lailla vaikuttaa.

Haastattelija [00:17:55]: Kyllä. Mites muut? Onks se teillä niinku... ei tiietä? Ei?

Puhuja 2 [00:18:02]: ...ainakaan oo vielä mitään semmosta. Et on niin vähän varma, et meil ei ookaan aikaa kaikki aika siihen työhön.

Puhuja 1 [00:18:14]: Mut varmaan se ois ihan hyvä sitten, et kun on just meitä, jotka ei niihin ryhmiin kuuluta... Sä oot saanu selkeitä tämmösiä tehtäviä, mutta se, että tavallaan ois joku semmonen, missä vois tuoda mitä huomaa, vaikkei kuulu siihen porukkaan, niin joku kanava, että vois...

Haastattelija [00:18:35]: Niin tarkotatko jotain palautelaatikkoa tai jotain...

Puhuja 1 [00:18:39]: No jotain vaikka sähköpostia, tai nimettömänä palautelaatikkoon, et voisko tämmöstä asiaa sitten ottaa huomioon siinä [?? 00:18:44].

Puhuja 2 [00:18:47]: Ihan hyvä idea.

Puhuja 3 [00:18:49]: Se vois olla hyvä se palautelaatikko.

Puhuja 1 [00:18:52]: Nii-i, vaikka.

Puhuja 3 [00:18:52]: Koska se, niin, asiathan tulee mieleen aina... pulpahtelee...

Puhuja 1 [00:18:55]: No niitä tulee, pulpahtelee, just kun on se tilanne siinä.

Puhuja 3 [00:18:59]: Just näin, joo.

Haastattelija [00:19:02]: Onks teillä muita ajatuksia, mitä teiän mielestä tässä laatujärjestelmän kehittämisessä pitäs nyt tässä vaiheessa, tässä alkuvaiheessa, ottaa huomioon? Että miten siitä saatais semmonen, et se palvelis teiän työtä parhaiten?

Puhuja 3 [00:19:15]: Varmaan siinä on just tää ryhmien, työryhmien tämmönen moniammatillisuus. Et siinä on eri ihmiset koottuna tiettyihin asioiden äärelle, ni et siel ei kukaan joudu sitte yksin sitä puoltamaan ja näin, et se saa varmaan heijastetta.

Puhuja 1 [00:19:41]: Nii, ja sitten kun me jokainen edustetaan omaa ammattiamme, me nähdään se jotenki. Ja sitten taas... mitä ei välttämättä, niinku sun työstäkään ei huomaa, eikä mun työstä, eikä [?? 00:19:55].

Puhuja 2 [00:19:58]: Paitsi sillon kun mun työ on tekemättä. Sen huomaa sillon. [Sit on heti? 00:20:01] et siivooja ei oo käyny täällä. [Huomaa? 00:20:03] et no niin. Sit ku on käyny, niin kaikki vaan...

Puhuja 1 [00:20:07]: Nii-i, kun on itestään selvää.

Puhuja 2 [00:20:09]: Joku tossa kävelee, kukahan se on? [naurua 00:20:10] Joo-o, se on.

Haastattelija [00:20:18]: No miten tää, että kun puhuitte paljon näistä kuntoutujista, ja täällä ollaan niitä potilaita varten tai asiakkaita varten, ni aattelettekste, et se jotenkin näkyy se laatujärjestelmä muutenki sitte sinne potilaalle asti? Tai pitääkö se teiän mielestä näkyä edes, vai onks se niinku teiän... et sä voit puhuu, joku saatto sanoa äsken, et on tärkeetä et voidaan sanoo, et meil on tämmönen laatujärjestelmä käytössä, mutta...

Puhuja 3 [00:20:45]: Mm, no kyllä se varmaan tulee jonnekin nettisivuille sitten, että meillä on tällaset, tavallaan informaationa niille, jotka lukee niitä nettisivuja, mut se, että ei se nyt ehkä just sit se yksittäinen siinä näe sitä. Mut kyllähän se, mä koen et se ohjaa varmasti jollain tasolla meiän työtä.

Puhuja 1 [00:21:09]: Mä mietin kans sitä samaa, et no toki nytki yritetään tehä mahollisimman laadukasta työtä, mutta et työntekijöiden kauttahan se varmaan, tai mä näkisin et se tulee. Miten me se sisäistetään, tai miten me sisäistetään yleensä se palvelu ja...

Puhuja 4 [00:21:27]: Kyl must tuntuu, et se asiakas tai potilas tai kuntoutujakin sen näkee. Mul oli kokemus, mä olin tos kesällä viis päivää julkisessa sairaalassa, ja olin ei-lääkärinä siellä, potilaana, kukaan ei tienny et mä oon lääkäri. Ja näin sen pienessä kuuden hengen huoneessa, näin koko ajan, et mitä ei olis pitäny... et siel koko aika tapahtu virheitä ja poikkeamia. Ja sitten mä olin siellä ehkä, leikkaussalissa, semmonen tunnin verran, ja sit mä taas näin, et siellä oli kaikki kunnossa. Todella hienosti viimisen päälle. Et siinä mä ainakin tunsin, et mä olen täysin... Esimerkkinä, et mä olin just tulos leikkaussalista, niin tuodaan kuumeinen, siis septinen mies, joka... viereen nukkumaan siihen keskellä yötä, ja joka sano ensimmäisenä, et on tääki aikaa kesälomaa viettää. Että ei ne taaskaan saa tätä bakteerii millään pois. Mä katoin, et mikä antibiootti siellä roi-, oli tippumassa, ja keräsin kamani. Mä tiesin, et siin on sairaalahygieniast merkittävä bakteeri, ja menin yökölle sanomaan, et mä

en mee tonne enää nukkumaan sit. Ja sit mä nukuin siellä aulassa. Et niinku näki, et vaik on (…) sairaala, niin kerta kaikkiaan siel ei toiminu oikeestaan siellä vuodeosastolla mikään.

Haastattelija [00:23:03]: Joo. Et sitten sanonu, että mikset menny nukkumaan, että...?

Puhuja 4 [00:23:07]: Mä sanoin sille, ja sit se vaan kysy, et mistä sinä tiedät, ja... mä vaan sanoin, et mä tiedän kyllä.

[Haastattelija ja muut puhujat nauravat 00:23:15]

Puhuja 4 [00:23:19]: Et kyl mä luulen, et potilas näkee sen myös, sen korkean ammattitaidon, ammattiosaamisen, ja vastuullisuuden siellä kaikkien työntekijöiden osalta. Et se kyl tulee näkymään.

Puhuja 2 [00:23:38]: Niihän mullakin oli eilen siellä Malmin sairaalassa kokemus. Mä kävin siel päivystykses. Sydän muljahtelee tässä ja kauheen inhottava olo, ja mulla on nyttekin vähän semmonen olo, niin tota... ne oli vaan siellä, kokeili pulssia ja otti paineet, ja sano että eei, eei, mene kotiin, työterveyden kautta sitten, ei ei ei, ei sul oo mitään hätää.

Haastattelija [00:24:03]: Niin, et ne laatupoikkeemat tulee kyl...

Puhuja 2 [00:24:04]: ...kiitos tästäkin, verorahat kyllä kelpaa, mut sit ku [ois halunnu? 00:24:07] hoitoo, ni sit on niinku et mitä sä tänne tuut.

Puhuja 1 [00:24:09]: Joo, kyllä.

Haastattelija [00:24:12]: No miten te aattelette...

Puhuja 1 [00:24:13]: Niin, ja sen mä vielä niinku tosta näin, että olin itekin sairaalassa kolme päivää kesällä, ni mä kiinnitin huomiota tähän tietosuojaan. Et se ei toteudu millään lailla. Ei kertaakaan.

[Puhujat ja Haastattelija myöntelevät 00:24:23]

Puhuja 1 [00:24:24]: Se on must ihan kauheeta, ja nyt kun ollaan niissä EU-, tää standardi mikä on, ja muutenkin, ni siihen pitäs kiinnittää huomioo, ja aivan surutta puhutaan henkilötunnuksella, nimellä, sit ku on siellä esipäivystyksessä ni siellä kuulee, ku joku puhuu ihan ummet ja lammet. Ni must se oli jotain ihan järkyttävää.

Puhuja 4 [00:24:42]: Joo.

Puhuja 2 [00:24:43]: Joo, ihan sama.

Puhuja 3 [00:24:44]: Hämeenlinnan sairaalassa mun äiti joutu... ihan iso potilashuone, ja kaikkien potilastiedot siellä laplatettiin ääneen. Kaikkien kohdalla, laaa, kovalla äänel et varmasti kaikki kuulee. Mun äiti oli ihan, et voi hyvänen aika, ettekste yhtään ajattele, et ehkä joku ei halua et tää asia...

[Puhujat myöntelevät 00:24:59]

Puhuja 3 [00:25:02]: Siellä vaan.

Puhuja 1 [00:25:05]: Että tietosuojan voit sinne laittaa.

[Puhujat nauravat ja myöntelevät 00:25:05]

Haastattelija [00:25:07]: Kyllä, se on kyl tärkee. Tota...

Puhuja 1 [00:25:10]: Vaikka kyllä mun mielestä täällä hyvin jo sitä, täällä noudatetaan jo nyt.

Puhuja 4 [00:25:13]: Kyllä.

Puhuja 1 [00:25:14]: Mut et siihen entistä enemmän voidaan kiinnittää.

Haastattelija [00:25:19]: Joo, toi on tärkee pointti. Miten sitte, ku näätte, ku teil on nyt kaikilla tämmönen tuore kokemus tosta potilaana olosta, niin miten te, onks teil ollu mitään mahollisuutta antaa, raportoida niistä laatupoikkeamista?

Puhuja 3 [00:25:30]: Niin. Ois voinu ehkä ensin sanoo jotain. [Eilen oli oma olo niin paha sillon? 00:25:32]

Haastattelija [00:25:33]: Tosi hyviä kokemuksia tän talon kehittämises, ku ite menee, ni et ei ainakaan noin kannata toimia.

Puhuja 4 [00:25:42]: Siis se on niin eri kulttuuri siellä. Mä voin sanoo, esimerkiks aamulla... Mä olin koko viikon syömättä. Ensimmäiset kaks päivää mut unohdettiin panna leikkausjonoon. Sit kolmantena päivänä mä pääsin niin kutsuttuun oranssiin jonoon, ja sitten perjantai-iltana kello 22 tuli kirurgi sanoon, et nyt leikataan. Ja... elikä mä en ollu saanu ruokaa koko viikkona, kaikki meni kirkkaana tippana. Sit oli se lauantai, mä sanoin, et nyt mä lähen kotiin, ja sain luvan siihen, mutta sitten oli kauhee nälkä tietysti aamulla. Ni sielt tuli sitten sairaala-apulainen, joka... se ei oo se Peijaksen aamiainen, mikä on hirveen iso, siin oli yks pieni leipä ja hyvin pieni jugurtti, ja sit oli kahvi. Mä sanoin, et mä ottasin mieluummin teetä. Ni hän sano, et ei voi antaa. Siis siel on kaikki näitä, ei oo suomenkielisiä nää hoitajatkaan. Mä kysyin siltä, et mitä varten? Paperissa lukee. Eikä se tuonu teetä mulle. Siis jos meillä tämmöst tapahtuis, niin kyllähän siitä ottas esimies heti...

[Puhujat myöntelevät 00:27:09]

Puhuja 4 [00:27:13]: Mut must tuntuu, et se on niin kaukana tää, et meillä me tehdään tätä kyllä, mun mielestä tää laatujärjestelmä määrittää sen hyvän asian, et me tehdään tämä ja me erottaudutaan tästä meiän suurimmasta kilpailijasta sen kautta. Et se on järkyttävää nähdä, miten, minkälaista se on, ja kannattaa mennä kyllä ei-sairaanhoitajana, ei-siivoojana, eikä kuntoutuksen asiantuntijana. Sillon on hyvä seurata sitä. Tietysti on poikkeuksia...

Puhuja 3 [00:27:44]: Niin, tai omaisenakin olla siellä, et tietää. Mut toisaalta se on hirveen hyvä kokemus ittelle sitten. Sä näet, kun sä tuut töihin itse, niin sä muistat ne.

Puhuja 4 [00:27:55]: Kyllä.

Puhuja 2 [00:27:59]: Kyllä.

Puhuja 3 [00:28:00]: Et miten käyttäydyt, mitä sanot, mitä puhut.

[Puhujat myöntelevät 00:28:03]

Puhuja 3 [00:28:05]: Et se luottamus ja yksityisyys on äärimmäisen tärkeitä.

Haastattelija [00:28:18]: Onks teillä jotain muita asioita, mitä teiän mielestä pitäis tähän laatujärjestelmän käyttöönottoon ottaa huomioon, tai mikä on niinku terveisiä, mitä te haluutte viedä?

Puhuja 1 [00:28:26]: Resurssit.

Haastattelija [00:28:28]: Mitäs niistä?

Puhuja 1 [00:28:30]: No tuntuu, että monet kyllä päätänsä, hiuksia repii täällä, et missä ajassa, millon tehdään näitä. Se on semmonen...

Puhuja 2 [00:28:40]: Jos meist on ykski pois, niin meil on heti hätä, se koko laitoshuolto. Sinikka repii tuol ihan hiuksia päästä. Mitä, mistä, mihin, mihin, mihin.

Puhuja 1 [00:28:48]: Mm, mut et tämmösiä kun rakennetaan, ni...

Puhuja 2 [00:28:49]: Mä pistin eilen sille viestin, että mä meen nyt päivystykseen, ni se oli ihan jo heti, mitä nyt, iltavuoroon ei tuukaan...

Puhuja 1 [00:28:59]: Pitäs myös resurssoida se... aika ihmisille, jotka sitä rakentaa.

Haastattelija [00:29:06]: Mm. Onko muita asioita, mitä te haluisitte, että erityisesti kiinnitetään huomiota?

Puhuja 4 [00:29:14]: No ehkä sit liittyen tohon resursointiin, voi ajatella sitä, että ehkä se nyt nykyään onnistuu, me ollaan kuitenkin varsin pieni yksikkö, niin sillon jotenkin tuntuu, et se on ihan luonnollista, et kaikki voi tehdä kaikkeekin. Elikkä tos oli muutama vuos sit joku, joku toi tänne pissanäytettä, ja sano sen jolle, että minne hän voi tän jättää, ni hän vaan mulle tuli sit, kun mä tunsin tän potilaan, sanomaan että siel ei... et miten te voitte ajatella, et potilas tulee uudestaan, kun tää teidän työntekijänne ei suostunu viemään tätä minnekään. Se olis vaan pitäny laboratorioon viedä. Että mun mielest semmonen... semmonen yhteenkuuluvaisuus, et nää työpaikat on kaikki riippuvaisia siitä, miten me menestytään, ja sit me hoidetaan tätä.

[Puhujat myöntelevät 00:30:08]

Puhuja 2 [00:30:10]: Joustavuutta.

Puhuja 4 [00:30:11]: Siinä mä uskon, et Eiran sairaalas on sellanen henki, jotenkin siel on ehkä enemmän aistittavis tää, et kaikki hoitaa kaikkee, ja et ei oo sellasia määrätynlaisia ammattinimikkeitä. Vaan että sen tilanteen...

Puhuja 1 [00:30:31]: Aivan. Tulee kohalle, käyttää maalaisjärkee.

Puhuja 2 [00:30:34]: Nii. Just näin, joo. Meilläki ku tulee potilas aamulla leikkaukseen ja tulee osastolle, ni kuka tahansa meistä, ainaki sillon ku mä oon työvuorossa, mä oon heti että huomenta, hei, ootko tulossa leikkaukseen, tonne käytävän päähän hoitajalle. Ettei heti, ettei jätetä potilast siihen, se voi olla ekaa kertaa täällä. Okei, jos se on viidettä kertaa, se tietää mennä, mut se on ihan kiva huomioida, että...

Puhuja 4 [00:30:56]: Tulee heti hyvä mieli siitä.

[Puhujat myöntelevät 00:30:58]

Puhuja 1 [00:30:59]: Se on se kontakti, että miten me kaikki täällä suhtaudutaan potilaisiin.

Puhuja 2 [00:31:07]: Jokainen voi tehä sen. Se on parasta asiakaspalvelua mun mielestä, huomioida toinen ihminen.

Puhuja 4 [00:31:13]: Mä sanon yhen esimerkin siitä. Nyt mä olin sitte tossa (…), elikä tuolla Meilahdessa, ni mä ihmettelin sitä, että mulle oli annettu sit semmonen jälkikontrolliaika, joss oli semmonen toimenpide kyllä. Ne oli toimenpideaikoja, ja siel oli ehkä, meitä oli ehkä 15 potilasta siinä, ja mä tulin jo sen verran aikasin, et mä kävelin siitä, niin siel oli... mun tuli mieleen ihan tää aikasemman Finnairin tämmönen lentoemäntä, joka ottaa vastaan potilaan, tota, matkustajan siellä odotusaulassa ja hoitaa kaiken sille. Täs oli semmonen erittäin kielitaitonen - siel oli paljon ulkolaisia potilaita myöskin - semmonen kielitaitonen henkilö, joka pani järjestykseen. Ensinnäkin tuotiin nää maskit, ja sitte sano, et nyt on valitettavasti kirurgi matkalla, mutta vähän myöhästyy, mutta ei tää yhtään myöhästy tää asioiden kierto. Eri kielil, puhu myös ranskaa ja englantia siinä, muistaakseni ei muita kieliä. Mut kaikki niinku tiesi sen siinä, et miten on. Sitten mä huomasin, et sit mä menin - ku olin niin aikasin, mut laitettiin ensimmäiseks - mä menin sinne saliin, ni hän on siellä sitten pukeutuneena, hän on sairaanhoitaja, joka rupee puuduttamaan mua ja toimii siinä assistenttina kirurgille, ja kaikki yks henkilö huolehti. Et siel ei oo enää tätiä siellä kopissa, vaan siel oli henkilö, joka otti sen koko 15 potilasta, kaikki huolehti niistä. Ja kirurgi oli tietysti. Ne kaksisteen teki sen työn siinä. Mä en oo tämmöstä ennen Meilahden sairaalas nähny. Et mulle jäi semmonen hyvä mieli siitä, että olipa hieno.

Haastattelija [00:33:19]: Ja varmaan enemmänki semmonen persoonallisuuskysymys häneltä, että hän otti sen tilanteen haltuun.

Puhuja 4 [00:33:24]: Kyllä. Joo. Mut semmosia ekstroverttejä ihmisiä on meilläkin varmaan, jotka pystyy sen tekemään, ja joil on ammattitaitoo siinä. Et se on semmonen hyvä, tulee semmonen hyvä mieli siitä, et ei tartte mennä niiden koppien luokse ja kysyä.

Puhuja 3 [00:33:42]: Kyllä.

Haastattelija [00:33:49]: No miten tän vois ottaa tässä laatujärjestelmässä huomioon? Tommost kokonaisvaltasta, voiks teiän mielestä laittaa tämmöseen laatujärjestelmään, että ihmisten vastaanotto ja...?

Puhuja 2 [00:34:03]: Miksei.

Puhuja 3 [00:34:04]: Miksei vois.

Haastattelija [00:34:06]: Ni, on ihan selkee laatutekijä.

[Puhujat myöntelevät 00:34:08]

Puhuja 3 [00:34:13]: Just se, et niinku sä sanoit, ihminen nähdään. Se huomioidaan, et se on täällä, ja oli sitten mikä tilanne tahansa, niin sille voi sanoa muutaman sanan ja käydä vaan. Se informaatio sille asiakkaalle on varmasti hirveen tärkee asia. Vaik ois myöhässä tai mitä tahansa, niin kun he tietää, ni se on ihan eri asia. Jos he ei tiedä, et he vaan istuu, niinku itse on joutunu omaisena istumaan... se oli toista tun-, yli kaks tuntii myöhässä lääkäri, ja kukaan ei tullu kertomaan mitään, mis mennään. Ni kyl se oli vähän semmonen, et lopputulos oli, että vaihdettiin lääkäriä.

[Puhujat myöntelevät 00:34:59]

Puhuja 3 [00:35:00]: Mut se, että se ois helpottanu hirveesti, jos joku ois vaan nähny mut, et hei, te odotatte täällä pitkään. Et tilanne on tämä.

Puhuja 2 [00:35:10]: Niin, tulla sanomaan, että tilanne on tämä.

[Puhujat myöntelevät 00:35:11]

Puhuja 3 [00:35:15]: Ite tekee sitä samaa täällä, ni ihmisten reaktio on ihana, et helpompi odottaa ku tietää.

Puhuja 2 [00:35:22]: On, on.

Puhuja 3 [00:35:24]: Ja voi yrittää arvioida, ja se ei välttämättä aina mene oikein, mutta...

Puhuja 2 [00:35:31]: Mut sinne päin.

Puhuja 3 [00:35:32]: Joo. Mut heidät on huomioitu.

Puhuja 1 [00:35:34]: Nii, ja että on että okei, te ette oo unohdettu.

Puhuja 3 [00:35:37]: Joo, nimenomaan se, ettei oo unohtunu.

Puhuja 2 [00:35:40]: Ja sillon [?? 00:35:39] oli siin neloskerroksessa, siinä A-käytävällä, niitähän piti monta kertaa ihan, eeei, ku tästä nyt menemme, ku tuli ihan sellasia, et missä se on se, et niinku melkeen ottaa käsikynkkään, et nyt mennään yhdessä ja näytetään tästä [?? 00:35:56] neloskerros. Sit ne tuli osastolle...

Puhuja 3 [00:36:00]: Sama se on tos alakerrassa. Siel on koko ajan, sä näät heti ihmisestä, et tää on aivan eksyksissä. Ei tartte muuta ku pysähtyä, et hei, mitä etsitte. Ni se on sillä hoidettu.

Puhuja 4 [00:36:16]: Ja sit se, et tietysti sillon kun hän panee sairaalan oven kiinni, ni se hoito ei suinkaan pääty siihen, vaan se huolenpito jatkuu vielä sen jälkeenkin, ja hänel on selvät speksit, et miten, mihin hän ottaa yhteyttä. Sekin on sellainen turvallisuutta lisäävä tekijä, ettei hän jää ihan omilleen sitten.

[Puhujat myöntelevät 00:36:39]

Puhuja 3 [00:36:40]: Just tänään sain palautteen siitä, ku oli pieni toimenpide tos alhaalla, ja sitte seuraavan kerran hän tulee perjantaina, ja sitte kun mä sanoin, et jos tulee ongelmii, ni meille voi aina soittaa, voi tulla näyttään, et ei sen oo pakko olla sillon perjantaina vasta, ni sit se itse asiakas sano, no tämä oli niinku et nyt on turvallinen olo lähteä pois. Et ku tietää, et mä pääsen takasin, mä saan yhteyden, ja mä saan apua jos mä tarvitsen.

Puhuja 2 [00:37:15]: Et just tää, et iteki aina, tai monta kertaa sanoo, et hei, voit soittaa mulle myöhemmin, jos tulee jotain. Harvemmin soittaa, mutta se, et näkee et ne on siitä hyvillä mielin, et okei, voi olla yhteydessä.

Puhuja 3 [00:37:30]: Se on niin helppo antaa se puhelinnumero, ja sit niil on, se on tärkeää et minulla on nyt tämä näin, et minä voin soittaa.

Puhuja 2 [00:37:41]: Joo, ja semmonen turvallisuuden kokemushan on jotenki tosi tärkee.

Puhuja 3 [00:37:51]: [hiljaa taustalla] Sen takia se työpuhelin on.

Haastattelija [00:37:55]: Hei, onks meillä jotain, onks jotain vielä mitä pitäis lisätä tai haluisitte kommentoida, tai...?

Puhuja 1 [00:38:04]: Niin, sitä aattelin sanoo vielä, et sitten tärkeetä, et se tulee sitte se laatujärjestelmä semmosella... normi, ymmärrettävällä kielellä, että siin ei niinku oo sellasta kapulakieltä tai jotenki semmosta, mistä vois tulkita niin tai näin tai... Tai sit tietysti siin on niin monta eri kohtaa, mut kuitenki.

Haastattelija [00:38:26]: Toi on tärkee pointti, koska monesti näissä laatujärjestelmissä käytetään jotenki semmosta laatukieltä, puhutaan akkreditaatiosta ja puhutaan semmosilla käsitteillä tai muita, niinku just laatupoikkeamat, et ensin pitää määritellä ne käsitteet ennen ku sit ku päästään itse siihen... et ihan niinku omaki kieli, tota, pyörii näissä kyllä helposti. Hyvä pointti. Et puhuttas sillä kielellä, mitä se työ on, ja muokattas se teidän työpaikan mukasesti eikä...

Puhuja 2 [00:38:57]: Niin, ja siihen pääsee nopeesti sitten [?? 00:38:58].

Haastattelija [00:39:03]: Olisko muita, mitä pitäs vielä huomioida tai mitä tulee vielä mieleen, mitä en oo hoksannu kysyä?

Puhuja 3 [00:39:11]: Ei, en mä ainakaan nyt osaa.

Puhuja 2 [00:39:15]: Mul on nyt vähän tää mun olo semmonen, en ihan täyspainosesti tässä voi, sellanen hassu olo mulla. Mut en mä, ei mulle tuu mieleen nyt, että.

Haastattelija [00:39:34]: Onks muilla jotain mitä halutaan, vai lopetellaanko tähän? Täs tulikin monta tärkeetä kohtaa, mitä pitää ottaa huomioon. Onks jotain mitä haluutte täydentää tai kysyä?

Puhuja 3 [00:39:51]: Se mikä oli, niin se, et ei noi tietenkään hetkessä synny.

Haastattelija [00:39:55]: Nii.

Puhuja 3 [00:39:56]: Et kyl niitä pitää miettiä ja rakentaa, ja pelkkä taustatyö on jo oma asiansa, ja sit saatika rupee niitä kirjottamaan sitten johonkin muotoon, ni kyl se on pitkä prosessi synnyttää nämä.

Haastattelija [00:40:21]: Ootteks te nähny valmiina semmosii laatujärjestelmän kuvauksia tai niitä, mitä kaikkia on nyt kirjotettu, että... ku mietin, et jos ois joku malli vaikka jostain muusta sairaalast tai muista vastaavista. Ku niitä standardeitahan kuitenki on olemassa erilaisia, et nää asiat ainakin pitäs huomioida.

Puhuja 4 [00:40:47]: Kyllähän tää korostuu nyt tällä hetkellä, koska meidän potilaat, asiakkaat, tai kuntoutujat ymmärtää lääketieteestä niin paljon enemmän kun aikasemmin. Ja tällä me pystytään varmaan erottautumaan, ja se on tärkee pointti, että kyl ne usein tietää jo suurin piirtein, et mitä se lonkan tai polven protetisaatio tarkottaa. Mut sit täytyy olla paljon muutakin, joka tekee siit semmosen kokonaisen, hyvän kokemuksen täällä. Et me ei olla enää semmosessa ammatissa, et me voidaan sanoo, et kyllä lääkäri sen kertoo, vaan ne tietää jo etukäteen.

Puhuja 2 [00:41:33]: Ihmiset osaa googlata.

[Puhujat nauravat ja myöntelevät 00:41:34]

Puhuja 1 [00:41:40]: Juu, ja sit googlaa vielä sen pahimman vaihtoehdon.

Puhuja 2 [00:41:44]: Jos sellanen tulee esiin. Sehän on yleensä hyvin...

Puhuja 3 [00:41:50]: Joskus saa vähän toppuutella, et ei ihan kaikkea pidä lukea.

Haastattelija [00:41:55]: Joo, mut selkeesti toi on vaikuttanu teidänki työnkuvaan, että...

Puhuja 4 [00:41:59]: Joo, kyl se on ihan. Ja sitte toisaalta, meidän kaikkien täytyy seurata aikaamme, eikä me voida olettaa siihen, että ne 20-30 vuotta sitten opiskeluaikana, et ne

pätis edelleen, vaan meidän täytyy koko ajan lukee sitä ja seurata. Varmaan kaikissa meiän toimipisteiden ammateissa on tää sama, että itsensä elinikänen kouluttaminen.

[Puhujat myöntelevät 00:42:31]

Haastattelija [00:42:36]: Onko teillä annettu, pystytteks te tekemään sitä töissä kaikissa ammateissa, vai meneeks se sitten, no...

Puhuja 3 [00:42:46]: Sanoisin, et välillä pystyy ja välillä ei. Välillä sun täytyy kyllä ihan muulla ajallakin käydä läpi ja selvittää itselleen asioita.

Puhuja 1 [00:42:58]: Nii, koulutuksiin pääsee tosi huonosti, että...

Puhuja 3 [00:43:00]: No nyt on nykyään. Ei oo enää itsestäänselvyys koulutukset.

Puhuja 1 [00:43:04]: Sekin on yks tietenki.

Puhuja 3 [00:43:07]: Mut kyl sit täytyy vähän niinku seurata sitä, et mitä tänä päivänä tapahtuu, ja...

Puhuja 1 [00:43:12]: Totta kai. Ei oo muuta vaihtoehtoo. Joskus aikoinaan, kun...

Puhuja 3 [00:43:17]: Jostain tietyistä asioista sit pitää itselleen vaan selvittää ne, että miten nämä toimii, mitä tämä tekee.

Puhuja 2 [00:43:27]: Meilleki tulee opiskelija, me ollaan heti, tai mä oon ainakin heti, no, mitäs uutta, mitäs uutta, onks jotain uutta? Koska nyt on jääny ku nalli kalliolle vähän, että mäkin oon ihan laitoshuoltaja. Käyny sen kurssin tai sen koulun, mikä se nyt on. Ois tietysti kiva, nyt on menny jo eteenpäin se ala.

Puhuja 1 [00:43:55]: Kaikki alueet menee eteenpäin, joo. Muuttuu.

Puhuja 3 [00:44:01]: Ja huomaa mun mielest sen, et entist enempi tarvitsee sitä eri... niinku ryhmien asiantuntijuutta. Tulee mieleen just sinut esimerkiks, ni kyllä hyvin helposti huomaa, että enpä tiedäkään niin paljon, ja en pysty neuvomaan, mut onneks sitten on alan ihminen, joka on oikea siihen, ja... et hyvin monessa tilanteessa tulee sitä, et eri asiantuntijat tarvitaan.

Puhuja 2 [00:44:33]: Niin, kyllä.

Haastattelija [00:44:33]: Niin, et tämmönen moniammatillinen yhteistyö.

[Puhujat myöntelevät 00:44:36]

Puhuja 2 [00:44:38]: Se on oikeestaan tän talon vahvuus. Tai on.

Puhuja 3 [00:44:39]: Joo, ja se, et saa helposti, et kun tarvitaan fysioterapeuttia tai tarvitaan sosiaalityöntekijää, tai ihan mitä tahansa, niin se, että sä löydät sen ihmisen, se niinku onnistuu. Sit jos sul on isompi byrokratia, iso laitos takana, ni sieltä se ei tuukaan niin nopeesti.

Puhuja 2 [00:45:01]: Et sitähän noi paljon kuntoutujatkin arvostaa, et kaikki ammattilaiset on saman katon alla ja saa niinku just...

[Puhujat myöntelevät 00:45:09]

Puhuja 2 [00:45:10]: Mut paljon he joutuu juoksemaan luukulta luukulle ja asiantuntijalta asiantuntijalle.

Puhuja 3 [00:45:19]: Mut ihan samalla tavalla tarvitaan lääkäriä, ja sit lääkäri tarvitsee hoitajaa, ja tarvitaan fysioterapeuttia et saadaan tämä ja tämä puoli tehtyä, ja... kaikilla on sitte omansa.

Haastattelija [00:45:34]: Mm. Miten se moniammatillisuus vois näkyä tässä laatujärjestelmässä? Niinku säki, sehän on selkee laatutekijä tässä talossa, et se on saatavilla, ni...

Puhuja 4 [00:45:53]: No mä sanon, et esmes mun työssäni semmonen yks muoto on... sanotaan nyt olisko se ollu viime viikolla, elikä maksakirroosihan on hyvin vaikee diagnosoida, ja me saatiin tehtyä se diagnoosi, ja sit pyydettiin, siin oli anestesialääkäri ja sisätautilääkäri ja ortopedi yhdessä, ja me pyydettiin potilas ja hänen miehensä sisään ja käytiin läpi tää maksakirroosiin liittyvät sudenkuopat leikkauksessa ja suunnitelma siitä, et miten tää tehdään niin, että hänelle mahdollisimman vähän syntyy leikkausriskejä ja turvallisesti voidaan lonkka protetisoida. Ja mä uskon, että nää asiakas ja hänen puolisonsa olivat erittäin kiitollisia siitä, et siinä oli kolme lääkärii, saatiin samaan istuntoon, ja jokainen katto sen omalta puolelta, ja sit tehtiin siitä tämmönen yhteenveto vielä. Ja se ei vaatinu aikaa, kun kaikki tehtiin puolessa tunnissa.

Puhuja 1 [00:46:58]: Ja tätä varmasti arvostetaan siellä.

Puhuja 4 [00:47:03]: Joo. Ja täytyy sanoo, et ei varmaan julkisella puolella tällästä pysty tarjoamaan.

Puhuja 1 [00:47:10]: Ei pysty. Että ihan se oma kokemus mikä on, että kun on kuntoutuja, ni sitte he käy yhellä lääkärillä yhen asian takia, toisella toisen, kolmannella kolmannen, et ei oo mitään semmosta. Ja sit ne jää kauheen yksin. Ei niinku oo... niin toi onki tosi hyvä.

Puhuja 3 [00:47:35]: Se on se pienen talon etu.

Puhuja 1 [00:47:43]: Ja myös se, et haluu siihen panostaa, tämmöseen.

Haastattelija [00:47:48]: Nii, ja kohdataan se potilas kokonaisena, ettei hoideta vaan sitä lonkkaa, vaan hoidetaan koko ihmistä.

Haastattelija [00:47:58]: Oliks...

Puhuja 2 [00:47:58]: Kyllähän tuol osastollaki välillä nää potilaat on ihan hauskoja, ku ne sanoo, et ai, nyt pitää lähtee kotiin, voi voi. Vähän niinku silleen haikeena [naurua 00:48:09]. Tääl on nii hyvä ruoka ja kaikki on niin ystävällisiä ja kivoja. Yleensä se on toiste päin. Kylhän sekin kertoo jotain aika paljon.

[Puhujat myöntelevät 00:48:25]

Puhuja 3 [00:48:27]: Mä itse tein julkisella puolella pitkään töitä, ja sielläkin löytyy eroja. Pienemmistä taloista, missä asiat toimii hyvin, ja sit on nää jätit, ja mulle varmaan kyllä oli suurin syy, miks mä halusin pois sieltä, ni se, että ku siellä se yks ihminen hukkuu. Ja sit et kukaan ei ottanu sillä tavalla vastuuta siitä, ja niinku hoitanu sitä, et se oli aina just tää, et hoidettiin vaan tämä palanen tai sitte tämä palanen. Se oli aika avutonta olla sit siellä potilaana. Se oli mun mielestä, mä en koskaan itse sopeutunu siihen, must se oli väärin, tai outoa. En tykänny siitä. Ja kuinka helpottu heti kun tuli pieneen taloon, ni asiat hoituu ihan eri tavalla.

Haastattelija [00:49:38]: Hyvä. Jos ei muuta, niin mä kiitän teitä lämpimästi. Täs tuli monta hyvää pointtia, ja tästä on hyvä jatkaa tän laatujärjestelmän kehittämistä ja käyttöönottoo, kun tietää vähän, että mikä se lähtötaso on tässä tilanteessa. Kiitos kovasti teille kaikille.

[taustahälyä, puhujat poistuvat 00:49:59]

Haastattelija [00:50:11]: Me siirrytään tästä pois.

[recording ends]

Name of recording: Haastattelu2.mp3

Length of recording: 00:54:31

Information:

[?] = Word was not understood completely but meaning is almost correct. Recording point is written to the text e.g. [word? 00:15:44]

[??] = Word could not be understood and therefore could not be written. Recording point is written to the text e.g. [?? 00:15:44]

[text] = Sounds or not transcribed parts are written with square brackets e.g. [laughs] or [interview paused due to a phone call]

[recording starts]

Puhuja 1 [00:00:00]: Järjestelmän käyttöönottoon täällä. Mikä teidän kokemus on tästä asiasta yleensäkin?

Puhuja 2 [00:00:11]: Mä voin ensin sanoa, että ei oo hirveesti kokemusta täällä rintamalla, oon kuullu et on tämmösiä tehään ja suunnitellaan, on eri ryhmiä sitten, kuka käy läpi sitä prosessia, mutta ei sen kummosempaa.

Puhuja 1 [00:00:32]: Entäs Sanna?

Puhuja 3 [00:00:33]: Sama juttu, olen kuullut mutta mitenkään en oo lukenu lisää tästä aiheesta tai sen isompaa.

Puhuja 1 [00:00:44]: Joo. Ja sulla oli kanssa että?

Puhuja 4 [00:00:46]: Aika epämäärästä vielä tässä vaiheessa tää tuntuu olevan, enkä oo perehtyny. Sen verran kädet ollu savessa, että mä oon ollu vähän ylikuumettunu jo aika pitkään, ehtiny näitä oheisjuttuja tarkastella. Perustöissä ja tutkimuksissa mun aika.

Puhuja 1 [00:01:09]: Onks teistä kukaan osallistunu näihin laatujärjestelmään liittyviin koulutuksiin tai orientaatioihin mitä tähän on? Tai työryhmiin? Ei?

Useita puhujia [00:01:19]: Ei.

Puhuja 1 [00:01:22]: Nyt kun tähän on varattu aikaa miettiä tätä laatujärjestelmää, niin mitä odotuksia teillä vois olla tän laatujärjestelmän käyttöönottoon liittyen? Mihin te assosioitte sen?

Puhuja 3 [00:01:37]: Mun mielest se, vuodeosastolla olen töissä, asiakaspalvelun kehittäminen ja että potilaat, siis on ne tyytyväisiä, mutta miten ne täyttää semmosen lomakkeen lähdössä--

Puhuja 1 [00:01:59]: Palautelomakkeen.

Puhuja 3 [00:01:59]: Niin siellä kaikki on hyvin.

Puhuja 1 [00:02:05]: Miten sä aattelit että laatujärjestelmä vois auttaa siihen?

Puhuja 3 [00:02:10]: Kehittää niitä osia mistä potilaat on ei tyytyväisiä.

Puhuja 1 [00:02:24]: Kyllä.

Puhuja 2 [00:02:28]: Mä ite oon poliklinikalla ja ehkä se tois kans parempaa asiakaspalvelua et asiat sujuis joustavammin, et kaikki tekis samalla tavalla, toimittas samalla tavalla, olis kaikilla samat säännöt, ehkä se veis eteenpäin, parempaan suuntaan.

[Ovi aukeaa 00:02:59]

Puhuja 1 [00:03:00]: Hei.

Puhuja 5 [00:03:04]: Päivää, olen pahoillani myöhästymisestä.

Puhuja 1 [00:03:04]: Ei hätää.

Puhuja 6 [00:03:07]: Mies (…) saakka.

Puhuja 1 [00:03:08]: No ni. Mä oon siis (…) ja teen tutkimusta laatujärjestelmän käyttöönotosta.

Puhuja 5 [00:03:20]: Joo, mä annan tämän suostumuslapun tästä.

Puhuja 1 [00:03:25]: Elikkä saa nauhottaa? Kuten selitin jo muille, käytän tätä tietoa tähän tutkimukseen ja kehittämiseen ja myös jaetaan anonyyminä, eli voi jutella ihan rauhassa ja poistetaan kaikki henkilötiedot ja tai kaikki mihin pystytään liittämään teidän sanomiset teihin henkilöihin. Haastattelun saa keskeyttää milloin haluaa ja tää on muutenkin täysin vapaaehtoista, että teitä ei kukaan pakottanu tänne. Istuksää johonki silleen et pysyy vähän turvaväliä? Vaikka siihen ni mä kirjotan samalla tukimuistiinpanot, jos tolle nauhalle tapahtuu jotain ni meidän ei tarvii tehä uudelleen vaan on sentään jotain muistiinpanoja olemassa. Mutta kysyin muilta jo, miten ootte osallistunu laatujärjestelmän käyttöönottoon, onko sulla millanen kokemus tähän mennessä?

Puhuja 5 [00:04:20]: Ei oikeestaan mitään.

Puhuja 1 [00:04:23]: Hyvä, kun tää on tällänen nollatutkimus, selvitetään nollatasoa, et mikä on lähtötaso miltä tää otetaan käyttöön. Ootko osallistunu laatujärjestelmään liittyviin koulutuksiin tai orientaatioihin? Infoihin?

Puhuja 5 [00:04:36]: Sillon ihan alussa oli jotain muutama, mutta ne kokoukset on aina perjantaisin jollon mä olen poissa täältä muissa tehtävissä ni en ole osallistunu.

Puhuja 1 [00:04:48]: Me oltiin puhumassa siitä, et minkälaisia odotuksia teillä on laatujärjestelmän käyttöönottoon liittyen, puhuttiin jo että vois tulla parempaa asiakaspalvelua, joustavampaa toimintaa ja palautelomakkeessa on tullu ilmi että kaikki asiat ei oo välttämättä niin hyvin mitä haluttas olla, et se vois auttaa semmosiin asioihin, oisko sulla jotain muuta mitä haluaisit lisätä ennen kun jatketaan?

Puhuja 5 [00:05:13]: Mä kuulin siitä, luin JCI-käsikirjan ja sitten oli (…) laatuihminen puhumassa vastaavasta hankkeesta (…) ja totesin että se sellaisenaan se on meillä JCI-formaatissa äärimmäisen vaikee viedä täällä läpi, pitäs olla päivystävä sairaala, jossa ois päivystävän sairaalan resurssit, mutta periaatteessa se, että me tehään tätä laatutyötä, se on hyvä asia, suhtaudun positiivisesti mutta en usko, että päästään siihen tarkkuuteen mikä JCI:ssä on.

Puhuja 1 [00:05:55]: Sen takia että tää ei oo päivystävä sairaala?

Puhuja 5 [00:05:58]: Sen tähden, että se on niin kaikenkattava homma, musta ois realistista, että meillä ois ihan sitä varten palkattu ihminen, joka sitä tekis, ottas vastuun siitä projektista. Se, että me tehään sitä ryhmässä ja vähän oman työn ohessa, koska se on aika niukka panostus siihen.

Puhuja 1 [00:06:28]: Et kun se on työlästä tehä?

Puhuja 5 [00:06:31]: Se on työläs ja ihan järjettömän iso projekti, jos sitä käsikirjaa lukee. Mistä kaikesta alkaen pitäs laatu määritellä. Se on vähän semmonen amerikkalaisista lähtökohdista lähtevä, jossa pitää määritellä minkälaista sähköä seinästä tulee ja miten sen laatu on varmistettu. Se on aika iso vaatimus. Eli olen hiukan tässä suhteessa negatiivinen JCI:tä kohtaan, mutta periaatteessa kannatan laadun prosessien hiomista.

Puhuja 1 [00:07:05]: Jos aatellaan laatujärjestelmiä, ei puhuta JCI:stä vaan laajemmin, sanoit että suhtaudut myönteisesti muuten laatujärjestelmän käyttöönottoon? Minkälaisia odotuksia?

Puhuja 5 [00:07:21]: Mä odotan, siis ylipäätänsä laatujärjestelmä mun ajatuksissa, se että me käydään läpi sitä mitä me täällä tehdään, koitetaan ettiä ongelmakohtia ja parantaa niitä, se on järkevää, se on nähty näissä erilaisissa hoitopoluissa mitä tänne saadaan luotua. Minusta se on kannatettavaa työtä sikäli ja kannatan tätä hanketta sen näkökulmasta.

Puhuja 1 [00:07:52]: Ajatteletteko, kun puhuttiin JCI:stä, mitä muut ajattelette, oisko tästä olla jotain haittaa tämmösen laatujärjestelmän käyttöönotosta? Muuta ku että tulee paljon lisää työtä?

Puhuja 3 [00:08:10]: Ei se varmaan haittaa.

Puhuja 1 [00:08:10]: Et ei oo haittaa.

Puhuja 4 [00:08:16]: Varmaan jotenki prosessoin ajatuksiani silleen, et mun on vaikee lähtee näitä asioita analysoimaan, kun mul ei oo selkeetä kuvaa siit platfomrista millä liikutaan, mä aattelin et on monenlaisia prosesseja jotka on laadultaan ja määriltään hyvin erilaisia, jotenki mä aattelen omaa työtäni ja mä aattelen vaikka varaajien tai sihteerien töitä, ni ei oo kovin paljon kosketuspintaa. Ne on hyvin erilaiset prosessit, jotenki, mut kyl mäkin kannatan niinku (…), kannatan, pitäs olla laaduntarkkailuyksikkö aina jokasessa firmassa, joka miettii

muunmuassa sitä, että voisko samalla effortilla tehä jotain enemmän tai paremmin, voiko säädellä jotain ilman lisäresurssia vai pitäskö saada lisää resurssia johonkin kohttaan, vaikka tehdä asioita paremmin, tavallaan semmonen hyötysuhdeajattelu on jotenki mun syädntäni lähellä. Kyl ne laatuasiat on niin epämääräsiä, jos aattelen omaa työtäni ja potilashoitoprosesseja, niin hyvin vähän on mitattavissa tai edes kovin helposti määriteltävissä olevia asioita, sillon voi kysellä et miten lähtee laatutekijöitä kattomaan tai parantelemaan tai säätelemään. Toki tämmönen vanha konkari kulkee vähän omaa polkuaan, semmosta vanhaa rutiinia on mukana mutta tietenkin. Varmaan ydin on tää, että mun on vaikee sanoo, veikkaan että ne on niin eri asioita, eri kysymyksiä eri prosesseissa. Mut tosiaan tunnen liian vähän tästä. Mä en oo sitä laatukäsikirjaa kattonu.

Puhuja 1 [00:10:30]: Jos aattelette laatujärjestelmää, jos semmonen otettas käyttöön täällä tai kun varmaan otetaan käyttöön, mihin se vaikuttas ensimmäisenä? Minkälaisia tuloksia sen käyttöönotolla vois olla? Mitä te aattelette, mikä tulee ekana mieleen? Onks jotain ilmeistä kohdetta, mihin laatujärjestelmä vois puuttua?

Puhuja 4 [00:11:09]: Ainakin esimiestyöhön, etenkin johtamiseen tai kommunikaatioon, että tieto kulkee molempiin suuntiin. Ylhäältä alas ja alhaalta ylös. Tämmöstä, ainakin se olis semmonen. Asioita kyllä tehdään ja suunnitellaan, esimerkiksi tämä laatujärjestelmä. Nyt kun mä puhun, mä huomaan, että mä suhtaudun tähän vähän sillä tavalla, että tää tuodaan ulkoa, ilman että sillä on tällä hetkellä tän hetken tiedoilla relevanssia mun työn suhteen. Sellanen tulee mieleen. Jos ois kyse, että mitä toivon pari vuotta sitten, jos mä itte olisin keksiny että joku laaduntarkkailu olis hyvä, sillo ois kasvualusta. Tää tuntuu hieman ulkoa, jos se on ameriikoista asti tuotu ni on se aika kaukaa haettu. Konkreettisesti jotenki. Nää on näitä flow-ajatuksia, näitä assosiaatioita.

Puhuja 5 [00:12:18]: Jos noihin assosiaatioihin voi yhtyä, kyllä mä tunnistan itessäni samaa, mä en osaa heti ajatella mitä se meijän toiminnassa ja mitä me tuotetaan, terveyttä asiakkaalle, mun mielestä siinä suhteessa meillä on aika hyvä tilanne, nyt siis konkreettinen laadunseuranta ja tulosten seuranta on olemassa. Prosesseja voi aina parantaa, ehkä mä oon ajatellu sitä siitä näkökulmasta miten saisi asioita menemään turvallisemmin ja tehokkaammin, ja vähemmällä työllä, että sais karsittua päällekäistä työtä, se on ehkä tuotantotaloudellinen näkökulma ollu, mut se on ihan totta että mä koen että meil on tarve saada joku sertifikaatti jota voi käyttää mainostamiseen, että meil on nyt tämmönen leima paperissa, ja siinä on se ongelma että jos me saatas tämmönen järjestelmä, hyväksyntä, ei se mihinkään kertaponnistukseen lopu, se vaatii jatkuvaa työtä että ylläpidetään sitä ja se on vähän niinku Michelin-tähti, ei tule helpolla eikä pysy helpolla.

Puhuja 4 [00:14:01]: Kuulostaa että tää vois palvella sellasta markkinointia, jollain tavalla edullinen asia jolla kilpaillaan vastaavia palveluita myyvien firmojen kanssa et meil on

vuodesta 2020 tai 21 ollu jo tällänen laadunvalvonta ollu meillä, keskustelun tasolla se antaa tiettyä etua. En poissulje sitä, enkä mä oleta, ettei se vois parannella meijän prosesseja, mutta jos tää nyt on motiivi ni se on vähän kaukana kliinisestä työstä. Arjen työstä, kun tavataan potilas tai varataan aikaa tai hoidetaan prosessia ja potilaskuntoutuksessa tai potilatyössä ni ei se sieltä oikeen löydä kasvualustaa tää ajatus.

Puhuja 3 [00:14:52]: Pitäskö tähän, onks eri ryhmistä, fysioterapeuteilta tai muualta, enemmän ihmisiä eri ryhmistä?

Puhuja 1 [00:15:03]: Kyllä joo.

Puhuja 3 [00:15:05]: Pitäs varmaan olla osallistumassa tähän. JCI, onks se sama, on eri ryhmät otettu tavallaan?

Puhuja 2 [00:15:17]: Eiks meil ollu semmosia työryhmiä just, että kuka käy läpi tätä prosessia ja tekee työtä sen eteen et me päästäs joskus jonain päivänä, mutta ku ei meillä oo varsinaista ollut ikinä, pohjatyö on kaikki tekemättä siellä, mehän lähetään ihan nollasta täällä. Jos miettii vaikka kilpailijoilla on käytössä ollut vaikka jo vuosia joku vastaavanlainen systeemi, niin me lähetään niin tyhjästä liikkeelle.

Puhuja 1 [00:15:56]: Tiedätteks te kilpailijoiden niitä, puhuitte (…) et siellä on käytössä JCI, mut onks muilla yksityisilä taloilla kanssa vastaavia järjestelmiä käytössä? Onks kaikissa myös tää JCI vai tiedättekste?

Puhuja 3 [00:16:13]: Sen mä tiedän, että on ja tarkkaillaan laatua, on ihmisiä, jotka tekee sen eteen töitä ja siellä määritellään korkeammalta taholta alaspäin millä taalla työtä tehään, ja on koko talolla yhtenäiset säännöt, tavallaan, missä puitteissa liikutaan.

Puhuja 1 [00:16:43]: Jos miettii arjen työtä, jos laaturjärjestelmä tulee käyttöön, mitä te aattelette mihin se vaikuttas, minkälaisia muutoksia se tois jokapäiväseen työntekemiseen?

Puhuja 2 [00:17:02]: Mä ajattelin sitä, ettei olis semmosta päällekäistä tekemistä, että siinä yks ihminen hoitas potilaan asioita ja se tulis hoidettua alusta loppuun kunnolla ja sit se siirtyy siitä lääkärille tai seuraavalle taholle mutta että ei tarviis, nyt on aika paljon semmosta että ihmiset tekee asioita hirveen paljon eri tavalla, meil on erilaiset käytännöt, me kohdellaan potilaita eri tavalla, ajatellaan potilaista eri tavalla, että otetaan nyt vaikka et

joku on ollu pitkään talossa, hän on etuasemassa verrattuna niihin kuka tulee uutena potilaana taloon. Semmonen jäis siitä. Kaikki potilaat ois saman arvosia on ne sitten ollu pitkään tai tulee vasta ensimmäistä kertaa. Niitä hoidetaan samalla tavalla ja samojen periaatteiden mukaisesti.

Puhuja 5 [00:18:19]: Mä en osaa oikeen nähdä mitään, mitä tekisin toisin. Pidän käytännössä vastaanottoa, tapaan potilaita poliklinikalla, ja sitten hoidan leikkaussalissa, teen leikkauksen, hoidan osastolliset jälkeenpäin. Ehkä siinä vois sanoo, että jotain hyötyä vois olla osastohoidossa, että tietäis jos meillä on semmonen selkeä hoitopolku, niin sillon leikkauksen valmistelu ja leikkauksen jälkeiset asiat menis hyvin ja turvallisesti, toisaalta kun ne menee nyttenkin, siinä mielessä en oikeen näe oisko siinä hirveen isoa muutosta. Vastaanottotyössä en näe, että ois yhtään mitään muutosta. Mutta toisaalta tää on sitä, kun munkin on vaikee hahmottaa et mitä se toiminta tämmösen järjestelmän alla olis, niin en pidä mahdottomana että se vois tuoda jotain sellasta, mitä mä en nyt pysty edes ymmärtämään.

Puhuja 1 [00:19:38]: Aatteletteks te, että tällä olis jotain vaikutusta esim. työturvallisuuteen? Semmosiin kysymyksiin?

Puhuja 3 [00:19:43]: Esimerkiksi vuodeosastolla olisi kiva saada enemmän henkilökuntaa, kun potilasmäärä vaihtelee. Sen suhteen. Sitten potilaallekin on turvallista.

Puhuja 1 [00:20:07]: Vaikuttaako tää teidän mielestä teidän työmotivaatioon täällä?

Puhuja 5 [00:20:15]: Ei ainakaan parantavasti.

Puhuja 1 [00:20:21]: Miten te aattelette laatujärjestelmän ja työhyvinvoinnin kannalta, että onks niillä yhtyttä vai tuoks se vaan lisää töitä ja sitä kautta rasite? Vai aatteletko että se vois vaikuttaa työhyvinvointikysymyksiin? Puhuitte aikasemmin et jos ois yhteiset ohjeistukset, ni sitä vois jotenkin, kaikki tietäs mitä tekis ja työn tekeminen ois helpompaa?

Puhuja 2 [00:20:44]: No omalta kannalta puhun nyt omasta puolesta, ja niitten ihmisten puolesta, jotka tekee sitä työtä mitä itekin tekee, osittain. Ollaan puhuttu siitä ihan keskenäänki, et helpottais kun ne isot päätökset kun ne isot päätökset ja ohjeistus tulis ylhäältä mitä me noudatetaan, eikä niin että me itte sovelletaan jotain tai tehään jotain asioita.

Puhuja 1 [00:21:16]: Osaatko antaa konkreettisen esimerkin?

Puhuja 2 [00:21:24]: No tota. No sanotaan vaikka että vaikka Kela-korvauksista, mitä on kaikilla tiedossa, se on ollu heinäkuusta lähtien aivan täysin kujalla ja ollaan ite puututtu poliklinikan puolella sitten siihen Kela-korvausmenetelmän ja korvauksia ollan annettu vaikka Kelalta niitä ei saada ja on yhteistyökumppaneita joilta ei saada vaikka rahaa sen takia, et Kela ei anna niitä korvauksia ja siellä seisoo sitten laskuja ja siitä on nyt puhuttu ihan johtoa myöten ja yritetty saada selkeyttä siihen että mitähän me tehdään ja millä tavalla me toimitaan ja potilaita tulee kumminkin päivittäin lisää ja lisää, toiminta pyörii samalla taalla. HYK:sin oy:ssä tehtiin se päätös ihan saman tien 1.7. mutta ottanu sen jostaki syystä ei tehty mitään. Asiaa vietiin eteenpäin mutta en tiiä oliko väärät ihmiset asialla. Se on vähän semmosta meijän omaa soveltamista laskutuksen puolella ja joudutaan antaan vastauksia semmosiin mihin meillä ei oo osaa eikä arpaa pystyy vaikuttaa niihin asioihin. Ku viedään eteenpäin niin kukaan ei sano mitään. Vähän pattitilanne.

Puhuja 1 [00:23:21]: Aatteletteko että laatujärjestelmän käyttöönotolla on vaikutuksia sairaalan tulevaisuuteen? Onks tää teijän mielestä semmosta toimintaa, että sairaala jatkossakin pärjää kilpailussa vai et täl ei ois mitään merkitystä siihen liittyen.

Puhuja 3 [00:23:45]: Nyt se on taas niin eri ryhmä potilaita ja eri leikkauksia, ehkä siihen vois henkilökunnalle enemmän koulutusta saada. Sen suhteen.

Puhuja 5 [00:24:04]: Luulen että tää on semmonen investointi tai ajatus, joka varmaan on totta tulevaisuudessa ja siinä mielessä tulevaisuutta ajatellen tätä hommaa täytyy tehdä, mutta se moni asia varmasti hoituis, jsut toi ongelma, että tavallaan tiivistyy Hellevin kuvaamassa ongelmassa myös se Taven kertoma, että tieto ei liiku. Et sillon jos hommat toimis, päätökset tehtäis hyvin, tieto tulisi läpi, luotettavasti, yklä se työhyvinvointia lisäisi ilman muuta. Sillä on positiivinen vaikutus olisi siinä tilantees. Mut se on aika iso kulttuurinmuutos organisaatiossa ja taas se kulttuurimuutos lähtee siitä, että joku sanoo, et nyt tehdään ni ei oo optimaalinen lähtötapa. Se on sikäli hassuu, kun aattelee että meillä on kuitenkin tässä talossa on aina ollu valmiutta muuttaa asioita ja tehdä asioita toisin ja monessa suhteessa me ollaan oltu edellä muita. Monella tapaa. Ehkä tää on meillä alkuinertiaa ja sitä että täytyy päästä käyntiin tän homman kanssa ja näkee että se etenee jossain, et kyl tää tulevaisuutta on mun mielestä. Täytyy, nää asiat on tärkeimpii tulevaisuudessa.

Puhuja 4 [00:25:17]: Mä aattelisin kun kysyit että onks tulevaisuus parempaa tällä niin voi se näin olla jollain tasolla, mutta metatasolla ainakin, ja voipi olla niin että jos tällänen tavallaan, en tiiä onks tää uutta kulttuuria, ainahan me on mietitty miten asioita olaan tehty ja mietitty ja pohdittu ja paranneltu asioita, ja optimoitu ajankäyttöä ynnä muuta, mut mä aattelen että jotenki et jollain tasolla varmaan, se on tätä nykykulttuuria. En tiedä tarviiko näin laajaa ja isoa systeemiä tähän, semmonen luonteeltani aika duunari, et jos on joku solmukohta, vaikka kahden kesken esimiehen kanssa tai jonkun toisen kanssa ni avataan se ja mietitään nyt se lähdetään tästä päivästä lähtien eri tavoin tekemään. Jotenki, en mä tiedä tarvitaanko niin valtavaa metatason juttua näitten prosessien, mä puhun tavallaan toteuttajatasosta. Mun potilasvirtoihin tää ei todennäköisesti, niissä tehtävään työhön tää ei vaikuta varmaan millään lailla. Tiedonkulkuun vois vaikuttaa, jos miettii, tai vaikka siihen, että mä saan paremmin kun tähän asti ajoissa paperit ja lausunnot kuntoon. Tämmöstä, sen sais ilman tätä laajaa systeemiä. Täytyis vähän optimoida ajankäyttöä ja resurssointia saada vähän lisää. MUt hyvin vieraaksi tää jää jotenki tällä hetkellä.

Puhuja 2 [00:27:48]: Mä ajattelen sitä, et työvälineetkin sinällään, meijän potilastietojärjestelmä ja kaikki muu siihen liittyvä, jos ne sais toimimaan ja jos meil olis semmonen ihminen täällä, jos sul tulee joku ongelma, et se ihminen on siinä saman tien hoitamassa sitä asiaa ja sä voit jatkaa työntekoo, et ei tarvii soittaa moneen eri paikkaan, ootella vastauksia tai sitten se korjataan se vika joskus kahen päivän päästä, sieltä et sä pääset koneella tekemään jotain, se on tehty asioita tosi monimutkaiseksi. Mun mielestä mä en tiedä kuuluuko oikeesti sitten lääkärin tai hoitajan ratkaista tietokoneongelmia koko ajan, mä ite koen et mul olis muutaki tekemistä mitä olis talolle paljon enemmän hyötyä, kun se, että mä tuntitolkulla jonotan johonki, yritän saada apua, kun mikään ei toimi. Se on ollu tässä siitä lähtien kun lähti meidän omat tietokonemiehet.

Puhuja 4 [00:29:14]: Oon kyllä pitkälti samaa mieltä, että jos pitää joku tän hetken solmukohta etsiä niin löytyy kyllä tältä puolelta, et eihän mikään tietokonesysteemi oo aukoton tai täysin toimiva, tässäkin on vähän kommunikaation puutetta. Mä oon monta kertaa atk-ihmisille sanonu, että jos täs ois näin tää toiminto tehty ni se ois helpompaa, mut ei se tunnu menevän kun niitä updatetaan välillä isollakin rahalla, ei tunnu menevän viesti perille. Tai ainakaan niitä ei oteta huomioon. Niitä ei toteuteta. Viime viikolla nyt kun tuli mieleen, oli muutamalla kuntoutettavalla, ei vaan kahdella vaan kolmella viikon kuntoutujaporukasta oli siinä oletusarvona itsemaksava, kun avaa ja menee hoitopolun kautta, siihen tulee itsemaksava. Kun se kuitenkin menee kuntoutusmaksusta, jonka lähettäjä tai maksajataho maksaa viikosta täällä, se on tavallaan euroton se kirjaus, mutta siin täytyy olla valpaana, varmaan on pitkin matkaa tullu tämmösiä virheitä että jotain on jääny laskuttamatta tai, sä oot tarkka siinä, toisin päin varmaan että on vahingossa ollu itsemaksava tai maksusitoumuksella tulleen tiedot siinä vaikka on kuntoutuja auki. Se on yks pieni detalji, mut se kertoo siitä että se on aika raaka ajanvaraussysteemi. Se ei aina hyvin toimi. Joskun on ollut mahdollista laittaa, vaikka on polun kautta otettu, ei oo klikata sitä

istuntoa. Ei oo vaan onnistunu ja sit Elsi Torppa on sen ratkassu, jotaki kiertoteitse. Sin on menny ainakin pari kolme kertaa 20 minuuttia kahdelta työntekijältä.

Puhuja 2 [00:31:24]: Plus sitten, että mä laitan laskua eteenpäin niin siinä mä sitten taas teen seuraavan prosessin sieltä ja joutuu hakeen ja tekeen ja sitä ja tätä et se ei oo yksinkertanen, siin on aika paljon semmosta parantamisen varaa vaikka.

Puhuja 4 [00:31:45]: Mä veikkaan et se ei oo rakettitieteestä kiinni, jos viesti kulkee ja ois niinkun sä sanoit, sisällä joku joka aidosti pystyy sitä hallinnoimaan, tää saataisiin hiottua parempaan kuntoon. Se on semmonen joka vähän ärsyttää tässä pitkin matkaa. Ei kauheesti mutta vähän.

Puhuja 5 [00:32:13]: Me ollaan ratkaisukeskeisen lyhytterapian kannattajia tässä mieluummin kuin pitkän analyysin.

Puhuja 4 [00:32:19]: Joskus se pitkä kohdennettu pieni tönäisy oikeasta kohtaa se on se juttu.

Puhuja 5 [00:32:28]: Puhuin vahingossa Sannan päälle.

Puhuja 3 [00:32:32]: Tuli mieleen uusi järjestelmä, se on toiminut ihan hyvin, taas jos jotain tarvii korjata, esimerkiksi se lääkekortti, aika monimutkaisesti monen kautta pitäis mennä jos laittaa jotain lääkelistalle. Viesti on mennyt eteenpäin mutta vastaus usein on, että se on semmonen järjestelmä, ei sitä pysty enää muuttamaan. Se on vaan semmonen. Nyt kun oli Apottikoulutus, netin kautta. Sitä kuitenkin joutuu käyttämään.

Puhuja 1 [00:33:32]: Haluutko (…) vielä jatkaa?

Puhuja 3 [00:33:34]: Ei, ei.

Puhuja 1 [00:33:36]: Jäiks sulla (…) äsken kesken?

Puhuja 5 [00:33:39]: Ei oikeestaan.

Puhuja 1 [00:33:40]: No mitä te aattelette, näihin leikkauksiin liittyviin tekijöihin, esimerkiksi leikkausaikoihin, tai potilasturvallisuuteen tai potilaan hoitoon leikkauksissa, oisko tällä laatujärjestelmällä leikkauksiin liittyviin tekijöihin vaikutusta? Esim. leikkausaikoihin tai kuinka kauan potilaat on leikkaussalissa tai?

Puhuja 5 [00:34:04]: Ei oo. Se siinä leikkausturvallisuus on se millä oli vaikutusta, oli check-listan käyttöönotto tässä useampi vuosi sitten ja siellä viimesen 30 vuoden ajan on tehty erilaisia mietintöjä siitä, miten vaihtoja nopeutetaan ja miten leikkaussalin toimintaa tehostetaan, ja tää järjestelmä ei tuo siihen minkään valtakunnan muutosta. Olen, käytäntö on sen osoittanut että jos maksetaan taloudellinen palkkio siitä että vaihdot on nopeampia, sit se menee nopeammin. Mutta jos ei haluta maksaa, halutaan tehdä se jollain muulla tavalla, piiri pieni pyörii, ei tuu mitään muutosta siihen.

Puhuja 1 [00:35:00]: Entäs potilasturvallisuuteen leikkauksisa, onko siinä muuta kun tsekkauslistoista, mutta?

Puhuja 5 [00:35:07]: En mä sanois että tässä, mun mielestä meidän potilasturvallisuus on niin hyvä, että jos 99 % parannetaan, se on hyvin marginaalinen, ei oleellista muutosta. Ehkä joku yksittäinen kämmi voidaan torjua, mutta se on oikeastaan sitä, miksi mä oon puhuin tossa aikasemmin prosessien läpikäymisestä, niiden kehittämisestä, joka on semmosta työtä mitä pitäis tehdä ihan riippumatta tämmösestä isommasta sateenvarjosertifikaatista.

Puhuja 1 [00:35:52]: Entäs muut?

Puhuja 2 [00:35:59]: Ei oo kokemusta.

Puhuja 4 [00:36:01]: Se on ihan mun kokemuskenttäni ulkopuolella, mut näin mä olen ymmärtänyt, että kaikki tehdään by the book, ja tarkkoja ollaan niinku pitää olla muutenkin, ilman tämmöstä sertifikaattia.

Puhuja 1 [00:36:12]: Mitä muita vaikutuksia tämmösellä laatujärjestelmän käyttöönotolla vois olla potilaan hoitoon suoraan, potilaan ja oitajan kohtaamiseen liittyen? Onko jotain prosesseissa tai jossain mihin teijän mielestä tämmönen laatujärjestelmä vois puuttua, vai voiko se puuttua?

Puhuja 3 [00:36:34]: Vois vaikka heräämön ja vuodeosaston väliset.

Puhuja 1 [00:36:37]: Mikä siinä on?

Puhuja 3 [00:36:40]: Se on no, esimerkkinä vaikka leikkausten jälkeinen, että tietty ryhmä potilaita sovittu, pidetään alussa heräämön puolella vaikka kaksi tuntia, ja sitten joskus käy näin, että potilaat tulevat aikaisemmin vuodeosastolle ja verenpaineet joskus romahaa ja sitten siinä. Enemmän työtä. Potilasturvallisuus kärsii, mutta ei kovin usein. Yhteistyötä ja tietokulku heräämön ja vuodeosaston kautta. Vois parantaa.

Puhuja 1 [00:37:45]: Mites nyt kun on paljon lehdissä tietosuoja-asioista ja tämmösistä, ajatteletteko että laatujärjestelmä vois semmosiin tietosuoja-asioihin vaikutusta? Vai ajatteletteko et ne on täällä jo sillä tasolla et sitä ei tarvii enää parantaa, tai mitään ei oo tehtävissä?

Puhuja 2 [00:38:06]: Varmasti on parantamisen varaa, koen sen sillai. Meillähän on varmaan joku tietosuojavaltuutettu tai joku semmonen, mun mielestä talossa, mutta en oo ihan varma kuka on, en tiedä tietääks muut? Ei.

Puhuja 5 [00:38:31]: Kyllä meillä minusta (…)

Puhuja 2 [00:38:32]: Onks se (…)?

Puhuja 5 [00:38:33]: (…) vois olla meillä sellanen. Millä tasolla meijän tietoturva on, mulla ei oo faktista käsitystä siitä ja se on varmaan semmonen jota tässäkin talossa mietitään, siinä mielessä varmaan, tämmösissä asioissa vois olla merkitystä sillä. Se on nyt nähtym iten tärkeestä asiasta puhutaan.

Puhuja 4 [00:39:05]: Saattaa olla, että tietoturvakysymys järjestetään kuntoon jos on jotain aukkoa, kaikissa firmoissa (…) tapauksen jälkeen. Voi olla että tää laatusertifikaatti ei edes ehdi siihen, et asiat on riittävän hyvässä jamassa. Ainakin noi (…) on semmonen juttu, että ulkopuolelta siihen ei pääse paitsi jollain ihan erityisillä tekniikoilla. Mul on privaattipuolella, niin sattumalta niilläkin on (…), siellä missä mä pidän privaattivastaanottoa, mul on niiltä yhteys kotoa. Siis pitää varmentaa tietysti ja pitää salasanat ja käyttötunnukset olla ja vaihtuu aika nopeesti myös mutta siihen pääsee mutta (…) ei millään. Semmonen vähän tiukempi portinvartiointi on.

Puhuja 3 [00:40:08]: Jos lähetän sähköpostia ja potilastietoja se kulkee sen henkilönumeron kautta, sille käytetä mitään henkilötunnuksia tai mitään.

Puhuja 2 [00:40:23]: Niin talon sisällä käytetään ihmisen ID:tä ei puhuta nimillä eikä syntymäajoilla vaan annetaan aina se ID. Se on ihan tämmönen talon sisäinen käytäntö ollu jo pitkään. Nythän salaisen sähköpostin kautta laitetaan talon ulkopuolelle lähetettä, jos potilas haluaa, vaikka teksin, et voidaan lähettää niitäkin, mut sit nää järjestelmät ja nää ohjelmat mitä meillä on käytössä, ne on aika monimutkaisia käyttää ja se aikavievää, sanotaan näin, ennen kun sä avaat sen jotain, sä menet ihan eri ohjelmaan ja sul on taas mihin sä kirjaudut ja näin edelleen ja näin edelleen ja saat varmistukset siitä, aika monimutkaiseks on välillä tehty nää asiat kyllä. Sais paljon helpommin.

Puhuja 3 [00:41:31]: Hyvä et potilaalta pyydetään niillä lupalapuilla et ne allekirjottaa, ennen kun pyydetään mitään. Jostain talon ulkopuolelta tai.

Puhuja 2 [00:41:45]: Joo, pitää olla aina lupalaput.

Puhuja 1 [00:41:48]: Onks jotain muuta mitä mä en oo hoksannu kysyä tähän laatujärjestelmän käyttöönottoon liittyen mitä pitäis huomioida? Tai minkälaisia ajatuksia teillä on ollu?

Puhuja 2 [00:42:10]: Mul tulee mieleen semmoset vielä, et meidän työolosuhteet tavallaan, työpisteet, me ollaan ortolla, missä hoidetaan selkää, on hoidettu pitkään, vuosikymmeniä, me omat työpisteet on semmoset, et me ei saada, siin ei oo minkäänlaista ergonomiaa, siin ei oo yhtään mitään, me ollaan aika pitkälti kaikki selkävaivasia ja lonkkavaivasia ja käsivaivasia kun ja siitä ollaan puhuttu ja pyydetty pitkään, että siihen tulis muutos mutta mitään muutosta ei oo tullu, aina vetotaan siihen, ettei oo resursseja siihen. Aika kurjat oltavat, kun me ollaan niin eri mittasia kaikki ja eri kokosia ja samassa pisteessä kun oot. Onneks on hyvät fysioterapeutit talossa.

Puhuja 5 [00:43:18]: Jännää huomata, et eniten vois ajatella, että siitä systeemistä olis hyötyä Helenin työssä, siis asiakirjahallinnointi ja päätösten teko ja tietyllä lailla hallinto, siihen liittyvät hommat ja toisaalta taas eri yksikoiden välinen kommunikaatio. Niissä on selvästi parannettavaa. Ja sitten taas jos ajattelee kahta kredatööriä, vanhat konkarit, me ollaan sitten ehkä, ainakin minä niin omiin tapoihini urautunut, että mä en nää että mulla ois enää mitään toivoa. Tässä suhteessa, tai jotenkin näihin tän tyyppisiin asioihin tuntuu,

että siinä se muutospaine olisi, ei niinkään ehkä potilastyössä. Potilaan kanssa kommunikaatiossa ja varsinaisessa hoidossa. Mut se meijän talon ongelma on ehkä se, että kaikki on keskittyny leikkaussaliin. Leikkausosastoon. Se on sen osasto, jonka toimintaa on aina mietitty, ja vuodeosasto on ollu valitettavasti on ollu vähän sellanen lapsipuoli, sen resurssointi ja asioiden ajattelu vuodeosaston kannalta, siin on kyllä parannettavaa. Kyl näitä kehityskohteita löytyy, tietysti kun istuu ja miettii.

Puhuja 3 [00:45:08]: Mitä luin potilaspalautuslomakkeita, sieltä käy ilmi että hoitotyö ei kaipaa parannusta, mutta just nää tilat ja ruokailu. Niihin.

Puhuja 5 [00:45:25]: Sitten mä luulet että myös se että vuodeosastolla joutuu aika paljon ongelmanratkaisuun ihan yksin. Et se monessa asiassa varmaan olis hyötyä jos meil olis selkeämmin määritelty miten toimitaan. Hoitopolun määrittely vuodeosastolle vois auttaa asiaa. Ei se kaikkeen oo ratkaisu, mutta se on ehkä paremmin toteutettu leikkaussalissa kuin vuodeosastolla, tällä hetkellä.

Puhuja 1 [00:46:17]: Mitä kattoo erilaisia jäjestelmiä, JCI:tä, monethan niistä nimenomaan puuttuu siihen hallintoon, mun mielestä oli tosi hyvä huomio se et ne monesti on tämmösiä hallintoon liittyviä, asiakirjahallintoon tai tietosuojahallintoon tai jotenkin semmosiin liittyviä asiotia, niinhän kun kattoo käsikirjaakin niin siellä on, ja vaikka siellä onkin niitä eri hoitoonkin liittyviä aspekteja, siellä on paljon sitä paperin, mikä liittyy ihan siihen että talo pyörii.

Puhuja 5 [00:46:49]: Kun tosta puhuttiin, onhan näistä laatujärjestelmistä meilläkin puhuttu, mut oliks meillä mitään ISO-järjestelmää missään vaiheessa? Mä en tunne sitä historiaa niin paljon että osaisin siitä sanoo, mut onhan tätä [?? 00:47:05] tehty nimenomaan tehty laatumielessä. Tekonivelpotilaan polkua, ei tää nyt silleen ensimmäinen yritys tässä saralla oo, mut tää on niin laaja ja kaikenkattava, että näin isoo ei kyllä koskaan oo.

Puhuja 4 [00:47:27]: Olihan miellä joskus, oisko siitä jo toistakymmentä vuotta nyt, kun oli tämmönen laajempi oli konsulttikin täällä käymässä ja sillon olin esimiesasemassa niin palaveerattiin siellä täällä aina sillon tällön ja mietittiin mitä parannellaan kommunikaatiota ja työturvallisuutta ja sekin oli aika laaja juttu ja siihen pistettiin rahaakin, sillon oli rahaa tällä firmalla, pistettiin aika paljon rahaa, mutta sitten jotenkin jossain vaiheessa kävi niin, että sitä konsulttia ei enää näkyny eikä kukaan oo siitä systeemistä mitään sanonu. Ja sitten oli semmonen palautejuttu, semmonen takuu oli tavallaan, et jos oli jotain probleemaa, oli tietyt vastuuihmiset, jolle laitettiin viestiä, ja sitten oli se takuu, että vastataan nopeesti, reagoidaan nopeesti. Ei taida olla semmosta postilaatikkoa olemassa. Mutta sekin oli tätä laaduntarkkailun parantamisen yritystä. Irralliseksi jäi se prosessi jotenkin.

Puhuja 5 [00:48:38]: Se on varmaan tämmösiä sporadisia yrityksiä tässä on ollu matkan varrella, ne on yleensä päättynyt aika lailla tähän tyylliin. Se on ehkä yks asia, et ei jaksa kaikesta heti innostua täysin rinnoin.

Puhuja 4 [00:49:01]: Oisko yhteisenä tekijänä, en tiedä onko näin mutta houkuttelevaa miettiä näin, että nää kaikki on tullut ulkoa päin, tarjottu ulkoa, ei oo lähteny perustarpeista tai että työntekijät sais miettiä minkälaista apua tähän nyt tarvittas. Se lähtis tavallaan kasvamaan siitä kasvualustatasolta se ajatus, et on joku ajatus johdolla tai johtoryhmällä tai kellä tahansa, ni (…) on ainakin ollu aina mukana, siis tää Nurmi, näissä jutuissa mukana, mut en tiedä, hänkin on varmaan ollut vähän ulkopuolinen, ettei ollu ihan virroissa mukana. Varmaan se on yhteistä sitten ja nyt tää tulee tälleen ainakin mulle vielä hyvin vieraana ja niinkin kaukaa haettu kun USA:sta tätä. Jotenkin vieraaksi on jäänyt toistaiseksi.

Puhuja 1 [00:50:01]: Elikkä teijän ratkaisu ois se, että mieluummin paikallisia kehittämistoimenpiteitä ja sitä vai kommunikaatiota, hallintoo paikallisesti kehitettäs mutta ei tämmöstä laajaa kattavaa, laatujärjestelmää? Puututtas niihin kohtiin, mitkä tarvii.

Puhuja 4 [00:50:21]: Voihan laajaan puuttua myös, eihän se poissulje sitä mutta mä aattelin et se sisääntulovinkkeli on vähän erilainen, jos se tulee ylhäältäpäin, joku siellä johdossa miettii et nyt täytyy tämmönen ottaa käyttöön, ni sit tavallaan ainahan me (…) ollaan joustettu ja adoptoitu moneen eri suunnanvaihtoon, ja kaikenlaiseen, mutta se ei oikeen kanna pitkää hedelmää, jos se ei tuu omista tarpeista. Kun mä ajattelen omaa potilasturvallisuutta, en usko, ja mä oon itse asiassa aika varma, että tää ei tuo siihen mitään lisäansiota.

Puhuja 5 [00:51:00]: Täs on se että nää oman työhön liittyvät käytännönongelmat on varmaan just tälleen ratkastavissa parhaiten ja tehokkaimmin, mut sitten taas jos me keskitytään vaan siihen, se semmonen hallinnon laadun parantaminen ja tämmöset organisaatiotason tehtävät, ne ei sitten tapahdu. Siinä mielessä voi olla että tarvittas joku systemaattisempi tarkastelu. Mut sillon taas meidän ottaminen siihen tekemään sitä, se on vähän hölmöö hommaa. Sillon täytys olla itsellä joku paljo ja motivaatio tehdä sitä sitten. Mä olen saanut nimityksen lääketyöryhmään, ja alunperin siinä oli semmonen lähestyminen että kysyttiin että voisiksä olla apuna siinä, että vähän kattosit minkälaisia kirjallisia dokumentteja tehdään ja näin poispäin, ni sanoin että sopiihan se, voin mä siinä olla, ei se, mä voin kommentoida lääkkeiden kliinisiä, miten antibiootti, probylaxia, trombiprobylaxia, ja sit sovittiin et mulla on tämmönen rooli ja sit kolmen päivän päästä mä törmäsin siihen yks ryhmäläinen kysy että millon sä kutsut meidät kokoon, kun sä oot puheenjohtaja? Nyt tää meni nyt sitten vähän eri tavalla kun mitä mä, sen jälkeen mä en oo tehnytkään mitään

sen asian eteen, odotan että asiat korjaantuu. Ei, se on ihan kuolleena syntynyt ajatus, että lähtee vetämään jotain täysin eri asiaa kun mitä käytännössä tekee, ei siitä tuu mitään.

Puhuja 1 [00:52:52]: Pitää olla intohimo ja selkee kykös omaan työhön.

Puhuja 5 [00:52:55]: Kyllä. Just tämä, jos tehdään vaan omaan työhön lliittyvää, sillon me ei ikinä päästä koko tähän hallintoon ja huipulle asti.

Puhuja 2 [00:53:16]: Niinhän täällä on aina ollu, jostain tulee että sinulle määritellään uusi työtehtävä tai ihan mitä vaan kyselemättä yhtään mitään onko edes koulutusta tai halukkuutta tehdä sellasta asiaa, sitten vaan pakosta rupee tekeääm ja teet ja teet kunnes opit sen asian ja tottakai pakon edessä vähän silleen, ja lopuksi että huomaat et ootkin sen sisällä ja kaikki on kaatunu kun päälle ja hoidatkin koko putiikkia suurin piirtein ja mitä. Miksi. Miks mä oon suostunu tämmöseen.

Puhuja 5 [00:53:58]: Good luck.

Puhuja 1 [00:54:03]: Meil alkaa aika loppua. Kiitos teille kaikille, tää oli mielenkiintoista ha hyödyllistä tähän tutkimukseen liittyen jos jollakin on lopuksi lisättävää, saa vielä lisätä mutta muuten me ruvetaan lopettelemaan. Hienoa kun olitte mukana.

Useita puhujia [00:54:22]: Kiitos.

[recording ends]

Name of recording: Haastattelu3.mp3 2020

Length of recording: 00:50:03

Information:

[?] = Word was not understood completely but meaning is almost correct. Recording point is written to the text e.g. [word? 00:15:44]

[??] = Word could not be understood and therefore could not be written. Recording point is written to the text e.g. [?? 00:15:44]

[text] = Sounds or not transcribed parts are written with square brackets e.g. [laughs] or [interview paused due to a phone call]

[recording starts]

Puhuja 1 [00:00:03]: Hei minun tarkoituksena on kysellä tässä keskustelussa tästä (…) tulevasta laatujärjestelmästä JCI:stä ja siitä, että minkälaisia odotuksia ja ajatuksia teillä on siihen liittyen, mitä hyötyjä ja haittoja te näätte siihen liittyen, onko siläl teidän mielestä jotain tekemistä esim. potilasturvallisuuden tai työturvallisuuden tai työmotivaation kanssa tai leikkaussaliin liittyviin asioihin. NÄmä haastattelut ovat luottamuksellisia, mutta nyt kysyn luvan, kun nämä nauhoitetaan, kirjoitan lyhyet muistiinpanot ihan vaan siltä varalta, jos tuossa nauhoituksessa tapahtuisi jotakin, niin on jotain kättä pidempää vielä jatkossakin. [?? 00:00:46] mutta myös tämän (…) laatujärjestelmän kehittämiseen, että henkilöt pystyis sitoutumaan siihen tai pystyykö siihen sitoutumaan, että miten sitä pitäisi muokata niin, että henkilöstö voisi sitoutua paremmin? Nämä on täysin vapaaehtoisia, te voitte keskeyttää tämän haastattelun ihan milloin vaan ja nämä anonymisoidaan nämä haastattelut niin, että teitä ei voi yhdistää millään tavalla, että kuka on sanonut mitäkin, voi jutella ihan vapaasti ja tehdään niin, että teitä ei voi yhdistää niihin sanomisiin milläänt avalla, raportoidaan yleisellä tasolla. Aloitetaan siitä, että jos jokainen kertois siitä, että miten te olette osallistuneet tähän laatujärjestelmän käyttöönottoon vai oletteko osallistuneet millään tavalla? Haluatko (…) aloittaa?

Puhuja 2 [00:01:38]: Joo, minä olen yhdessä, kun on näitä työryhmiä jaettu, niin minä olen tämmöinen potilaan arviointi taitaa olla virallinen, niin sitä pohtinut tuossa. Meitä on fysioterapeutti, 2 sairaanhoitajaa ja minä.

Puhuja 1 [00:02:09]: Hyvä. Entäs [?? 00:02:11]

Puhuja 3 [00:02:12]: Mähän en oo nyt ollut vielä missään mukana, että tämä on ensimmäinen tätä uutta laatua, mutta sitten meillä tehtiin vastaavaa kansiota 2000-luvun alussa ja sitten heitin jo kansion roskiin, kun tuli [?? 00:02:31] ja rupes vaikuttamaan siltä,

että raha ratkaisee enemmän kuin mikään laatu, niin vähän kiukuspäissään heitin koko kansion menemään.

Puhuja 2 [00:02:42]: 2000-luvun alussa jo ekat.

Puhuja 3 [00:02:44]: Meillä oli oikein laatukansio siinä. Se oli hieno, iso paksu kansio täynnä tavaraa ja jokainen oli purettu auki ja tätä joka kerta meillä tehdään ja tarkastellaan.

Puhuja 2 [00:02:56]: Mutta se ei ollut tämä JCI?

Puhuja 3 [00:02:57]: Ei, mä en enää muista, että mikä siellä olisi ollut se pohja, millä me lähdettiin, mutta silloin käytiin sitten, kuntoutuksen puolelta varsinkin, niin olin itse mukana tekemässä.

Puhuja 1 [00:03:14]: Oliks se muuten saman tyyppisesti, kun mitä nyt tehdään?

Puhuja 3 [00:03:19]: Mä epäilen, että samalla tavalla. Puren vain paloiksi ja etsitään niitä kohtia, mihin voisi puuttua.

Puhuja 1 [00:03:27]: Entäs (…)?

Puhuja 4 [00:03:27]: Mulla ei ole mitään kokemusta tuosta JCI:stä, olen vain tuolla leikkausosastolla siivoojana ja itte aina, kun tuntuu, että menee väärin, niin keskustelen esimiehen kanssa ja muutetaan niitä systeemeitä, mikä [?? 00:03:49] .

Puhuja 1 [00:03:57]: Miten (…), kun sinä olet ollu siinä työhyvinvoinnissa mukana, niin miten sinä olet kokenut sen, onko sulla koulutus- tai muuta orientaatiota JCI:hin, onko se minkälaista ollut?

Puhuja 2 [00:04:08]: Me saatiin koulutusta meille etukäteen. (…) lääketieteellinen johtaja ja meidän HR-henkilömme (…) nykyään, niin he piti tämmöisen priiffin, tunnin mittaisen, kävi vähän läpi, että mistä tässä on kyse kokonaisuudessaan. Semmoisen olen käynyt läpi.

Puhuja 1 [00:04:42]: Oliko siitä hyötyä siihen teidän työryhmään?

Puhuja 2 [00:04:46]: Se ei mennyt spesifisti siihen, se oli laaja-alainen, mutta siitä sai vähän käsityksen, että mikä se pointti on kokonaisuudessaan. Tämä on yksi palanen tämä meidän homma.

Puhuja 1 [00:05:03]: Jos mietitään laatujärjestelmä JCI:tä, niin minkälaisia odotuksia teillä on siihen liittyen, mitä te ajattelette, että siitä voisi olla, mitä te odotatte vai odotatteko mitään?

Puhuja 3 [00:05:18]: No minä odotan, että se selkiyttää käytäntöjä ja paljastaa, mitkä on heikoimpia lenkkejä.

Puhuja 1 [00:05:34]: Entäs Mervi?

Puhuja 4 [00:05:36]: No ehkä, jos tulee semmosta uutta näkökulmaa, että totakaan ei tullut ajateltua.

Puhuja 1 [00:05:44]: Mitäs (…)?

Puhuja 2 [00:05:48]: Tämä oli varmaan kysymys, että mitä ajatuksia herättää tämä projekti, niin en ole ennen ollut tässä mukana, mutta kuulostaa varsin laajalta, että paljon tehdään töitä sen eteen ja sitten vähän semmoisia, ehkä semmoista vähän skeptisyyttä, että miten se käytännössä tulee käyttöön koko talossa ja sitten, kun vielä (…) ei ole enää me täällä tässä rakennuksessa, vaan se on kaikkia erikoisaloja ja tuolla ympäriinsä, niin se, että miten ne saadaan niputettua yhteen. Kuitenkin ilmeisesti tämä on (…) juttu, että koko (…) koskee, että se olisi jotenkin hallittavissa, jos olis neljän talon, mutta sekään ei sinänsä, koska tämä työ on niin erilaista jokaisessa paikassa, niin en vielä nää semmosia selkeitä linjoja, että näin me tehdään koko talossa. JOku muu osio voilla, mutta meillä se potilaan arviointi on ihan erilaista, mihin tarkoitukseen se arviointi tehdään. Positiivisena ehkä se, että tulee sitten käsiteltyä näitä asioita, vaikka ei saataisikaan mitään yhteistä linjaa, niin ainakin pieni pala joihinkin toimintoihin voisi herättää ajatuksia ja kehittää sitä toimintaa.

Puhuja 1 [00:07:38]: Entäs (…)?

Puhuja 3[00:07:41]: Vähän samoilla linjoilla Tuomaksen kanssa. Uskon kanssa, että semmoisia linjakohtaisia alueita saadaan käsiteltyä ja niihin jonkunlaista yhteistä toimintamallia, mutta että ne toimisi ihan laaja-alaisesti, koska materiaalikin on niin erilaista eri paikoissa. Ehkä ei kaikissa voida mennä samalla kaavallakaan, vaikka olisi kuinka tehty yhtenäistä linjaa.

Puhuja 1 [00:08:19]: Mitä (…) ajattelet?

Puhuja 4 [00:08:19]: Tuosta tuli mieleen, että nyt kun ollaan tässä (…) kanssa yhteistyössä, niin ainakin osa niistä tulee suoraan, että voisi kopioida, että mitä (…) tekee. Kyllä sitten, kun on pienempi paikka ja erilaisia kaikkia, niin pitää sitten viivata erikeen kaikki.

Puhuja 2 [00:08:37]: Jos olen oikeassa, niin eikö (…) ole kesken tämä homma, ne on paljon tämän parissa, mutta nekään ei ole valmiita.

Puhuja 3 [00:08:44]: Joo, mutta ne on paljon pidemmällä.

Puhuja 2 [00:08:45]: Ei voi vielä [?? 00:08:46]

Puhuja 1 [00:08:46]: Ei vielä voi.

Puhuja 4 [00:08:50]: Minä oon sen verran laiska, että valmis malli.

Puhuja 2 [00:08:54]: Onhan se totta, että jos tulee päällekkäisiä ja varmasti tuleekin.

Puhuja 3 [00:09:03]: Jos minä ajattelen näitä kipuasiakkaiden kanssa toimintamallia, niin ainakin näin, kun tietää (…) toimintamallin ja meidän toimintamallin, niin ne saa jotenkin samaan muottiin. Melkein tekis mieli sanoa, että toivon, ettei panna samaan muottiin.

Puhuja 1 [00:09:38]: No mitä te ajttelette erityisesti, jos puhutaan noista hyödyistä tai onko jotakin haittaa tämmöisen laatujärjestelmän käyttöönotossa?

Puhuja 2 [00:09:48]: No ainakin, jos asioista mennään pilkulleen, mitä on kirjattu, niin ei tämä toimi, jos tekis ihan kaiken sillä tavalla, mitä on kirjattu siihen, että miten me toimimme ja miten nämä auditoidaan, niin eihän tätä voi pilkulleen noudattaa, että ehkä vähän tuntuu tietenkin, että voi tulla ja toivottavasti siitä ei tule liian jäykkää.

Puhuja 5 [00:10:31]: Pahoittelut, pääseekö vielä osallistumaan, tulen näin kesken?

Puhuja 1 [00:10:31]: Pääsee, hyvä kun tulit. Sinä olet varmaan (…). Oliko sulla mulle joku lomake?

Puhuja 5 [00:10:36]: No eipä mulla ole.

Puhuja 1 [00:10:38]: No ei se mitään, mutta kysyn, että saako sinun puolesta nauhoittaa?

Puhuja 5 [00:10:41]: Saa, niin se oli se lomake, juu kyllä saa.

Puhuja 1 [00:10:45]: Tiedäthän, että tämä on vapaaehtoista ja tämä anonymisoidaan ja tän voi keskeyttää milloin tahansa ja minä olen (…) ja olen tutkijana tässä hankkeessa. Tarkoituksena kysyä tästä laatujärjestelmän käyttöönotosta ja me oikeastaan jo aloiteltiinkin.

Puhuja 5 [00:10:59]: Joo, meillä oli aika hasardi tilanne osastolla.

Puhuja 2 [00:11:04]: Joo, sitä vaan, että onko siinä semmoinen riski, että tulee tämmöistä kauheen jäykkää toimintaa ja semmosta ylimääräistä, mitä ei välttämättä juuri haluaisi juuri tältä potilaalta käyttää aikaa siihen, en tiedä.

Puhuja 5 [00:11:29]: Kun sinä (…) vedit tätä laatujärjestelmää, niin minkälaisia odotuksia sulla on? Näätkö jotain hyötyjä ja haittoja sen käyttöönotosta?

Puhuja 5 [00:11:39]: No itse asiassa minun tietämys itse laatujärjestelmästä on aika vähäinen. Ei siitä minun mielestä ole ihan kauheesti tiedotettu, osastolla ainakaan. Ei me päästä koskaan näihin auditorion aamumeetengeihin tai muuallekaan, että siellähän yleensä näist ätiedotetaan. Se, mitä mulla on tiedossa, niin voisi olettaa, että varmaan on aika paljon

hyötyjä just esim. työtapojen yhdenmukaistamisessa, kun niissä on aika paljon eroja, että tulisi semmosta selkeyttä ja johdonmukaisuutta ja sitä kautta myöskin turvallisuutta.

Puhuja 1 [00:12:32]: No näätkö sä mitään haittaa tämmösen käyttöönotossa tai mitä haittaa siinä voisi olla?

Puhuja 5 [00:12:38]: Ei mulla nyt ihan äkkiseltään tule.

Puhuja 1 [00:12:41]: Oisko (…), ajatteletko, että vois olla jotain haittaa tämmösessä laatujärjestelmän käyttöönotossa?

Puhuja 4 [00:12:46]: En tiedä, ehkä jos siitä tulee semmosta mekaanista toimintaa, että ei se lähikontaktit kärsi silleen siinä potilaan kanssa.

Puhuja 1 [00:13:02]: Entäs (…)?

Puhuja 3 [00:13:06]: No minä toivon tietenkin kanssa, että se ei vie itse potilastyöstä aikaa, että ei tule semmosia kirjallisia lippulappuja, mitä pitää alkaa täyttämään, jotka monta kertaa näissä laatujutuissa kysellään kauheesti, mutta sitten esim. ei välity meille tekevälle tasolle mitään palautetta siitä, että me on uhrattu aikaa ja täytelty kaiken maailman kyselyjä ja vastauksia ja tehty jotain analyysiä, niin meille ei kerrota..

Puhuja 4 [00:13:41]: Ei tule mitään tulosta..

Puhuja 3 [00:13:43]: Sen toivoo aina, että mitä tahansa tämmöisiä tehdään, niin niistä tulisi palaute ihan sinne alatasolle saakka, että mitä tämä tuotti, antoiko mitään lisäarvoa.

Puhuja 2 [00:13:57]: Olis kiva, jos oikeesti tärkeitä asioita, mitä itsekin kysyis, niin ettei tulis päällekkäisyyttä, että sitten voisi katsoa jonkun laatikon, että nämä ja nämä asiat on jo kysytty. Semmosia, että ne oikeasti hyödyttäis meitä kaikkia. Joku semmoinen yhteinen nimittäjä, että saataisiin myös kliiniseen käyttöön, ettei se uppoaisi jonnekin tutkimusaineistoon. [?? 00:14:38]

Puhuja 3 [00:14:40]: On tehty paljon kaikenlaisia tutkimuksia ja seurantoja, joista sitten palaute jää meille ihan mysteeriksi.

Puhuja 1 [00:14:48]: Oletteko te sanoneet sitä eteen päin, että..

Puhuja 3 [00:14:50]: On annettu palautettakin. Minä olen ollut täällä vuodesta (…), niin on ehtinyt olla monenlaisissa projekteissa mukana. Aina sanon jo heti nykyään osallistuessani, että toivon myös saavani palautteen tässä, kun kuitenkin olen palvellut taloa.

Puhuja 1 [00:15:05]: Kyllä. Täytyykin sanoa tästä (…), että kun nämä palautteet tehdään, niin ne tulee myös kaikille tietoon. Minkälaisia tuloksia tämmöisestä laatujärjestelmän käyttöönotosta voisi olla tulevaisuudessa?

Puhuja 3 [00:15:34]: Sillä pitkällä kokemuksella voin sanoa, että yleensä aina on ollut jotain hyötyä, mitä on käytännön työssä nähny, että joku käytänne, jos se on ollut ihan [?? 00:15:47] , että jokainen on soveltanu omaa mallia, niin siihen on saatu jotain käytännön yhteistä linjaa.

Puhuja 5 [00:15:59]: Kyllähän se varmaan lisää tehokkuutta.

Puhuja 1 [00:16:06]: Mites se tehokkuus lisääntyy, jos ajattelet ihan konkreettisesti arjen työssä?

Puhuja 3 [00:16:14]: Varmaan ainakin niin, että jos tulee joku ongelmatilanne, että kaikki heti tietää, että okei, näin tässä kuuluu menetellä, jos ei mulla ole siihen taitoja tai mahdollisuuksia juuri sillä hetkellä, niin voin kollegalta kysyä, että mites tämä nyt menikään, jos tulee joku ongelmatilanne, niin silloin, kun on sovittu joku yhteinen malli, niin aina työporukasta joku muistaa, että näin me tehdään.

Puhuja 5 [00:16:38]: Se olis sit tärkeetä, että kaikki sitoutuisi siihen, ettei kukaan lähde sooloilemaan, koska eihän siitä ole mitään hyötyä silloin.

Puhuja 3 [00:16:48]: Se on minun mielestä tärkeä asia niistä laatukriteereistä, se antaa meille toimintaan linjat.

Puhuja 1 [00:17:05]: Millaisia muutoksia tämmöinen laatujärjestelmän käyttöönotto vois tuoda teidän arjen työhön ihan konkreettisesti, just kun puhuttiin noista yhteisistä linjauksista ja voisi tuoda lisää tehokkuutta ongelmatilanteissa, mutta onko muita ajatuksia, mitä se teidän arjessa voisi vaikuttaa?

Puhuja 5 [00:17:25]: Minä toivon ainakin, että se helpottais sitä omaa työntekoa, semmonen turha työ jäisi pois.

Puhuja 2 [00:17:36]: Se turha työ, se just, sitä se on parhaimmillaan, mut pahimmillaan se tuottaa turhaa työtä. Juuri jos kirjaillaan kauheesti jotain epäolenaista juuri siinä potilaskontaktitilanteessa.

Puhuja 1 [00:17:59]: Entäs..

Puhuja 4 [00:18:01]: Ehkä just, jos on valmis malli tms. käytäntö, niin se nopeuttaa, ettei tarvii erikseen jäädä pohtimaan.

Puhuja 3 [00:18:13]: Hyvä esimerkki on nyt meillä käytössä oleva Haipro, niin se tuli just sitä kautta, kun todettiin, että meillä ei ole mitään, mihin me kirjattais niitä ongelmatilanteita ja nykyään on sit semmonen käytettävissä, johon tiedetään heti, että ne pystytään kirjaamaan ja sitä kautta ne käsitellään ja asiat menee eteen päin eli se on tämmöinen hätä.. onko se sulle tuttu?

Puhuja 1 [00:18:36]: Mä en tunne.

Puhuja 3 [00:18:38]: SE on semmonen, mihin me kirjataan kaikki, jos meinaa olla läheltä piti -tilanteita tai sattuu joku vahinko, niin se on juuri sen seurausta, kun oli tehty tätä laatusysteemin läpikäyntiä. Silloin se on mielekästä se kirjaaminen, kun tietää, että tämä tuottaa tulosta.

Puhuja 5 [00:19:02]: Siitä on apua silloin, kun ei etsitä syyllisiä.

Puhuja 1 [00:19:06]: Onko sillä Haiprolla semmosta työturvallisuuteen tai potilasturvallisuuteen liittyvää?

Puhuja 3 [00:19:10]: On.

Puhuja 1 [00:19:12]: Vähän sama asia tässä laatujärjestelmässäkin haetaan juuri sitä, että se potilas- ja työturvallisuus olisi korkeammalla tasolla.

Puhuja 3 [00:19:20]: Että osataan jo ennakoida tavallaan niitä tilanteita.

Puhuja 1 [00:19:25]: NO miten te ajattelette tämmösestä laatujärjestelmän käyttöönotosta, että miten se voisi vaikuttaa työturvallisuuteen muuten kuin tuon ennakoinnin kautta? Varmaan yhteiset toimintamallit, mitä tuli jo esille ja ne linjaukset, että jos on jotain ennakoimatonta, niin miten toimia. Mietin vaan, että onko tämä Haipro ja tämä vähän..meneekö..

Puhuja 3 [00:19:59]: Onhan se, että käyttäjät ja ortopedit merkkaa, että kumpi polvi leikataan ja kaikkea tällaisia käytäntöjä, mitä on matkan varrella tullut tämmöisiä toimintamalleja, ettei leikata väärää polvea tms. Nyt heti keksin, että mitä tämän pitäisi tuoda tullessaan.

Puhuja 2 [00:20:23]: Elikkä työturvallisuuteen, jos se sitä kautta vähentäis tahtia tai sähläystä, niin sitten, mutta jos mietin oman vastaanoton kautta, niin mitä, jos pistän injektioneulan ranteeseen tai jotain, niin ei se välttämättä mulle tunnu työturvallisuudessa, mutta osastolla varmaan enemmän.

Puhuja 5 [00:20:54]: Varmaan joo.

Puhuja 1 [00:21:02]: Niin, että jos osastolla, miten se osastolla sitten näkyis?

Puhuja 2 [00:21:10]: Just se, että jos se sähläys, en mä sano, että te sähläätte siellä, mutta just ei tule semmosta virhealttiutta siinä ja jos saatais vähän työtahtiakin alaspäin ja stressiä alas, niin kyllä se sitä kautta muhunkin vaikuttaa toki. Kun tekee toimenpiteitä ja sohasee vahingossa hanskan läpi, kun on stressissä ja käsi tärisee, teoreettista.

Puhuja 5 [00:21:51]: Kyllä se kiire lisää vahinkoja ja riskejä.

Puhuja 1 [00:22:04]: Se on niin sääli, että tämmöinen laatujärjestelmä ei poista sitä kiirettä, vaikka se poistais sitä sählläämistä.

Puhuja 2 [00:22:11]: Juuri, jos jotkut linjat selkeytyis, niin ei tarttis miettiä sitä, että mitä nyt tämän kanssa teen. Jos se säästäisikin aikaa.

Puhuja 1 [00:22:26]: Niinpä.

Puhuja 3 [00:22:28]: (…) on ollut niin pieni yksikkö ja pyritty siihen, että nopeasti reagoidaan muuttuviin tilanteisiin, niin meidän on tarvinnu osaltaan olla tämmöistä laadun miettimistä koko ajan täältä sisältä päin, mutta sitten se osaltaan, kun tehdään niin nopeita muutoksia, niin kyllähän se on tietenkin sähläystä aina hetken aikaa, kun keksitään taas joku uusi juttu, että okei, tehdään tämä nyt näin tai näin, kun tarve onkin tämmöiselle. Sitten välillä on hyvä pysähtyä, että onkö näin hyvä tehdä tätä, kun on näin ollu pitkän aikaa ja polkastu erilaisia juttuja, että tarvitaan tätä ja tätä, mutta ei oo tehty semmoista yhteenvedonomaista hetkeen.

Puhuja 5 [00:23:25]: Kyllä osastollakin, just tämän myötä tulee koko ajan kaikkea uutta kovasti, mitä pitää omaksua lyhyessä ajassa, eikä oikein saa edes perehdytystä niihin asioihin.

Puhuja 3 [00:23:43]: Se mulla olikin mielessä, että perehdytys, siihen ei koskaan voida panostaa liikaa. Jos on asia, johon pitäis, se ei riitä, että joka kerralla annetaan kauhea pläjäys, siitä kuitenkin puolet menee ohi. Sen pitäisi olla sellasta, että siihen voi palata. Sitä perehdytystä vähän kerrattais vielä kuukauden tai parin päästäkin vielä.

Puhuja 5 [00:24:08]: Kun tulee ihan tuntemattomia potilaita leikkaukseen, niin meidän pitäisi hallita ihan älyttömästi asioita sillä, että sieltä tulee kerran sairaanhoitaja Töölöstä kertomaan niistä toimenpiteistä meille osastotunnille, niin kyllä se lisää henkistä kuormittuneisuutta tosi paljon.

Puhuja 1 [00:24:30]: Miten se (…) yhdistyminen näkyy nyt teidän arjessa?

Puhuja 5 [00:24:35]: Muuttuneissa tilanteissa.

Puhuja 1 [00:24:47]: Niin, jos se perehdytys on ollut heikkoa ja jotenkin nopeeta, niin ei siinä paljon. MIten te ajattelette, että tämmöinen laatujärjestelmän käytöönotto voisi vaikuttaa teidän työhyvinvointiin vai onko sillä mitään vaikutusta siihen?

Puhuja 3 [00:25:06]: Kyllähän sitä kautta tulee sitä hyvinvointiakin, kun on selkeet toimintamallit.

Puhuja 4 [00:25:17]: Ehkä se piristää silleen, että taas mietitään uusiksi.

Puhuja 2 [00:25:27]: Oliko siellä yhtenä asiana työhyvinvointi tms? Jos se kehittäis meidän tykytoimintaakin?

Puhuja 5 [00:25:33]: Anteeks, mitä kehittäis?

Puhuja 2 [00:25:38]: Joo joo.

Puhuja 3 [00:25:40]: Meillä on semmonen vitsi, kun on verrattu tähän taloudelliseen tilanteeseen. Meillä on tänä päivänä juuri pitkästä pitkästä aikaa vähän niin kuin työhyvinvoinnin nimissä tämmöinen talo tarjoaa aamupalaa, että se on tämmöinen harvinainen, mitä on tullut, mutta on monet kerrat esitetty toiveita, että olis jotain yhteistä mukavaa puuhaa, joka ei ole työntekoa, vaan jotain vallan muuta.

Puhuja 2 [00:26:14]: Jos teillä ei ole, niin se pitäisi olla, työhyvinvointi.

Puhuja 3 [00:26:19]: Ehdottomasti.

Puhuja 2 [00:26:20]: Pitää soittaa sinne jenkkeihin, että tämmöinen pitäisi sallia [?? 00:26:29] pikimmiten.

Puhuja 1 [00:26:31]: Kokonainen päivä. Miten te ajattelette, että tämmöinen laatujärjestelmän käyttöönotto vaikuttais (…) tulevaisuuteen? Onko se joku kilpailutekijä teidän mielestä talolle vai miten te ajattelette?

Puhuja 4 [00:26:54]: Kai se on markkinoinnissa, hei hei täällä meillä.

Puhuja 3 [00:27:02]: Se ei välttämättä näy meille niinkään, kun me ei olla semmosessa tilanteessa, varmasti ylempi porras, joka näitä neuvotteluita ja sopimuksia tekee, niin varmasti paremmin näkyy, että se yksi valttikortti.

Puhuja 4 [00:27:15]: Meriittiä lisää.

Puhuja 3 [00:27:23]: Hyvä maine ei tänä päivänä pelkästään yksistään riitä.

Puhuja 5 [00:27:32]: Just kuulin, että Terveystalokin alkaa tekemään näitä tekoniveliä.

Puhuja 4 [00:27:36]: Täällä vai?

Puhuja 5 [00:27:36]: Ei, vaan ilmeisesti Ruoholahdessa. Tämä oli tämmöinen sivukeissi, tätä ei tarvii laittaa.

Puhuja 3 [00:27:46]: Ei kun minä olen tässä odotellu, kun maanantaisinhan on (…), mut minä sanon sitä Tarjoustaloksi.

Puhuja 1 [00:27:54]: Voitte tarjota.

Puhuja 3 [00:27:58]: Sillä se varmaan pääsikin.

Puhuja 1 [00:28:02]: Lisääkö ne niitä leikkauksia?

Puhuja 5 [00:28:07]: [?? 00:28:07] niitä leikkauksia.

Puhuja 4 [00:28:19]: Minä ajattelin, että se muuten vaikuttaa, vaikka markkinoinnin kautta (…) tulevaisuuteen tuo järjestelmän käyttöönotto.

Puhuja 2 [00:28:28]: Se on varmaan kokonaisvaltaisesti, mitä ollaan tässä työtehtäviä käyty läpi. [?? 00:28:38] vaikuttaa.

Puhuja 1 [00:28:44]: Miten te ajattelette, että tämmöinen laatujärjestelmän käyttö tulisi vaikuttamaan noihin leikkauksiin liittyviin tekijöihin ja leikkaussalin toimintaan?

Puhuja 2 [00:28:55]: Kyllä varmaan potilasturvallisuus voisi parantua. Leikkausajat ehkä lyhentyis, vaikka luulen, että aika tappiin se on jo laitettu.

Puhuja 5 [00:29:10]: Itse ajat, joo, mutta en tiedä, onko niissä vaihdoissa ja muissa hiomista vielä, en tiedä.

Puhuja 2 [00:29:15]: Siitä Manninen piti just esitelmän, mikä on ollut jo täällä, että on tutkittu niitä vaihtoaikoja jne. Sitä on jo tehty. Voihan sieltä tulla jotain uutta.

Puhuja 4 [00:29:41]: Minä olen miettinyt, kun me tehdään noi vaihdot jne., niin siellä usein voi olla niin, että ihan erityyppinen leikkaus tulee ja siinä menee ylimääräistä aikaa, kun odotetaan eri pöytiä sinne ja tänne, että miksei niitä leikkauksia voida suunnitella niin, että yhtä asiaa voisi tehdä yhdessä asiassa, että selkäpöydät jne. Se on välillä semmosta ruljanssia niiden siirtäminen. Nythän tehtiin viimeksi, oliko se topprojektin tiimoilta se, että on yksi ortopedinen sali, siellä tehdään vain pelkästään ortopedisia, yleensä.

Puhuja 5 [00:30:24]: Toihan olisi ihan hyvä kehittämisidea, silloinhan ne vaihtoajatkin lyhenisi, kun ei tarvitsisi vaihdella niitä kalusteita.

Puhuja 4 [00:30:32]: Ja minä olen käsittänyt niin, en tiedä, kuka niitä leikkauslistoja tekee, että tehdäänkö ne osastolla vai miten, niin ne ei välillä käy meikäläisen järkeen, että mitä järkeä tässä on tässä touhussa, että vaihdellaan edes takas.

Puhuja 3 [00:30:47]: Osasto ja leikkuri tekee yhteistyötä leikkauslistojen suunnittelun suhteen.

Puhuja 5 [00:30:52]: Se on kuule semmosta sillisalaattia, että se ei ole ihan yksinkertaista.

Puhuja 4 [00:30:58]: Mullekin tulee ja heijastuu se, että mitä tässä tapahuu.

Puhuja 1 [00:31:05]: Mitä teidän mielestä, että yhden tyyppiset leikkaukset aina yhdessä salissa jne?

Puhuja 4 [00:31:13]: En tiedä, että onnistuuko se sitten, jos on vähemmän toisia leikkauksia ja toisia enemmän, se on niin alkuvaiheessa sillon, kun sovitaan niitä leikkauspäiviä, että miten sitä pystyy muka järjestämään.

Puhuja 3 [00:31:36]: Siinä on varmaan yksi kehittämisen paikka.

Puhuja 1 [00:31:51]: Sanoit Tuomas, että se voisi vaikuttaa potilasturvallisuuteen siellä leikkaussalissa.

Puhuja 2 [00:31:56]: Niin, kyllä, jos käydään [?? 00:32:02] läpi ja mikä on [?? 00:32:05] , mitä se JCI:kin edellyttää, niin sitä kautta.

Puhuja 1 [00:32:16]: MItä muita vaikutuksia tällaisella laatujärjestelmän käyttöönotolla voisi olla siihen potilaan hoitoon? Näkyykö se potilaan kanssa työskentelyssä? Vaikka sanoitte, että ei tarvii siitä ruveta mitään kirjaamaan...

Puhuja 3 [00:32:39]: Minä ajattelisin, että kyllähän se voi siellä taustalla vaikuttaa, mutta kyllähän se potilas itsessään pitkälti sanelee, kuinka minä hänen tilanteessa etenen.

Puhuja 5 [00:32:49]: Niin, kun ethän sinä voi jokaista hoitaa samalla tavalla.

Puhuja 3 [00:32:49]: En minä sitä laatujärjestelmää näe sillä lailla ohjenuorana siihen asiakkaan kanssa.

Puhuja 5 [00:33:02]: Kyllä se voi helpottaa sun työtä sillain, että voit perustella niitä asioita ehkä.

Puhuja 3 [00:33:08]: Niin, sanotaan että ehkä semmoisessa epäselvässä tilanteessa, kun potilas kyseenalaistaa jotakin, niin voin vedota johonkin laatujärjestelmään.

Puhuja 1 [00:33:17]: Niin, että tämä on kuulkaa tutkittu juttu.

Puhuja 5 [00:33:22]: Ehkä jotain tällaista.

Puhuja 2 [00:33:37]: Sitten tiedon saanti voi muuttua sitä kautta, kun on vähän strukturoitu juttu, mihin joku on saattanut kirjata asioita, niin näkyy selkeesti ja voi nopeasti kattoo, mitä potilaasta on kirjattu. Esim. kivun kuvauksia, MRSA kipupiirroksia jne.

Puhuja 1 [00:34:13]: Onko teillä muita ajatuksia, mitä hyötyjä voisi olla laatujärjestelmän käyttöön otolla? Mitä pitäisi huomioida, mitä en ole hoksannut kysyä tai..?

Puhuja 3 [00:34:32]: Ne on tullut jo esitettyä toiveet. Ja tosiaan se palaute, kun tämä on pitkä prosessi, niin aina välillä informaatiota, missä mennään jne. Silloin on alemmankin tason helpompi ymmärtää, minkä takia pitää vastailla johonkin kysymyksiin tai osallistua johonkin prosessiin omalla pienellä panoksellaan. Sitten se helposti jää, kun en nyt ihan tarkkaan tiedä, mitä kuvioita tässä on, mutta useimmiten se liittyy siihen, että se työryhmä saa tehtäväkseen jotakin ja niiden pitää kysellä kollegoilta, että kuinka te teette tätä ja kartoitetaan sitä ja sitten se vastausprosentti tahtoo jäädä vähän heikonlaiseksi, kun se motivaatio ei ole tekevällä tasolla niin selvää, että miksi me näitä kyselyitä ja prosesseja aina tehdään, mutta kun sieltä tulee aina välillä vähän palautetta, vaikka lyhyttäkin, että nyt tämä etenee näin ja kiitos, nyt olette antaneet tämmöistä palautetta, niin se aina lisää sitä motivaatiota projektin eteen päin viemiseen.

Puhuja 1 [00:35:57]: Tuomas sanoit, että teillä on oma työryhmä sen potilaan arviointiin, niin minkälainen aikataulu teillä on tai oletteko te suunnitelleet steppejä?

Puhuja 2 [00:36:06]: Joo, me ollaan hyvin alkutekijöissään, kartoitus käynnissä, mutta se on aika hyvin tehty jo, että Kristiina on hoitanut osaston käytännöt ja kuntoutuksen ja sitten

taas polikliiniset toiminnat ja nyt sitten Elina on osaston toimintaan selvittänyt ja siltä osin, miten kipua arvioidaan, siinähän on muutama osio. Sitten siihen kuuluu myös rtg ja laboratorio ja ne on omanaan selvityksessä. Sen verran ollaan päästy eteen päin ja siitä mennään pala kerrallaan eteen päin ja sitten meidän pitäisi seuraavaksi pohtia, että onko tässä jotain suuria linjoja, mitä voi vetää, että kaikki nämä toiminnot, mitä tässä luettelin, että onko kivun arviointi, voiko jotain yhtenäistämään, mutta hankalaa tulee varmaan olemaan.

Puhuja 1 [00:37:19]: Miksi se tuntuu hankalalta?

Puhuja 2 [00:37:23]: se toiminta on kuitenkin niin erilaista, että siksi me verrataan noita eri osa-alueita. Kaikilla voi olla jokin numeerinen kivun arviointi, mutta sitten ortopedeille sopii tietynlaiset ja meille sopii tietynlaiset, mutta kyllä niissä varmaan jotain yhdistettävää on.

Puhuja 1 [00:37:53]: Ootteko te puuttunut semmosiin tavallaan laatupoikkeamiin tai puhutteko te semmosista, että jos jossain paikassa ei toimikaan, niin kuin pitäisi.

Puhuja 2 [00:38:03]: Niin, että reagoidaanko me siihen välittömästi?

Puhuja 1 [00:38:06]: Niin.

Puhuja 2 [00:38:04]: Ja viedään käytäntöön?

Puhuja 1 [00:38:08]: Niin.

Puhuja 2 [00:38:08]: Me ollaan niin alkuvaiheessa, että ei ole tullut näitä tilanteita vielä. Se jää nähtäväksi, voin myöhemmin kertoa, että käytiinkö me asian kimppuun heti.

Puhuja 1 [00:38:25]: Entäs osastolla, mitäs sää ajattelet, jos on tämmösiä työryhmiä sielläkin, olisko siellä jotain, mihin voisi heti saman tien tarttua?

Puhuja 5 [00:38:42]: En edes tiedä, että mitä kaikkia työryhmiä on olemassa, että on vähän vaikea tohon sanoa mitään.

Puhuja 3 [00:38:51]: Kyllä meillä talossa on mun mielestä vähän semmoinen perinnekin, että jos on jotain isompaa ongelmaa, niin kyllä siihen pyritään tarttumaan aika nopsasti, ettei välttämättä tarvitse [?? 00:39:02] tms.

Puhuja 5 [00:39:03]: Kyllä, tämä on sen verran pieni yksikkö, niin ei tarvii mitään hirveitä byrokratiaa käydä.

Puhuja 3 [00:39:10]: Jos vaan vie sitä asiaa eteen päin, kun aina välillä on se ongelma se, että jossain tilanteissa on tullut niin, että on päässyt kärjistymään, kun asianosainen ei ole lähtenyt heti viemään eteen päin, jos hänellä joku mättää, niin sitten [?? 00:39:23] ja avautua, meistä kukin käyttäytyy omalla tavallaan.

Puhuja 1 [00:39:28]: Osaatteko te sanoa, että mille henkilöstöryhmälle teistä tämmöisestä laatujärjestelmästä olisi enemmän hyötyä? Mietittekö te, että se olisi just siihen leikkaussalityöhön tai siellä tiettyihin henkilöstöryhmään tai joku hallinto tai maksuliikenne tai mikä se voisi olla?

Puhuja 3 [00:39:54]: Eiköhän se ole enemmän kokonaisvaltaisempaa. Sanotaan, että silloin monta kertaa enemmän, kun me on tehty näitä laatujuttuja matkan varrella, että en tähän osaa sanoa, se on paljastunut paremmin, että mitä kaikkea siihen kokonaisuuteen kuuluu, että sitä ei välttämättä itse hahmotakaan, että mitä kaikkea palasia tässä on, ennen kuin tämä on, että se koko prosessi tulee paremmin näkyväksi, kun ne pannaan siihen paperille ja puretaan ja keskustellaan. Sitten, ehkä johto on ehkä havainnut, että hops heijjaa, täällähän on osaavaa henkilökuntaa. Me on huomattu sitten, että valkenipa niillekin.

Puhuja 5 [00:40:46]: Ja paljon semmosta työtä joka organisaatiossa, mikä ei näy joka paikkaan, vaikka se on todella olennaista kokonaisuuden kannalta.

Puhuja 3 [00:40:48]: He ovat tyytyväisiä, että homma rullaa. Kyllä he ovat ymmärtäneet sitä, että välttämättä edes, kuinka hyvin se rullaa ja ei aina rattaat ole ihan kohdillaan, mutta silti se homma on saatu rullaamaan.

Puhuja 2 [00:41:13]: Minä luulen, että suurin hyöty vois olla tämmöisessä akuutissa toiminnassa, niin kuin osasto- ja leikkaussalitoiminnassa. Sen laadun varmistaminen, mutta

muissa näissä polikliinisissa meillä on enemmän varaa sählätä siellä. Kun jotain tapahtuu, niin...

Puhuja 3 [00:41:34]: Ei ole henki vaarassa.

Puhuja 2 [00:41:34]: Niin. Toki sielläkin hyöytyjä on tai se on ehkä tärkein, että siihen kannattaa panostaa.

Puhuja 5 [00:42:00]: Siellä on ehkä niin monta vaihetta, että jos niitä jokaista vaihdetta sais edes vähän hiottua, niin kyllähän se kokonaisuus sitten vaikuttaa paljonkin.

Puhuja 1 [00:42:18]: Mites Mervi sun työhön, mitä sä ajattelet tähän?

Puhuja 4 [00:42:23]: Eipä varmaan paljon mitään, että mä teen itte niitä päätöksiä, kun meillä on vaan 2 työntekijää vain aamu- ja iltavuorolainen, siinä ei paljoa.

Puhuja 1 [00:42:45]: Onko teillä jotain lisättävää vai ollaanko me valmiita?

Puhuja 2 [00:42:52]: Mites tämä jatkuu tämä tutkimus, oliko tämä tässä?

Puhuja 1 [00:42:58]: Tämä jatkuu niin, että tästä tehdään nyt yhteenvedot ja sitten tehdään raportti ja sitten (…) oli semmoinen ajatus, että jatkettais sitten seuraavassa vaiheessa, kun se on otettu käyttöön se laatujärjestelmä, niin sitten tehtäis kanssa tämmöinen vastaava haastattelu tai kyselyselvitys, että miten se eroaa tästä 0-tutkimustasota, mikä nyt on. Nyt on tämä 0-taso, kun ei olla varsinaisesti vielä otettu, kun nämä työryhmät on vasta lähtenyt käyntiin. Parin vuoden päästä, kun se on otettu käyttöön, niin sitten olis se 3. osio, että onko oikeasti joku muuttunut. Ajatuksena olisi, että siitä raportoitaisi koko henkilöstölle, mitä nämä tulokset on, että oikeasti se käyttöönotto voisi vaikuttaa, että ei vaan kirjoiteta tieteellisiä artikkeleita, vaan enemmän niin, että vaikuttaisi tai kehittäisi myös samalla tätä prosessia, että samalla paranis tai jos sitä pystyy parantamaan jotenkin, se käyttöön otto ja että teidän äänet tulis kuuluviin, että se otettas niin käyttöön, että se myös oikeasti hyödyntäisi teidän työtä.

Puhuja 2 [00:44:07]: Tuostakin tuli mieleen, että jos tehdään jotain artikkeleita, niin ihan semmonenkin yleinen [?? 00:44:13] , että tämmöinen julkaistiin teidän keräämillä tiedoilla, niin sehän voisi olla ihan YES.

Puhuja 1 [00:44:17]: Ja aika paljonhan täällä on eri tutkimustoimintaa, mitä tehdään tämmösestä kehittämistyöstä, että mielenkiintoistahan se on kuulla, että se on tehty omassa talossa.

Puhuja 3 [00:44:30]: Ja minä luulen, kun tässä on ollut noita YTeitä jne aika paljon, niin tämmöset väliportaan esimiehet, niin he ehkä eniten kaipaa tämmösiä ohjenuoria toiminalleen, että tekevä taso tekee sillä rutiinilla ja meillä on työntekijät, aika moni, semmosia pitkän linjan tekijöitä. En tiedä, sairaalan puolella, kun on tullut näitä uusia toimintoja, niin sen mukana uusia tekijöitä, jotka ei välttämättä ole ehkä niille niin tuttuja, mutta sitten meidän väliportaan esimiehet, niin ne on välillä ihan pulassa, kun on niin kauheesti kaikenlaista, niin että miten ne itse kokee ja jaksaa työssään, että toivon, että heitä haastatellaan tässä työssä. Minä luulen, että sieltä voi pongahtaa sellaisia kehittämiskohteita, joihin me ei osata sanoa mitään, mutta me nähdään, että siinä kohtaa on yksi semmoinen solmu, että projektin siitä ylöspäin ja sieltä alaspäin, en tiedä, onko se siinä, mutta huomaa, että siinä on semmosia...

Puhuja 5 [00:45:48]: Tiedon kulku varmaan kattaa...

Puhuja 4 [00:45:53]:

Puhuja 3 [00:45:53]: Ei tieto kulje kyllä välillä, vaikka ollaankin pieni yksikkö.

Puhuja 5 [00:45:55]: Niinpä. Siihen nähden kulkee tosi huonosti.

Puhuja 4 [00:46:01]: Kaikista paras, oliko se viime viikolla, kun tuli hätäleikkaus. Viittä vaille 6 tulee hoitaja käytävällä vastaan ja sanoo, että tämä on se hätäleikkaus, joo, moikka ja hei, kun meillä on semmonen vitsi, että hätäleikkaus. Niin hän sanoi, että ihan oikeasti. Ai jaa, kun minä olen lähdössä töistä pois.

Puhuja 5 [00:46:27]: Tämä korona-aika ei ole varmaan helpottanu yhtään sitä asiaa, kun on kaikki osastotunnit jäänyt pois.

Puhuja 1 [00:46:35]: KOrona ei kyllä helpota mitään asiaa.

Puhuja 3 [00:46:37]: Ei.

Puhuja 1 [00:46:42]: Mutta kun te puhuitte noista väliportaan esimiehistä, niin miksi juuri heille pitäisi olla erityisesti, onko ne tuoreimpia talossa vai?

Puhuja 3 [00:46:50]: Ei, kyllä meillä on ihan pitkän linjan tekijöitä nekin, mutta aina välillä tulee semmonen tuntemus, että olisko ne vähän ylikuormitettuja...

Puhuja 5 [00:47:00]: On.

Puhuja 3 [00:47:00]: Ja sitten on niin monenlaista sälää heillä, että vaikea edes tietää, että mitä kaikkea juttuja niillä pitäisi olla hanskassa ja välillä se tuntuu, että se asia pitäis olla hanskassa siellä ehkä vähän ylemmällä portaalla, eikä välttämättä kaikki siinä väliportaalla. Niillä on niin moneen suuntaan niitä lankoja, että solmuun menee välillä. Minä en osaa sanoa edes kaikkia ongelmia, mutta se vähän heijastuu meillekin päin, että sen takia toivoisin, että heitä haastateltais omana yksikkönään.

Puhuja 1 [00:47:41]: Onko muilla samanlaisia ajatuksia?

Puhuja 5 [00:47:43]: On joo.

Puhuja 3 [00:47:43]: Niillä voisi olla hyviä kehittämisideoita.

Puhuja 5 [00:47:47]: Ehdottomasti.

Puhuja 1 [00:47:58]: Se on varmaan semmonen epäkiitollisin paikka se.

Puhuja 3 [00:48:04]: Kyllä.

Puhuja 5 [00:48:11]: Tuosta hätäleikkauksesta tulee mieleen se satu, että susi tulee, susi tulee, että ihan oikeesti tulee, eikä kukaan tiedä, että miten käy.

Puhuja 3 [00:48:23]: Varsinkin, jos [?? 00:48:23] käytetä.

Puhuja 2 [00:48:37]: Viimeksi, oliko se pikkujouluissa, no mutta kuitenkin, jouduin esittämään naista ja se kerrottiin 0,5 h ennen esitystä. Sun hahmo on sitten nainen.

Puhuja 5 [00:48:56]: Hyvin sä vedit. Mun mies oli siinä (...) ja mun esimies. Aika vaivaannuttava. Se varmaan se vitsi olikin...

Puhuja 5 [00:49:26]: Tuonko mä sulle sen lomakkeen vielä?

Puhuja 1 [00:49:31]: Voisit antaa sille (...)

Puhuja 5 [00:49:33]: (...) joo. Pitäähän se nyt olla, että on kaikki luvat kunnossa.

Puhuja 1 [00:49:38]: JOs teillä ei ole mitään lisättävää, niin me voidaan lopetella. Kiitos tosi paljon, kun osallistuitte. Voin viedä (...) tietoa, että haluatte myös välitietoa tämän etenemisestä.

Puhuja 3 [00:49:50]: Ilman muuta.

Puhuja 1 [00:49:51]: Kiitoksia.

Puhuja 1 [00:16:06]:

[recording ends]

Name of recording: Haastattelu_1_2022.mp3

Length of recording: 00:34:33

Information:

[?] = Word was not understood completely but meaning is almost correct. Recording point is written to the text e.g. [word? 00:15:44]

[??] = Word could not be understood and therefore could not be written. Recording point is written to the text e.g. [?? 00:15:44]

[text] = Sounds or not transcribed parts are written with square brackets e.g. [laughs] or [interview paused due to a phone call]

[recording starts]

Puhuja X [00:00:02]: [?? 00:00:02]

Haastattelija 1 [00:00:03]: No mitä te nyt aattelette tästä tälleen, ku kahen vuoden tauon jälkeen, nii mitä te aattelette tästä laatujärjestelmästä täällä (…)? Onko se missä vaiheessa tällä hetkellä?

Puhuja 1 [00:00:17]: Jaaha.

Puhuja 1 [00:00:20]: [?? 00:00:19] että mitenkä menny, onko se mennyt eteenpäin.

Haastattelija 1 [00:00:26]: Nii et ole huomannut vai?

Puhuja 1 [00:00:27]: [Päällekkäinen puhe 00:00:27]

Haastattelija 1 [00:00:30]: Mitäs?

Puhuja 2 [00:00:31]: No mulla ihan sama vastaus oikeestaan, en oo muistanu laatujärjestelmää enkä oo nähny sitä esillä olevan missää vaiheessa, eikä oo multa kysytty mitään kylläkään, myöskään ja.

Puhuja 2 [00:00:45]: Vasta nyt, kun tuli tää kutsu tähän toiseen, nii muistin, et ai juu, tämmönen oli jotenki tekeillä.

Haastattelija 1 [00:00:50]: Nii?

Puhuja 2 [00:00:51]: Et käytännössä ei oo näkyny mun mielestäni.

Haastattelija 1 [00:00:55]: Joo.

Puhuja 2 [00:00:57]: Eikä kuulunu.

Puhuja 1 [00:00:57]: Joo, yleensä aika paljoki [?? 00:00:59] sähköpostin kautta tulee viesti, mut ei [Päällekkäinen puhe 00:01:02]

Haastattelija 1 [00:01:03]: Nyt ei oo tullu ollenkaa?

Haastattelija 1 [00:01:06]: Mut onks teil ollu nyt tässä sen jälkee, ku viimeks mä olin teitä haastelemassa, nii sen jälkee mitää orientaatioo tai koulutuksia tai mitään, mikä ois liittyny siihen laatujärjestelmään?

Puhuja 1 [00:01:15]: [Päällekkäinen puhe 00:01:16]

Puhuja 2 [00:01:16]: Ei yhtään mitään.

Haastattelija 1 [00:01:17]: Ei? Nii, et ei oo, ei mitää? Nii?

Puhuja 2 [00:01:18]: [Päällekkäinen puhe 00:01:18] ei missää vaihees [Päällekkäinen puhe 00:01:19]

Puhuja 2 [00:01:21]: Muistaakseni, muisti on toki rajallinen, mutta luulis, et ois jääny mielee, jos ois jotain ollu, kysytty tai ehdotettu tai jotai.

Haastattelija 1 [00:01:31]: Nii, et voidaa aatella, et ollaa oikeestaa samas kohassa ku sillon pari vuotta sitte?

Puhuja 2 [00:01:36]: Sillonki oltiin ymmällämme.

Haastattelija 1 [00:01:37]: Nii, kyllä, just näin, niin joo, sillonki oli sitä, että mitä tämä on.

Haastattelija 1 [00:01:42]: No ootteks te kerenny miettiä sen jälkeen, ku viimeks juteltiin tästä asiasta, et mitä se vois hyödyttää, tämmönen laatujärjestelmän rakentamine?

Haastattelija 1 [00:01:56]: [?? 00:01:55] odotuksia, ku viimeks siitä juteltii, nii jäiks teille sen jälkee mielee jotai, että?

Haastattelija 1 [00:02:03]: Minkälaisia odotuksia siihen liittye ois?

Puhuja 2 [00:02:05]: Tiedä, onko tää odotus, mut mä aattelin, et se on ainaki sitten ku markkinoidaan (…) palveluita, nii se on varmaa hyvä semmonen niinku tavallaan lisä, laadullinen lisä siinä, että tietää, et me tavallaa seurataan prosesseja ja toimintoja jotenki ja tämmöstä, että.

Haastattelija 1 [00:02:20]: Nii.

Puhuja 2 [00:02:22]: Tämmönen tuli mieleen, tavallaan tämmönen niinku hyötynäkökulma vähän uloski päin.

Puhuja 1 [00:02:29]: Ja nyt mitä viime aikoina palveluseteleitä kans tullu [?? 00:02:32] tänne ja, eli se on se markkinointi, et siel on kolme paikkaa, (…), (…) ja oisko (…), nii ainaki mitä käyty läpi vuodeosastolla, nii aika paljo ihmisiä (…) sairaala.

Haastattelija 1 [00:02:51]: Nii.

Haastattelija 1 [00:02:54]: Missä se, tiiätkö mihin se liittyy, et ne valitsee juuri (…)?

Puhuja 1 [00:02:59]: Se on vaan niiden henkilökohtainen valinta varmaa ja.

Puhuja 1 [00:03:04]: [?? 00:03:03] et ei siellä tarkemmin, siel sitten kerrota, et miks.

Puhuja 2 [00:03:09]: Toki voi toivoa, et se liittyy maineeseen, hyvään maineeseen, että tavallaan on siinä pohjana [?? 00:03:14]

Puhuja 1 [00:03:09]: [Päällekkäinen puhe 00:03:09]

Haastattelija 1 [00:03:11]: Nii, hyvä, nii.

Puhuja 1 [00:03:13]: Kyllä, jep.

Haastattelija 1 [00:03:17]: Ootteks te aatellu, et sillä vois olla ihan sinne toiminnan laatuun, et mitä se laatu nyt sitte ikinä onkaa, nii merkitystä?

Puhuja 2 [00:03:25]: Niin, tällä laatujärjestelmällä, mikä ettei, joo ja ainaki ihmisten mielikuvissa voi olla, niinku mä totesin alussa, että jos ihmiset aattelee tai kokee ja potilaat tietää, että me seurataan omaa toimintaamme jollaki, niin.

Puhuja 2 [00:03:40]: [?? 00:03:40] vähä niinku tutkimuksenki teko, ku me julkastaan aika paljo tutkimuksia vuosittain, nii onhan se semmonen, mitä kaikki ei tee, että se on tavallaa semmonen niinku.

Puhuja 2 [00:03:50]: Vähä niinku kiinnostuksen näkökulma asioihin myöski, halutaan tietää ja varmaan sitä myötä myös sitten parantaa toimintojamme.

Haastattelija 1 [00:04:00]: Kyllä.

Puhuja 2 [00:04:01]: Ja ainakin ihmisten mielikuvissa.

Haastattelija 1 [00:04:09]: [?? 00:04:08] varmaan monessakaan, niinku just mietin tätä laatujärjestelmän pystyttämistäki, niinku täs tehdään samalla tän tutkimusta ja selvitystä, nii tää on mun mielestä ihan niinku jotenki hyvä lähtökohta, et niinku oikeesti samalla kerätään tietoo siit, et miten henkilökunta kokee, että.

Puhuja 2 [00:04:22]: Joo.

Haastattelija 1 [00:04:23]: Iha positiivista.

Haastattelija 1 [00:04:26]: No ootteks te miettiny, et jos nyt se [?? 00:04:28] järjestelmä nyt koronan hiipuessa toivottavasti tois, tuoko se teidän omaan työhön jotai muutoksia?

Haastattelija 1 [00:04:37]: Jos se nyt alkais jotenki enemmän näkymää?

Puhuja 1 [00:04:46]: No ainaki, siis jos [?? 00:04:48] ei se korona mitenkää ainakaa vaikuttanut [Päällekkäinen puhe 00:04:53]

Haastattelija 1 [00:04:53]: Nii, se ei vaikuta [?? 00:04:54] laatujärjestelmä tulis kunnolla käyttöön, nii vaikuttaako se teiän?

Puhuja 1 [00:05:02]: No en uskoisi.

Haastattelija 1 [00:05:05]: Mites sun työhö?

Puhuja 2 [00:05:07]: Mun on vaikee ajatella, miten se vaikuttais.

Puhuja 2 [00:05:11]: Oikeestaan siitä syystä, et mä oon jotenki niin omalla alueellani jotenki, et mä teen, mä oon (…) ja teen niitä konsultaatioita, on poliklinikkapotilaita ja sitten on kuntoutujia ja.

Puhuja 2 [00:05:25]: Ja mul on omat työmenetelmäni ja jotenki, et ja tapani, niinku struktuurini tehdä omaa työtäni, nii mun on vaikee siihen niinku.

Puhuja 2 [00:05:36]: Et ymmärtäisin, et siihen tulis jotain muutosta.

Puhuja 2 [00:05:39]: Ja vähän aattelisin, että on vaikee myös muitten tulla sanomaan, että tätä voi parantaa, tai nyt en tarkota sitä, et mä olisin kritiikille pelokas vaan sitä, että muut ei tiedä oikeen, miten mä teen mun omaa työtäni ja mitä siihen liittyy, et mitkä ne mun työkalut on mun työkalupakissa.

Puhuja 2 [00:05:57]: Se on tavallaa nii pitkälle viety semmone erikoistumispolku jo.

Haastattelija 1 [00:06:01]: Joo.

Puhuja 2 [00:06:02]: Et aika sillee niinku autonominen oon, että jos jotain ehdotetaan tai pyydetään pohtimaan, nii aina olen siihen tietenki valmis, kyllä.

Haastattelija 1 [00:06:13]: Onko teitä monta vastaavaa työtä [?? 00:06:15]

Puhuja 2 [00:06:16]: Mä oon ainoa [Päällekkäinen puhe 00:06:16]

Haastattelija 1 [00:06:16]: No joo, no sitte, joo.

Puhuja 2 [00:06:18]: Joka tekee tätä, just tätä työtä, että.

Puhuja 1 [00:06:23]: No meil meil on sit paljo enemmä osastonhoitajia, hoitajat, kuka hoitaa just nää, palveluseteleiden kans potilaat ja.

Puhuja 1 [00:06:34]: Kuka hoitaa nimenomaan (…) potilaita, nii yksityisiä.

Puhuja 1 [00:06:39]: Se on sit, et siel on vähä eri linjat ja no.

Puhuja 1 [00:06:44]: Se hoitoprosessi on.

Puhuja 1 [00:06:47]: Sielläki on vähä eri tavalla.

Haastattelija 1 [00:06:55]: No missä vaiheessa teiän mielestä toi laatujärjestelmä on (…)?

Haastattelija 1 [00:06:58]: Onks se edelleen ihan alussa niinku sillon kaksi vuotta sitten?

Puhuja 2 [00:07:01]: En tiedä.

Puhuja 2 [00:07:04]: Mul ei oo mitään tietoa siit.

Puhuja 1 [00:07:04]: [Päällekkäinen puhe 00:07:04] siis ku.

Puhuja 1 [00:07:06]: Ainaki sähköpostin mukaan, mitä [?? 00:07:08] viestiä tulee [?? 00:07:09] se homma, mitä.

Puhuja 1 [00:07:12]: [Päällekkäinen puhe 00:07:12]

Puhuja 2 [00:07:12]: Onko tullu mitää viestiä tästä [?? 00:07:14]

Puhuja 1 [00:07:14]: Ei.

Puhuja 2 [00:07:14]: No ei mullekaa, näin, mul on sama mielikuva, että.

Puhuja 1 [00:07:16]: [Päällekkäinen puhe 00:07:16] joka, ei ihan joka päivä, [?? 00:07:18]

Haastattelija 1 [00:07:23]: No jos aateltas tätä, laatujärjestelmä tulis tässä [?? 00:07:26] enemmän käyttöö, niin näättekö te teiä, varsinki osastotyössä, nii onks sillä tekemistä työturvallisuuden kanssa?

Haastattelija 1 [00:07:45]: Tai potilasturvallisuuden?

Puhuja 1 [00:07:46]: [?? 00:07:46]

Puhuja 1 [00:07:51]: No.

Puhuja 1 [00:07:55]: Nii, siis [?? 00:07:56] et siis potilaan kannalta?

Haastattelija 1 [00:07:58]: Nii.

Puhuja 1 [00:08:00]: No.

Haastattelija 1 [00:08:00]: Tai oman, sun oman työn kannalta?

Puhuja 1 [00:08:01]: Oman, joo, kyllä, varmasti on.

Haastattelija 1 [00:08:04]: Minkälaisia?

Puhuja 1 [00:08:07]: [?? 00:08:08]

Puhuja 1 [00:08:10]: No jos vaikka jotain tapahtuu, vaikka [?? 00:08:14] tapaturma [Päällekkäinen puhe 00:08:14] sitte mä tiedän, miten, mitä sitte seuraavaksi pitäs tehä ja, että niitä ohjeita on kyllä annettu ja me aina välillä niitä käydää läpi osastotunnillaki.

Haastattelija 1 [00:08:34]: Sulla ei varmaan oo mitään semmosta laatujärjestelmää vai onko?

Puhuja 2 [00:08:38]: Ei varmaan tän tällaista, että.

Puhuja 2 [00:08:41]: Itse vastaan työhyvinvoinnistani ja olen vastuussa potilaitten hyvinvoinnista siinä mielessä, et en mokaa omassa työssäni, en töppäile pahemmin, että.

Puhuja 2 [00:08:56]: Oikeastaan perusperiaatteet, josta lähdetään tätä työtä tekemään, nii ne on jotenki mukana.

Haastattelija 1 [00:09:03]: No aatteletteko te, että laatujärjestelmäl vois olla yhteyksiä työn motivaatioo? Voisko se parantaa teiän työssä viihtymistä tai motivaatioo tehä työtä?

Puhuja 2 [00:09:18]: Tää nyt on naftia, tää mun vastaukseni, mut en pysty sanomaan [Päällekkäinen puhe 00:09:21] vaikuttais oikeen, että.

Puhuja 2 [00:09:25]: Tuntuis sitte semmoselta, vähä niinku päälle liimatulta, et jos nyt lähdetään tätä mun työnkuvaa miettimään ja nyt niinku lähdetään jotain tästä parantamaa, nii en näkis, et se oikeestaan olis sillä niinku tekemisen polulla, et se ois vähä niinku semmone.

Puhuja 2 [00:09:41]: Jo vähän itse tarkoituksellinen jotenki, et.

Puhuja 2 [00:09:46]: En poissulje sitä, ettei ois jotai, mutta nyt ei kyllä tuu mieleen, että mitä se vois olla.

Haastattelija 1 [00:09:53]: Mites sun työssä?

Puhuja 1 [00:09:56]: [?? 00:09:55] palveluseteleiden potilaita on paljo enemmän, siis tulee jatkuvasti, nii toki se työ on lisääntynyt sit sen mukaan, et.

Puhuja 1 [00:10:09]: Että on tarvittu sitte lisähoitaja sinne vuodeosastollekin.

Puhuja 1 [00:10:17]: Ja muutenki, siis.

Puhuja 1 [00:10:20]: [?? 00:10:20] mitä aikaisemmin ollut, et paljo enemmän töitä [?? 00:10:24]

Haastattelija 1 [00:10:25]: Niin, ku on erilaisia potilaita, ku tulee?

Puhuja 1 [00:10:27]: Erilaisia, nii, juu, kyllä.

Haastattelija 1 [00:10:30]: Kaupunki siis antaa niitä palveluseteleitä ja sitte niillä tulee?

Puhuja 1 [00:10:32]: [Päällekkäinen puhe 00:10:32]

Haastattelija 1 [00:10:33]: Kyllä, just näin.

Haastattelija 1 [00:10:37]: No mitä te aattelette, mikä vois olla työhyvinvointii liittyvä tekijä, mikä vois edes liittyä tähä laatujärjestelmää?

Haastattelija 1 [00:10:44]: Näätteks te [?? 00:10:44] mitää yhteyttä?

Puhuja 2 [00:10:49]: No tää nyt on tämmöne laadullinen, siis mulla tää mielikuva, mutta jos esimiestyö ois semmosta, että ois aitoa semmosta pohdintaa ja vuorovaikutuksellista tämmöstä, sanotaa ny vaikka suoraa näin, et esimies ymmärtäisi, mitä työtä mä teen.

Puhuja 2 [00:11:05]: Mul on (…) esimiehenä ja mä oon (…).

Puhuja 2 [00:11:09]: Ja teen tutkimusta, mul on (…) melkein riittävä määrä kansainvälisiä julkasuja, nii se kuilu on niin iso, et ei oikeen synny semmosta, mikä mua motivois oikeen.

Puhuja 2 [00:11:20]: Siis semmosta.

Puhuja 2 [00:11:21]: Eikä voi olla [?? 00:11:22] fysioterapeutin näkökulmasta, nii ei oo tämmöstä ymmärrystä, mitä esimerkiks semmone potilastyö-vuorovaikutus ja transferenssit, vastatransferenssit, mikä se dynamiikka on siinä hoitosuhteesa, nii ei oo vaan sitä työkalua esimiehellä.

Puhuja 2 [00:11:39]: Esimiehillä.

Puhuja 2 [00:11:41]: Nii jotenki semmone, et ois tämmöne kollegiaalinen mahdollisuus esimiehen kans, mut sitä ei nyt oo.

Puhuja 2 [00:11:48]: Et vähän sillee niinku yksinäinen susi olen jotenkin, mut se ois yks ainoa, tavallaa semmonen, että voisin täällä, no mul on tietenki muita [?? 00:11:58] työnohjaukset ja kaikki tämmöset, mä itekin ohjaan myös muita sitte, et siel tavallaa semmonen läpituulettuva virta, jos sä oot, niinku ajatukset on niinku.

Puhuja 2 [00:12:09]: Miten sanois? Niinku tuoreena ja tämmöstä, mutta se ois semmone kiva juttu.

Puhuja 2 [00:12:17]: Mä muistan aikoinaa, ku mä itte olin, mä olin 11 vuotta johtavana psykologina ja sillon oli myös enimmillään varmaan 16 alaista.

Puhuja 2 [00:12:27]: Nii jokaisen kans käytiin semmosta [?? 00:12:28] hurjan filosofista ja syvää pohdintaa, et miltäs tuntuu ja mitkä on ne omat karikot itse kullaki ja tämmöstä, nii se nyt puuttuu, mut se on se, tavallaa se on se organisaation todellisuus tällä hetkellä, kun ei oo muita.

Puhuja 2 [00:12:43]: [Päällekkäinen puhe 00:12:43]

Haastattelija 1 [00:12:43]: Mut kuka se vois olla, et jos miettii tota esimiestyötä? Kuka se vois olla lähimpänä niinku sun?

Puhuja 2 [00:12:49]: No (…) kanssa ollaan, (…) oli mun (…).

Puhuja 2 [00:12:52]: (…) oli mun esimiehenä muutaman vuoden.

Puhuja 2 [00:12:56]: Ja sillon tavallaan aina oli niinku liikkumatilaa ja tämmöstä pohdinnan mahdollisuutta ja luottamusta oli (…) myös hyvin paljo mun, nyt mä en edes tiedä sitä, että onko (…), (…), fysioterapeutilla luottamusta, koska meil ei oo sitä dialogia oikee.

Puhuja 2 [00:13:17]: Mut semmonen tavallaa, et tultaisiin tutuiksi, että esimieskoulutuksensaki puolesta ymmärtäisi jotai.

Puhuja 2 [00:13:27]: Ja sit tuli nyt tässä vielä ohessa mieleen tämmönen tutkimuksen teko, että ne jotka ei tee tieteellistä tutkimusta, niin niiltä puuttuu kyllä myös semmonen, tavallaan ymmärrys siihen isoon prosessiin, miten hurjan paljon työtä tehdään ennen ku saadaan joku julkaisu, niin sitä me tehdään tässä rinnakkain tai sitte koulutusta, me tehdään ihan hyviä seminaareja, (…) seminaarit on semmosia maankuuluja kyllä, että, ja arvostettuja, niin sitäkää en oikeestaa.

Puhuja 2 [00:13:55]: Tuntuu välillä, että ne lähiesimiehet ei tiedä sitä isoa ja moniulotteista työtä ja sitä [?? 00:14:04] mitä se tarvii, se työ, jotta saadaan hyvä seminaari rakennettua, suunniteltua ja luotua ja hyvät luennoitsijat ja tämmöstä.

Puhuja 2 [00:14:13]: Itte, ku mä istun kolmella, jakkaran kolmella, et mul on potilastyö, sit mul on tää seminaarien ja tää opetustyö ja sitten tää tutkimus, nii mulle se on tavallaa semmone hyvä, mutta.

Puhuja 2 [00:14:26]: Mut oikeestaan kumpaan, nyt ku mä mietin, yhteenkään näistä ei tuu sitä ymmärrystä esimiehellä.

Puhuja 2 [00:14:33]: Ja se johtuu koulutuksesta, se johtuu varmaan monista muista jutuista.

Puhuja 2 [00:14:37]: Se nyt vaan on näin, en tiedä, tulisko laatujärjestelmä tähän jotenkin, että huolehdittaisiin siitä, että lähiesimiestyö on myös laadullisesti, ei vain määrällisesti, et saadaan maskeja ja kopiopaperia jostai ja tämmöstä, et ois oikeesti semmonen tunteellinen ja niinku laadullisella puolella semmonen pohdinta, nii.

Puhuja 2 [00:15:00]: Mut jotenki huomaan nyt olevani vähän yksinäinen, ku mä tätä kerron, nii tää tämmöne, tämmösiä ajatuksia.

Haastattelija 1 [00:15:09]: Ooks sä yrittäny jutella muille kuin lähi, tai no varmaan ei lähiesimiehelle, mutta niinku ylemmille?

Puhuja 2 [00:15:16]: No hei, meil on hyvin toimivat keskustelukontaktit eri suuntiin, et ei se ny sillai, mutta jos nyt miettii oikeesti työtä ja esimiestyötä ja tämmöstä, nii tää on semmone relevantti kysymys.

Haastattelija 1 [00:15:27]: Kyllä.

Puhuja 2 [00:15:30]: Et mielelläni itteni näkisin vaikka (…) alaisena tai.

Puhuja 2 [00:15:34]: Se ois niin kun jotenkin parempi ratkaisu.

Haastattelija 1 [00:15:40]: Onks sulla tämmösii vastaavia kokemuksia tai miten ajattelet tän?

Puhuja 1 [00:15:43]: [?? 00:15:43] kuitenki vuodeosaston [?? 00:15:45] työnkuva ja esimies, meil on vuodeosastolla [?? 00:15:53] tavallaa samalla sitte jonohoitaja, et hänel on aika paljo muita tehtäviä ja.

Puhuja 1 [00:16:01]: Se se nyt on auki.

Puhuja 1 [00:16:04]: Työpaikka on auki tossa esimiehen tai vuodeosaston hoitajan.

Puhuja 1 [00:16:11]: Just selkeet, et niin paljon työtä, et hän ei kerkeä kaikkea.

Haastattelija 1 [00:16:15]: Nii.

Puhuja 1 [00:16:17]: Tehä, nii.

Puhuja 1 [00:16:20]: Toki nää on, vuodeosastotki [?? 00:16:21] et.

Puhuja 1 [00:16:23]: Siel on semmosta, noista keskustelua, noista vuodeosaston asioista, että ja.

Puhuja 1 [00:16:33]: Muuta, et.

Puhuja 1 [00:16:34]: [?? 00:16:34] tuli mielee, meil on niitä, Epassi on käytössä ja.

Puhuja 1 [00:16:40]: Se kyllä.

Puhuja 1 [00:16:49]: On siis, [?? 00:16:50] on käytössä [Päällekkäinen puhe 00:16:52]

Haastattelija 1 [00:16:56]: Nii, se Epassi on [?? 00:16:57] työhyvinvointiin liittyvänä tekijänä, jos voi niinku mennä tekee kulttuuririentoja tai liikuntaa tai?

Puhuja 1 [00:17:02]: Nii, joo.

Haastattelija 1 [00:17:04]: Onko tota, onks teillä? Mitä muuta te aattelette [?? 00:17:06] työhyvinvointi, hyvinvoinnin kantavina voimina, että onks teillä jotain muita ajatuksia, että mikä auttaa siinä työhyvinvoinnissa, et jos [?? 00:17:15] et tää laatujärjestelmä voi olla yks, jos se saatas toimimaan, nii?

Haastattelija 1 [00:17:21]: Onks muita niinku pilareita?

Puhuja 2 [00:17:26]: Nii.

Haastattelija 1 [00:17:29]: Hyvät keskusteluyhteydet?

Puhuja 1 [00:17:29]: [Päällekkäinen puhe 00:17:30]

Puhuja 2 [00:17:30]: Nii, se mitä jo oikeestaan on aika, siis meillähän on hurjan hyvä tiimityö jotenki, että se on kyllä semmone, josta myös (…) on varmaan jotenkin eniten erottautunu muista firmoista jotenki siis] kuntoutustiimistä.

Puhuja 1 [00:17:45]: Joo.

Haastattelija 1 [00:17:46]: Joo.

Puhuja 2 [00:17:47]: Et se on tosi hyvä ja hyvät dialogit ja avoimet pohdinnat ja tiimipohdiskelussa aina tiimitapaamisella tai kokouksessa aina niinku se tieto karttuu, että se on joka kerta näin itse kullakin, et jotenki rakentuu se ymmärrys potilaaseen niinku joka kerta.

Haastattelija 1 [00:18:06]: Onko teillä aina potilaskohtaset ne keskustelut siinä [Päällekkäinen puhe 00:18:09]

Puhuja 2 [00:18:10]: Joo, me käydään ne läpi, ne jotka on kuntoutujia, siis jos on poliklinikkapotilaita, nii sit on satunnaisesti vaikka lähettävän lääkärin kans jotain tai joskus sairaanhoitajankin kans, jos on näin, mutta aika harvoin.

Puhuja 2 [00:18:21]: Tai fyssarin kans, mut sit jos on kuntoutujina täällä joku porukka, nii heiät käydään aina kerran viikossa läpi [?? 00:18:29]

Puhuja 2 [00:18:31]: Kyllä jokainen vuorotellen ja silleen.

Puhuja 2 [00:18:36]: Mut sillon ei oo potilaat läsnä, että se ei oo semmonen.

Haastattelija 1 [00:18:38]: Nii, et voi jutella ihan niinku kollegan?

Puhuja 2 [00:18:41]: Kyl, juu, just näin.

Haastattelija 1 [00:18:42]: Joo.

Puhuja 1 [00:18:46]: Meil on vuodeosastolla sit niin, et tota.

Puhuja 1 [00:18:49]: [?? 00:18:49] et siis tosiaan hoitajat viihtyy täällä ja.

Puhuja 1 [00:18:54]: [Päällekkäinen puhe 00:18:54]

Haastattelija 1 [00:18:54]: Mistä te päättelette, että hoitajat viihtyy?

Haastattelija 1 [00:18:57]: Mä mietin nyt tätä laatujärjestelmää, et mistä sitä saa, niinku mistä nurkasta [?? 00:19:00]

Puhuja 1 [00:19:00]: [?? 00:19:01] tiimityö [Päällekkäinen puhe 00:19:02]

Puhuja 1 [00:19:05]: [?? 00:19:05] sitte siitä voi keskustella ja.

Puhuja 1 [00:19:10]: No ainaki yli.

Puhuja 1 [00:19:11]: Paljo mä oon ollu täällä? 12 vuotta, nii.

Puhuja 1 [00:19:14]: Oon toki, mut on siis, et täältä hoitajat pääsevät eläkkeelle [?? 00:19:19] työpaikkoja.

Puhuja 1 [00:19:23]: [?? 00:19:22] tulee, tuli sitte uusien tilalle ja.

Haastattelija 1 [00:19:27]: Se on hyvä.

Haastattelija 1 [00:19:29]: Jos mietitään tätä laatujärjestelmää, et mihin tää vois niinku auttaa sitten siinä niinku työhyvinvoinnissa, nii varmaan se, et niinku jotenki.

Haastattelija 1 [00:19:36]: Niinku [?? 00:19:36] laadullisesti tai jotenki, et ois semmonen niinku systeemi siihen.

Haastattelija 1 [00:19:42]: [?? 00:19:42] esimiestyöhön, että [?? 00:19:44] vaikee [?? 00:19:45] niinku jos ei ole, eihän laatujärjestelmällä pysty tekemää semmosia asioita, mitä ei oikeesti ole olemassa, et sä voit vaan niinku järjestää sitä, mikä on ja niinku sopia jostain tietyistä yhteisistä pelisäännöistä.

Puhuja 2 [00:20:01]: Nii, jos tää nyt on tämmönen vähän organisaatiopsykologinen juttu, mutta jos alainen vois opettaa esimiestään enemmän siitä, missä mennää ja tavallaa miten tehdään töitä ja tämmöstä, niin et se ois niinku mahdollisimman kivasti ylöskin päin menevä, se viesti, että [?? 00:20:17] nii se ois kyllä semmone.

Haastattelija 1 [00:20:17]: Niin, kyllä.

Puhuja 2 [00:20:20]: Sehän on ollu semmonen aikoinaan sillon ku mä ite olin johtavana psykologina, niin mä vein koko kollegakunnan ja alaisteni ajatuksia aina (…) ja mietittii ja mä sanoin, et nyt tämmönen täytyy saada läpi, et me ollaan porukalla tätä mieltä, et tää ei nyt toimi.

Puhuja 2 [00:20:37]: Sillon oli ylös päin se, mutta nyt tuntuu siltä, et se pysähtyy siihen.

Puhuja 2 [00:20:42]: Seuraavalle asteelle jotenki, että.

Haastattelija 1 [00:20:43]: [?? 00:20:43] miettii, et siin laatujärjestelmäs vois olla semmost, et miten niitä asioita viedään niinku molempiin suuntiin, niinku semmonen järjestelmä?

Puhuja 2 [00:20:49]: Joo, kyl mä näin, et se hengittää, se systeemi, et [?? 00:20:51]

Puhuja 2 [00:20:52]: Eri asia on sitte, mitä voidaan läpi viedä niistä ajatuksista ja kommenteista, feedbackista, että mikä johtaa mihinki, mut et se väylä ois jotenki auki ja olemassa ja auki.

Puhuja 2 [00:21:04]: Et se ois niinku tärkeetä.

Haastattelija 1 [00:21:09]: Kyllä.

Haastattelija 1 [00:21:14]: No te kummatkaa ette oo töissä leikkauksii liitty-, tai mis-, niinku, paitsi nyt tietysti osastolla tulee leikkauspotilaita?

Puhuja 1 [00:21:20]: Joo.

Haastattelija 1 [00:21:21]: Mut aatteletko, että ne vois vaikuttaa jotenki leikkauspotilaitten hoitamiseen tai johonki tonne laatujärjestelmää?

Puhuja 1 [00:21:35]: Musta ei.

Haastattelija 1 [00:21:35]: Ei?

Haastattelija 1 [00:21:40]: No [?? 00:21:40] jotain muuten siihen potilaasee liittyvii, potilaa hoitoo liittyvii asioi, mihin se laatujärjestelmä vois olla hyödyks tai?

Puhuja 1 [00:22:00]: Jos oli se joku [?? 00:22:00] semmonen [?? 00:22:01] semmone järjestelmä, nii.

Puhuja 1 [00:22:11]: Tavallaa nykysi on se vaa, se, jos aikasemmin oli plastiikkakirurgia ja neurokirurgi [?? 00:22:16]

Puhuja 1 [00:22:19]: [?? 00:22:20] potilaita vuodeosastolla, nii.

Puhuja 1 [00:22:25]: [?? 00:22:24] ja siel on, se prosessi on tuttuu kaikille ja.

Puhuja 1 [00:22:31]: En mä oikee, mä en [?? 00:22:32]

Puhuja 1 [00:22:35]: Miten se järjestelmä voi auttaa siihen [?? 00:22:37]

Haastattelija 1 [00:22:39]: Nii, et just se, et se prosessi on tuttu kaikille ja on yhteiset ohjeet, kaikki tietää, miten, mitä tehdää?

Puhuja 1 [00:22:44]: Joo.

Puhuja 1 [00:22:45]: Niin se on, koska se on semmonen.

Puhuja 1 [00:22:48]: Kaikki nää leikkaukset, on suunniteltu leikkaukset ja.

Puhuja 1 [00:22:51]: Ei tässä mitää semmosta.

Puhuja 1 [00:22:55]: Ensiapuu [Päällekkäinen puhe 00:22:56]

Haastattelija 1 [00:22:57]: Nii, ei oo mitää yllättäviä leikkauksia, että joku tulis vaa?

Puhuja 1 [00:22:59]: Joo.

Haastattelija 1 [00:23:02]: No mitä te aattelette? Mä kysyin viimekski tän saman, et mitä te aattelette, miten tämmösel laatujärjestelmällä voi olla yhtymäkohtia (…) tulevaisuutee?

Haastattelija 1 [00:23:10]: [?? 00:23:10] alussa [?? 00:23:11] markkinoinnista ja maineesta, nii aatteletteko te, et se on niinku?

Puhuja 2 [00:23:17]: Ku se on varmaa semmonen pysyvä laatu, laadullinen juttu sitte, et jos tila ja tahot, potilaat tietää, että tarkkaillaan itteämme ja meil on tämmönen, tavallaan laatusysteemi tai seuranta kuitenki työssämme, nii onhan sillä markkinointiarvoa, että tiedetään, mitä tehdään jotenki ja.

Puhuja 2 [00:23:42]: Ois kiva tietää, et mikä on se alkuperänen niinku motiivi rakentaa tätä laatujärjestelmää ja ottaa sitä käyttöön, se jäi jotenki vielä vajaaksi sillon viimeksi, että sillon vois olla helpompi ainaki mun miettiä, et tavallaa, et mistä se tulee, mikä on se paine, mikä

on se motiivi ja alkukipinä, koska mun mielestä, niinku säkin sanoit, nii asiathan on pyöriny vuosikaudet hyvin rutiininomasesti ja turvallisesti siellä leikkurissaki ja niinku mullaki jotenki, että.

Puhuja 2 [00:24:10]: Että voisko olla kyse siitä, että joku, joka ei oo ollu tässä työssä kiinni, on ideoinu, nyt täytyy tämmönen saada jotenki ja sillon se tulee päälle liimatuks jotenki vähän, että jos se kumpuaa, se tarve porukan sisältä, et nyt meiän täytyy ruveta miettimää näitä laadullisia asioita ja tarkkailemaa ja seuraamaa, sillon se on ihan eri, se substanssi tavallaa.

Puhuja 1 [00:24:31]: Joo, kyllä.

Puhuja 2 [00:24:32]: Sillon se lähtee meiän tarpeesta, tavallaan liittyen siihen, että pitäs ajatusten mennä ylöspäin, nii tässäki varmaa, laatujärjestelmässä, nii näkisin hyvänä sen asian, et se lähtis meistä.

Puhuja 2 [00:24:45]: Et nyt lähtee niinku, nyt tulee työntekijät niinku vaatimalla laatusysteemiä, et nyt tähän laatusysteemi, nyt me tarvitaa sitä.

Puhuja 2 [00:24:53]: Nyt en tiedä, mistä se on tullu ja mikä sen lähtökipinänä on, että.

Puhuja 1 [00:25:01]: [?? 00:25:00] samaa mieltä, et [?? 00:25:01]

Puhuja 1 [00:25:06]: [?? 00:25:05] tosiaa siellä vuodeosastolla [?? 00:25:08] jos tulisi [?? 00:25:10] alueelta ja lapsipotilaita, nii sillon otettais taas sen Apotin käytön, niin oiskos siihen sitte jotain?

Puhuja 1 [00:25:20]: Tarvitaa, mutta siihenki tulee koulutusta ja tääl on aika semmone [?? 00:25:25]

Puhuja 1 [00:25:27]: Turvallinen ympäristö, että.

Puhuja 1 [00:25:30]: Aina tietää, mitä tehä ja.

Puhuja 2 [00:25:32]: Munkin on jotenki vaikea ajatella, et jotain tehtäisiin huonosti, jos se ei oo laatujärjestelmä, että kyllä silti pitää just sitä koulutusta, jos Apotti [?? 00:25:39] nii ei oteta käyttöö, mutta jos otetaan, nii totta kai siihen sitte koulutetaan ja.

Puhuja 1 [00:25:47]: Siis meil oli [?? 00:25:48] käytös sillon ku oli neuropotilaita ja plastiikka.

Puhuja 2 [00:25:51]: Okei.

Puhuja 1 [00:25:52]: Mut meil oli vaa se lukuoikeus [?? 00:25:55] jos olisi tulevaisuudes niitä lapsipotilaita, niin siihen tarvii toki enemmän koulutusta ja.

Puhuja 1 [00:26:07]: Nii ja sitte [?? 00:26:07] käyttö.

Puhuja 2 [00:26:11]: Joo.

Puhuja 1 [00:26:13]: Että okei, se on vielä [?? 00:26:14] tai se on [?? 00:26:15] tiedossa.

Puhuja 1 [00:26:19]: [?? 00:26:18] palveluseteleiden kanssa alkaa pyörimään tosi hyvinki, et paljon potilait on jonossakin.

Haastattelija 1 [00:26:28]: Nii, et ei tarvi sillee miettii, et työt loppus, että?

Puhuja 1 [00:26:30]: Joo, [Päällekkäinen puhe 00:26:30]

Haastattelija 1 [00:26:37]: No me ollaa oltu tosi nopeita nyt, mul oikeestaa loppu [?? 00:26:39] kysymykset, et mitä te muuta haluaisitte kertoo tästä laatujärjestelmästä tai?

Haastattelija 1 [00:26:43]: No teil ei [?? 00:26:43] kerrottamaa, mutta onko teillä ideoita, miten sitä nyt kannattas lähtee edistämään tai?

Puhuja 2 [00:26:52]: Jotenki aattelisin, et viestittämään meille, et missä mennään nyt ja mitä on ajateltu ja miten meiän edellinen haastattelu on vaikuttanu tähän laatu-, systeemiprosessiin, et miten sitä on hyödynnetty, onko se tullu siihen niinku rakennuspalikaksi vai onko se vaan niinku.

Puhuja 2 [00:27:08]: Onko nää irrallisii asioita vai liittyykö ne toisiinsa jotenkin, se ois ihan kiva tietää ja.

Puhuja 2 [00:27:14]: Ja miten tullaan tätä hyödyntämää nyt sitte, vaikuttaako se viestintää nyt kun me sanotaan molemmat yhteen ääneen, me ei oikeen tiedetä yhtään mitään, ei oo tullu viestiä eikä mitää, nii oisko tää semmone heräte sitte, et jos on jotai nyt muhinu, et on oikeesti kehitetty laatusysteemiä, nii oisko meiän hyvä saada tietää siitä?

Haastattelija 1 [00:27:35]: Iha hyvä.

Haastattelija 1 [00:27:37]: Hyvä viesti, tommone viesti, [?? 00:27:38]

Puhuja 2 [00:27:39]: Nii, semmone aika selkee [Päällekkäinen puhe 00:27:39] ei oo tietoa, jos on jotain, mitä meiän tarttis tietää, niin saataisiinko me sitä tietoa?

Haastattelija 1 [00:27:44]: Nii.

Puhuja 1 [00:27:47]: Mielummin sähköpostin kautta varmaan [Päällekkäinen puhe 00:27:49]

Puhuja 2 [00:27:49]: Nii, kyllä, se on ihan hyvä.

Puhuja 2 [00:27:53]: Sit jos herää lisäkysymyksiä, nii voi [?? 00:27:54]

Haastattelija 1 [00:27:55]: Niin, voi kysyä, että mite, kyllä.

Haastattelija 1 [00:27:59]: No onks teillä mitää ideoita tosta työhyvinvoinnin niinku [?? 00:28:03] laatujärjestelmää, et miten te niinku kehittäisitte sitä puolta tai?

Puhuja 2 [00:28:09]: Mä en tiedä, mulle tulee ny mieleen, kun on kevätkin ja aurinko paistaa, niin semmosta jotain pientä juhlintaa vois olla ainaki pari kertaa vuodessa ja vähän sitä, että vaikka henkilökunnalta kysyttäis, et mitkä ois tarpeet ja toiveet ja jotenki, että sehän on tavallaan hyvin.

Puhuja 2 [00:28:25]: Yllättävän iso osa työhyvinvointia, että voi välillä juhlia ja käydä vaikka skumppaa juomassa tai tanssimassa tai jotain tämmöstä, että.

Puhuja 1 [00:28:36]: Varmaan sen koronan takia on jäänyt aika [?? 00:28:38] mutta ainaki tos vuodeosastolla, oliko se joulukuun aikana? Oli, kyllä.

Puhuja 2 [00:28:46]: Joo.

Puhuja 1 [00:28:46]: Osallistuin siihen, mut tota joku [?? 00:28:49]

Puhuja 2 [00:28:50]: Oli meilläki joku semmonen ja jotkut läksiäisetkin on ollu yhden fyssarin, joka lähti eläkkeelle, niin jotain [Päällekkäinen puhe 00:28:56]

Puhuja 2 [00:28:59]: Toinen juttu, mikä nyt tuli mieleen, niin mul ei oo tietoa, että missä määrin [?? 00:29:02], niin voidaan käyttää tätä punttisalia.

Puhuja 2 [00:29:07]: Yksinkertainen, meil on aika hyvä, nähtävästi jossain rappusessa, nii hyvä punttisali tuolla ammattiopiston puolella tai jossai, nii mul ei oo siitä tietoo, että onko se auki ja mitenkä sinne pääsis, nii.

Puhuja 2 [00:29:19]: Ja pääseekö saunaan sen jälkeen, tämmösiä, meil oli aikoinaan tämmöstä psykolenkkia, muutaman kollegan kans käytiin lenkkeilemässä, sit käytiin vielä vähän jotain rautaa nostamas, sitte heittämäs löylyjä, nii se oli jotenki semmosta mukavaa.

Haastattelija 1 [00:29:34]: Nii [?? 00:29:34] kuulostaa.

Puhuja 2 [00:29:35]: Sen jälkeen oli hyvä olo ja rento olo.

Haastattelija 1 [00:29:38]: Kyllä.

Puhuja 2 [00:29:39]: Ja tää vois olla tää, tavallaan semmone, kun, jos on nyt hyvät laitteet täällä kuitenki, et on resurssit, niin mitenkäs saatasiin sitte nää resurssit yhdistettyä henkilökunnan tarpeisii jotenki.

Haastattelija 1 [00:29:53]: Niinpä.

Puhuja 1 [00:29:52]: Joo, meillä ainaki viesti tullut, et tavallaa se on vielä auki ja [?? 00:29:57] henkilökunnalta, että mikä [?? 00:29:58] sopiva kellonaika tai aikataulu, et millon sitä salia voi käyttää.

Puhuja 2 [00:30:02]: Okei.

Puhuja 2 [00:30:04]: Ei oo varmaan, ei kuulosta tutulta viestiltä, ei oo varmaa mulle asti tullu, mutta tässä voi olla.

Puhuja 2 [00:30:10]: Semmonenkin on ollu nyt, ku mietitään viestintää, nii semmosta, et joku on jääny pois viestinnästä myös sattumoisin, se ei ollu mitenkään ilkeesti ajateltu, mut ei oo vaa niinku muistettu ottaa mukaan johonki viestintäketjuu, nii.

Haastattelija 1 [00:30:22]: Joo.

Puhuja 2 [00:30:24]: Itse huomasin, että meiän sosiaalityöntekijä oli kokonaan ollu poissa yhdestä aika pitkästä viestiketjusta ja hän on kuitenki vankkana osana sitä kuntoutustiimiä.

Puhuja 2 [00:30:36]: Niin tämmöstä, että varmistutaan siitä, että kaikille, jotka on jotenki siinä mukana, tulee se viesti myös perille, että.

Puhuja 2 [00:30:43]: Eli tavallaan viestintää taas jotenki, että.

Haastattelija 1 [00:30:56]: Eikös teillä tos alakerrassa se uima-allaski? Saatteko te käyttää henkilökunta sitä?

Puhuja 1 [00:31:00]: Se on purettu.

Haastattelija 1 [00:31:01]: Ai se on purettu?

Puhuja 1 [00:31:02]: Joo.

Puhuja 2 [00:31:02]: Joo, siihen on.

Puhuja 1 [00:31:03]: [Päällekkäinen puhe 00:31:03]

Haastattelija 1 [00:31:03]: Mun mielest se oli viel sillon [?? 00:31:04]

Puhuja 2 [00:31:05]: Niin oli, joo ja ne rakentaa uutta ja ne on räjäytelly tota kalliota [?? 00:31:08]

Puhuja 1 [00:31:10]: Siinä tulee sitte se [?? 00:31:11]

Puhuja 2 [00:31:13]: Okei, joo.

Haastattelija 1 [00:31:14]: Okei

Puhuja 1 [00:31:15]: [Päällekkäinen puhe 00:31:15]

Puhuja 2 [00:31:15]: [Päällekkäinen puhe 00:31:15] semmonen kuntoutuspaikka vanhuksille.

Puhuja 1 [00:31:17]: Kyllä, nimenomaa, joo.

Haastattelija 1 [00:31:20]: Joo.

Puhuja 1 [00:31:20]: [?? 00:31:20] vuodeosastolle [?? 00:31:23] jatkohoitopaikaks sitte.

Puhuja 2 [00:31:26]: Aha [?? 00:31:26] no sehän on hyvä.

Puhuja 1 [00:31:26]: [Päällekkäinen puhe 00:31:26] ketkä ei pääse kotiin suoraa [?? 00:31:30]

Haastattelija 1 [00:31:35]: Onks teil, tuleeks teillä muuta mieleen, mitä en oo hoksannu vielä kysyä tai mitä haluisitte täydentää tai lisätä tai?

Haastattelija 1 [00:31:46]: Ainaki perusviestinä se, et sitä viestintää tästä [?? 00:31:48] voitas nyt parantaa ja kertoo, et missä vaiheessa on tota.

Puhuja 1 [00:31:52]: Nii.

Puhuja 2 [00:31:53]: Niin, parantaa vai pitäskö sanoo aloittaa?

Puhuja 1 [00:31:54]: [Päällekkäinen puhe 00:31:53]

Haastattelija 1 [00:31:56]: Nii.

Haastattelija 1 [00:31:57]: Niin, totta.

Puhuja 2 [00:32:00]: Ja sitte pitää liikkeellä jotenki ja käynnissä.

Haastattelija 1 [00:32:04]: Nii.

Haastattelija 1 [00:32:22]: Hyvä, mut jos teil ei oo sen enempää lisättävää, nii me oltiin nyt ihan nopeita tällä kertaa, ku meit oli, tai teit oli vaa näi vähä, nii.

Puhuja 2 [00:32:28]: Joo, paljokos täs on haastateltavia yhteensä sitte [Päällekkäinen puhe 00:32:32]

Haastattelija 1 [00:32:32]: Täs on niinku kolme ryhmää.

Puhuja 2 [00:32:34]: Okei, joo.

Haastattelija 1 [00:32:35]: [?? 00:32:35] ei tänään päässy, niin miten ne pääsee sitte, pääsiskö ne joku toinen kerta tulemaa.

Puhuja 2 [00:32:40]: Okei, just joo.

Haastattelija 1 [00:32:42]: Ja viimeksi niistä on ajatuksena tehä niinku.

Haastattelija 1 [00:32:45]: Tietysti kehitetään (…) toimintaa, mutta myös sitten kirjotetaan jonkunnäköinen tieteellinen artikkeli ja myös sitä, et yhdistetään nämä tulokset myös niihin (…) työhyvinvointitilastoihin, et onko niillä mitään yhtymää, tai katsotaan, että onko niillä mitään yhtymäkohtia siihen.

Puhuja 2 [00:33:01]: Joo, okei.

Haastattelija 1 [00:33:03]: Joo.

Puhuja 2 [00:33:05]: Eli jossain vaiheessa saadaan näistä tietoa sitten, myös tästä?

Haastattelija 1 [00:33:07]: No toivottavasti, kyllä ja toivottavasti, tota.

Haastattelija 1 [00:33:11]: Sehä on vähä niinku (…) ja (…) heiniä sitte, tai mä aattelisin sen niin.

Puhuja 2 [00:33:17]: Joo.

Puhuja 1 [00:33:17]: [?? 00:33:17] et täs on jotain ryhmiä, et mä en oo tiennyt sitä?

Haastattelija 1 [00:33:22]: Niin tai näitä, niinku tätä haastatteluryhmiä?

Puhuja 1 [00:33:24]: Nimenomaan, joo, okei.

Haastattelija 1 [00:33:24]: Joo, et viimekski se [?? 00:33:26] kolme vai neljä ryhmää viimeks ja sitten tota.

Haastattelija 1 [00:33:30]: Samal taval nyt ois ajatuksena.

Puhuja 2 [00:33:32]: Just, okei, joo.

Haastattelija 1 [00:33:36]: Mut kiitos teille, ku olitte.

Puhuja 1 [00:33:37]: Kiitos.

Haastattelija 1 [00:33:37]: Kiitos.

Haastattelija 1 [00:33:39]: Täytyy (…) vähä kysellä, että mikä [?? 00:33:40]

Haastattelija 1 [00:33:44]: Mikä se seuraava askel on, että mitä tapahtuu näille, jotka ei tullu tänään paikalla, että.

Puhuja 2 [00:33:47]: Nii, joo.

Puhuja 1 [00:33:48]: No ainaki sähköpostissa oli muistutus tästä [?? 00:33:51]

Haastattelija 1 [00:33:51]: Nii?

Puhuja 2 [00:33:51]: Okei, joo.

Puhuja 1 [00:33:53]: En tiiä, tuliko se kaikille.

Haastattelija 1 [00:33:55]: Niinpä.

Puhuja 2 [00:33:55]: Joo, (…) tuli joku päivä sitte [Päällekkäinen puhe 00:33:58]

Puhuja 1 [00:33:57]: Joo.

Haastattelija 1 [00:33:58]: Joo, nonii.

Puhuja 2 [00:34:00]: Me ollaa (…) kans naapureita, me ollaa ainoat oikeestaan tuolla röntgenin puolella, niin me, ja sit tehdään yhteistä tutkimusta tavallaa parissakin projektissa mukana.

Haastattelija 1 [00:34:09]: Nonii.

Puhuja 2 [00:34:11]: Dialogi on auki.

Haastattelija 1 [00:34:12]: Joo, hyvä, joo.

Haastattelija 1 [00:34:14]: Joo, (…) on kyllä reipas noissa [?? 00:34:15] tutkimusasioissa, et.

Puhuja 2 [00:34:16]: Hän on mahtava, joo, ja tarkka.

Haastattelija 1 [00:34:18]: Kyllä.

Puhuja 2 [00:34:18]: Hyvällä tavalla pikkutarkka, asiat ei mee ohi.

Haastattelija 1 [00:34:21]: Niinpä, hyvä nii.

Puhuja 2 [00:34:22]: Menee oikeilta [Päällekkäinen puhe 00:34:23] tutkimus [?? 00:34:26] jutut.

Haastattelija 1 [00:34:23]: Nii, kyl.

Haastattelija 1 [00:34:27]: Niinpä.

Haastattelija 1 [00:34:30]: Okei hyvä.

Haastattelija 1 [00:34:31]: Mut kiitos teille, hyvä.

Puhuja 2 [00:34:31]: Jes, kiitos.

[recording ends]

Name of recording: Haastattelu 2_2022.mp3

Length of recording: 00:59:45

Information:

[?] = Word was not understood completely but meaning is almost correct. Recording point is written to the text e.g. [word? 00:15:44]

[??] = Word could not be understood and therefore could not be written. Recording point is written to the text e.g. [?? 00:15:44]

[text] = Sounds or not transcribed parts are written with square brackets e.g. [laughs] or [interview paused due to a phone call]

[recording starts]

Haastattelija 1 [00:00:02]: Tosiaan, minkälaisia odotuksia teillä on sitä laatujärjestelmää kohtaan, miten te ootte tähän mennessä sitä oppinut ja miten te ootte osallistunut laatujärjestelmän kehittämiseen. Aloitetaanko siitä osallistumisesta, että oletteko te sen viime haastattelun jälkeen ollut sen laatujärjestelmän kanssa jotenkin tekemisissä ja onko se näkynyt teidän arjessa jotenkin vai onks se ihan täysin hautautunut kaikkien muiden kiireiden joukkoon.

Puhuja 1 [00:00:37]: Ei oo näkynyt. En oo työryhmissä ja mun mielestä työryhmät on varmasti kesken, edelleenkin. Ei niistä oo minun mielestä mitään ilmoituksia tai asioita tullut. Ainoa mitä mulle tulee mieleen, niin meidän hygieniahoitaja on tehnyt niitä ohjeistuksia uusiks, et niinkun päivittänyt niitä. Ne on meidän kotisivuilla luettavissa.

Haastattelija 2 [00:01:13]: Ihan ulkoisilla kotisivuilla?

Puhuja 1 [00:01:17]: Siis meidän talon sisäisillä, intrassa. Muusta mä en tiedä mitä on tapahtunut. Onko teillä?

Puhuja 2 [00:01:30]: (…) laittaa aina niitä sähköpostimuistutuksia että on joku JCI-kokous tai päivityskokous. Se on aina kokoukset, tai lähes... Nyt oli joku poikkeus, mutta lähes aina kokous on perjantaina, jolloin mä olen niinkun aina perjantait poissa, niin mä en ole... Meillä varmaan jos puolitoista vuotta on siitä edellisestä, niin kyllä meillä varmaan joku kokous on ollut missä mä oon ollut, mutta älä edes kysy missä ryhmässä. Jotain leikkaussalitoimintaa pohtiva ryhmä olin silloin aikoinaan. Mä en tiedä onko sitä enää olemassa.

Haastattelija 2 [00:02:11]: Mites sulla?

Puhuja 1 [00:02:14]: Ei, ei oo ollut mitään. En kuulu mihinkään semmoseen ryhmään eikä oo mitään tietoa missä mennään, et ei oo näkynyt, kuulunut mitään. Se mitä mun mielestä joskus aina on, tota... Niinkun vaan kuulee, että kyllä ne ryhmät toimii edelleen, mutta mitään tuotoksista ei oo tullut, eikä oo esitelty.

Haastattelija 2 [00:02:42]: Oisitteks te toivonut että olisitte kuullut vähän enemmän et mitä siellä tapahtuu, tai?

Puhuja 1 [00:02:48]: Kyllä mä jollain lailla varmaan. Puoltoista vuotta on aika pitkä aika. Mut tietysti koronakin on varmaan vaikeuttanut niitä tässä ajassa, mut et ehkä jonkinlaisia, että miten se laatukirja edistyy tai onko syntynyt, pitäiskö, tai haluaako joku jotain ulkopuolista arviointia niistä. Ehkä semmosta olisin odottanut.

Haastattelija 2 [00:03:22]: Entä (…), jos sä oot osallistunut ainakin yhteen kokoukseen, niin onks se ollut semmosta... Miten sä oot kokenut sen työstämisen? Onks se tuntunut mielekkäältä?

Puhuja 2 [00:03:32]: Se lähti jotenkin... Miten nyt sanois? Aivan liian monimutkaisesti liikkeelle, kun suurin piirtein tässä ois pitänyt, kun sillon aikoinaan luettiin sitä käsikirjaa miten tää pitäis tehdä, niin tuli semmonen vaikutelma että tää on amerikkalaisesta ympäristöstä tuleva systeemi jossa pitää suurin piirtein selvittää että miten toi pistorasiasta tuleva sähkö on tuotettu. Laatu alko tämmösistä asioista, niin siinä oli hirveen vaikea nähdä mitään relavanssia niillä vaadituilla asioilla meidän kannalta. Yks tai kaks kokousta oisko meillä ollut, niin siinä jotenkni me puhuttiin leikkaussalin toiminnan pullonkauloista, joista on puhuttu 30 vuotta tässä talossa ja puhutaan varman seuraavat 30 vuotta. Tunnistettiin tiettyjä ongelmia. En mä tiedä onko niitä...

Puhuja 3 [00:04:45]: Päivää.

Haastattelija 1 [00:04:47]: Päivää.

Puhuja 2 [00:04:49]: Onko niitä korjattu. Jotkut asiat on varmaan korjautunut, mut ei siitä, et en mä voi sanoo et onks se tämän...

Puhuja 3 [00:04:53]: Saanko syödä tän omenan?

Haastattelija 2 [00:04:56]: Saa syödä toki.

Puhuja 2 [00:04:56]: Onks se tämän prosessin seuraus, vai onks se muuten vaan tehtyä kehitystyötä? Mä en osaa sanoa. Jos sä kysyt mikä tän laatusysteemin nimi on, niin...

Puhuja 3 [00:05:11]: Minkä?

Puhuja 2 [00:05:11]: JCI.

Puhuja ? [00:05:12]: Missä me ollaan? Mä voin sanoa [?? 00:05:14]. En minä tiedä.

Haastattelija 2 [00:05:18]: Sä et oo ollenkaan törmännyt tähän laatujärjestelmän kehitykseen (…) ollessas?

Puhuja 3 [00:05:26]: Siis oon mä varmasti siihen joskus vuosia sitten, mutta niinkun nyt just näinä jonain hetkinä, niin ei oo ihan hetkeen tullut vastaan. Ja voin jo sanoa, että on tuolla alakerran kuntosalilla, niin voi kysyä että where is the laatu, niinkun näissä tämmösissä jutuissa, mutta ei oo tullut vastaan.

Haastattelija 2 [00:05:50]: Tää on hyvä tieto.

Puhuja 3 [00:05:54]: Vai onks teillä?

Puhuja ? [00:05:56]: Ei.

Puhuja ? [00:05:55]: Ei.

Puhuja 3 [00:05:55]: Mä aattelin et oonks mä jotenkin poikkeus, et mä oon unohtanut lukea jotain.

Puhuja ? [00:06:01]: Ei.

Puhuja 2 [00:06:02]: Et oo.

Puhuja ? [00:06:02]: Ei.

Haastattelija 2 [00:06:05]: Mutta te epäilette että ne työryhmät kuitenkin kokoontuu säännöllisesti, mutta se viesti ei vaan välity teille, vai.

Puhuja 1 [00:06:10]: Joo, mä tiedän että ne kokoontuu, et kyllä ne tekee jotain, mut se että mitä, et onko ne saanut aikaseks... Se on varmaan niinkun (…) sano tossa noin, et se oli niin... Ne lähtökohdat oli sit niin utopistisia tai semmosia, et se varmaan on vaikuttanut siihen työskentelyyn.

Haastattelija 2 [00:06:33]: No, tätä on kuitenkin kaks vuotta kohta, pitäis olla melkein tehty täällä, niin mitä te oisitte odottanut että tässä välissä olis tapahtunut? Ajatteletteko te, että olis ollut valmiimpaa nyt tähän mennessä, jos...

Puhuja 1 [00:06:47]: No, kyllä mä varmaan ajattelisin näin.

Puhuja 3 [00:06:50]: Onko siitä tullut joku, kun sä sanoit et sä tiedät?

Puhuja 1 [00:06:55]: Siis mä tiedän että (…) on käynyt kokouksessa. Mä tiedän et (…) on käynyt kokouksissa, mut mihin ryhmiin ne kuuluu - sitä mä en enää muista. Mutta tiedän, että he ovat olleet.

Puhuja 3 [00:07:07]: Ja sit sä sanoit et vaatimuk... Mitä sä sanoit äsken, että on ollut jotkut korkeet, niin mitä ne on?

Puhuja 1 [00:07:13]: Siis just se laatukäsikirja.

Haastattelija 2 [00:07:14]: JCI.

Puhuja 1 [00:07:16]: Tai ne ohjeistukset mitä annettiin, niin mä jotain niistä näin sillon ja ne oli kyllä semmosta että miten niitä lähdetään purkamaan, niin se on varmaan ollut se tosi vaikeuttava tekijä.

Puhuja 2 [00:07:35]: Sillon Villen kanssa katottiin sitä laatukäsikirjaa alussa ja Ville sano ihan oikein sen että tää on semmonen homma, että tässä täytyy olla, niinkun, jos tätä oikeesti rupee tekemään, niin tätä täytyy tehdä täyspäiväisesti, että tää ei ole semmonen sivujobi joka tehdään vasemmalla kädellä ja käydään kokouksissa, et se oli... Se olis ollut niin iso työ lähteä rakentamaan semmosta, eikä... Meille ei oikeastaan missään vaiheessa tullut semmosta ajatusta että miks tää olis tarpeen, muuta kun et (…) ruvettiin tekemään ja sit meilläkin tehdään, et me voidaan sitten kertoa jollekin että meillä on tämmönen laatujärjestelmä. Mut että kun sillä ei ollut... Ei me ainakaan nähty mikä on se välitön hyöty miks me tämä tarvitaan. Me tunnistettiin se, että meillä on monta semmosta ongelmakohtaa erilaisissa prosesseissa jotka pitäis ratkoa ja katsoa ja varmistaa että asiat menee hyvin, mut kun se laatukäsikirja oli ihan eri maailmasta, et se lähti tämmösistä täysin toiminnan kannalta epäoleellisista asioista, et onks varmaa et on juomakelpoista vettä joka tulee hanasta. Tänhän ois tietysti voinut jättää omaan arvoonsa, mutta kun se toimeksianto oli jotenkin semmonen... Ei sitä oikeastaan ollut. Tehkää laatujärjestelmä, et kokoustakaa. Ja ainut... Mulla on semmonen käsitys, että ainut ryhmä joka varmaan, tai ainut henkilö joka on saanut merkittävästi irti tästä on Jouni Lohikoski, joka on ollut siinä dataryhmässä. Jouni on penkonut [Acutea? 00:09:14] ja se on syvällä siellä, niissä tietopankeissa. Sillä on paljon tietoa ja se on varmasti sellasta mistä on hyötyä talolle, että... Et näin, tää on mun kuvitelmani tästä.

Puhuja 1 [00:09:32]: Ja sit kun heillähän on ollut tämmösiä, et heillä on tunti aikaa joskus. Varmaan, niinkun se mitä mä nyt tiedän että joku on ollut, niin varmaan ehkä maksimi kahden-kolmen kuukauden välein tunti, niin se on aika mitätöntä mitä siinä yhdessä tunnissa kerkii tekee.

Puhuja 3 [00:09:52]: No, eihän se oo... [päällekkäin puhumista 00:09:54].

Puhuja 1 [00:09:55]: Siinä keskustellaan mistä me puhuttiin viimeks.

Puhuja 2 [00:09:59]: Näin se on.

Haastattelija 1 [00:10:00]: Mikä sen laatuhässäkän... Mitä siinä on arvioitu? Onks ne prosessien arvioita tässä talossa, leikkaustoiminnan vai mitä ne on ollut mitä siinä on tehty?

Haastattelija 2 [00:10:14]: Haluaako muut vastata, mitä siinä ois pitänyt tehdä?

Puhuja 2 [00:10:19]: Mä oon ollut... En muista sen ryhmän nimeä, mut joku semmonen leikkaussalin toimintaa selvittävä ryhmä. Toisaalta mulla se menee vähän sekaisin, kun sit oli tää tämmönen, oliks se nyt se Biomedin Rapid Recovery -systeemi, joka sitten kanssa tähtäs siihen samaan, mutta se oli spesifi homma ja...

Puhuja 3 [00:10:39]: Eiks se ollu ihan hyvä?

Puhuja 2 [00:10:42]: Se oli ihan hyvä, koska sillä oli rajattu toimeksianto ja se oli handlattavissa oleva systeemi. Varmaan se sai tiettyjä asioita, parannuksia aikaan siinä. Mut sekin oli sit loppuviimeks semmonen, että siihen projektiin mä osallistuin ja siinä mä huomasin, että kyllä se niinkun semmoset ulkopuoliset sparraajat, jotka tänne tulee, niin ei me niihin voida luottaa, et kyllä ne kaverit, jotka sitä veti, niin ne näytti siltä että ei ne... Hyvä kun ne muisti että oliks tää nyt (…) missä me ollaan tällä kertaa kokouksessa, et ei se... Kyllä se niinkun itse täytyy tehdä ne projektit. Ite täytyy tunnistaa ne ongelmat. Ite täytyy löytää ratkaisut siihen. Ei... Konsultti ulkopuolelta on vähän niinkun turha juttu näissä. Ongelma on se, että näihin asioihin täytyy sit olla aikaa ja resursseja ihan oikeesti.

Puhuja 3 [00:11:47]: Saanks mä kysyä, että mikä sun rooli on tässä?

Haastattelija 2 [00:11:48]: Mun rooli on... Anteeksi, mä unohdin esittäytyä. Mä oon siis (…), mä oon tässä auttamassa tekemään tätä tutkimusta laatujärjestelmän käyttöönotosta eri ympäristössä, et on tää teidän ympäristönne.

Puhuja 3 [00:12:02]: No niin.

Haastattelija 2 [00:12:03]: Mä en tähän laatujärjestelmään sinänsä sekoittunut. Mä oon vaan kerännyt näitä aineistoja ja ollut noiden apuna sen tutkimus... Teidän toi Anni Vuohijoki on siinä, tekee väitöskirjaa tästä aiheesta ja sitten hänen kanssaan tätä on tehty.

Puhuja 3 [00:12:23]: Okei.

Haastattelija 2 [00:12:25]: Ja mulla on ollut enemmän tää laadullinen osuus, että (…) on katsonut niitä kvanttiaineistoja ja selvittänyt sitä yhteyttä työhyvinvointiin. Mut tää on jotenkin edennyt nyt selkeesti hitaammin kuin alunperin ajateltiin ja nyt tässä kerätään silleen, että ihan alussa tehtiin tämmöset haastattelut ja nyt tehdään nää haastattelut ja nyt varmaan kerran tai kaks vielä myöhemmin.

Puhuja 3 [00:12:46]: Ja pitäiskö tää laatujuttu näkyä jotenkin meidän, et tää kehittäis meidän omaa työhyvinvointia?

Haastattelija 2 [00:12:50]: Se ois hyvä, mutta eihän se, jos se ei oo kelläkään edes tiedossa eikä oikeestaan mitään seuraavia askeleita oo otettu, niin eihän siitä oo sitten mitään näyttöä, ainakaan vielä.

Puhuja 3 [00:13:02]: Niinpä. No joo. No, ei ainakaan meikäläiselle.

Haastattelija 2 [00:13:08]: Niin.

Puhuja 1 [00:13:09]: Mut sen mä tiedän että niillä osassa työryhmissä niillä oli se, että näitä ohjeistuksia ne rupes käymään läpi ja katsomaan et mitkä on vanhoja ja miten niitä edes tähän päivään sijoitettuja, tai sitten että ne olis nykypäivää vastaavia ja sitten sieltä paljon... Mä tiedän sen verran, että paljon putsattiin pois vanhaa ja niitä kautta, tai niitä on rakennettu, mutta miten sit ne nivotaan tähän kokonaisuuteen, niin sitä mä en tiedä.

Haastattelija 2 [00:13:41]: Ja kun kysyit mitä tän laatujärjestelmän pitäis olla, niin monesti se on just niitä prosessikuvauksia, et kaikki tietää mitä tapahtuu missä vaiheessa. Just ajantasaisesta ohjeistuksesta ihmiset tietää että mikä löytyy mihinkin asiaan, mikä ohjeistus, et osaa toimia erilaisissa tilanteissa niinkun on sovittu. Se on yleisesti näitä laatujärjestelmän tämmösiä perus... Mut se...

Puhuja 3 [00:14:01]: Mä muistan, että tää on tehty siis joskus aikoinaan kun Sarkkisen Kristiina oli, kun oli meillä Kelan kuntoutuksia, niin niihinhän tehtiin. Meillä on joku hemmetinmoinen laatukalent... Tai toi mappi, niin siinä oli justiin ja varmaan sillon oli näitä että mitä milloinkin kuullut, mutta sen jälkeen kyllä tää on ihan mulle uutta.

Puhuja 1 [00:14:23]: Mut ne on niin pienissä osissa vielä mitä tehdään.

Haastattelija 2 [00:14:29]: Mut sanoisitteks te että tää laatujärjestelmä on vielä yhtä alkutekijöissään kun se oli sillon puolitoista vuotta sittenkin, vai? Niinkun sä sanoit että jotain ohjeistuksia päivitetty.

Puhuja 1 [00:14:38]: Mä sanoisin et se varmaan on isossa mittakaavassa, niin nää on niinkun ihan tämmöstä pientä, anteeks, räpellystä mitä on tehty.

Puhuja 3 [00:14:47]: Niin.

Puhuja 1 [00:14:48]: Et miten ne sitten niinkun nivoutuu ja mitä sitten niissä ryhmissä, ketkä kuuluu näihin jotka on tehnyt niitä, niin mitä siellä sit tapahtuu, niin se on niinkun täysin pimennossa.

Puhuja 2 [00:15:03]: Joo, sama vaikutelma tulee. Joku ohjeiden päivittämistyö, sehän pitäis periaatteessa olla että jos ohjeita tehdään, niin senhän pitäis olla automaatio, eikä niinkun laatuprojektin [aikana? 00:15:14] päivitetään ohjeet.

Puhuja ? [00:15:14]: Joo.

Puhuja 2 [00:15:18]: Et sitä nyt ei voi tämmösenä saavutuksena mainostaa. Mut tosiaan, niin näiden... Varmaan on fokusoituja hyötyjä joissain tietyissä alueissa. Mä luulen et se datankeruu on semmonen mistä on varmaan ihan oikeesti hyötyä.

Haastattelija 2 [00:15:37]: Ja mitä dataa hän siis keräs?

Puhuja 2 [00:15:40]: Kaikkea mitä tuolta meidän potilastietojärjestelmästä löytyy, et siellä on erilaisia leikkaussalin aikaleimoja joita sinne kirjataan, mutta ei niitä kukaan ole välttämättä osannut ajatella että mitä niillä tehdään. Mut että tää on semmonen nyt, että siitä ei varmaan oikein tiedä kuin Jouni. Ehkä Mikko varmaan hyötyy siitä tiedosta, mutta...

Haastattelija 2 [00:16:09]: Kun se varmana käytetään myös sitä, kun alunperinhän tavoitteena on myös että jos tämmösen avulla saatais esimerkiks leikkausaikoja lyhennettyä tai jotenkin järkevöitettyä ettei mee mihinkään turhaan sähläämiseen siinä välissä aikaa, et sen takia ne varmaan kattoo niitä leikkausaikaleimoja.

Puhuja 2 [00:16:27]: Joo.

Haastattelija 2 [00:16:30]: Vai miltä se näyttää leikkaussalin toiminnan kannalta?

Puhuja 2 [00:16:35]: Siis sillä tavalla se näkyy, että jos mulla oli semmonen käytännön kysymys et mun piti hakee, kuinka paljon meillä on leikkauksia peruuntunut, niin se on hirveen vaikee lähteä etsimään mistään, mutta sit kun meni Jounille niin se hetken aikaa näpytteli konetta ja sillä oli siinä todennäköisesti kohtuullisen kattava listaus. Et sillä on niinkun tämmönen tatsi siihen potilastietojärjestelmään ja mitä sieltä ylipäätänsä löytää. Et siinä on hyviä työkaluja varmasti kehittää tätä, mut jotenkin musta tuntuu et se ainakaan mulle ei oo näkynyt, et mitä sillä nyt on tehty, että... Ja taas nää lopputulokset eli prosessien sujuvuus, niin ehkä ne muuttuu niin hitaasti että sitä ei vaan huomaa, mutta musta tuntuu että ei ne oo paljon muuttunut tässä. Mutta sit jos aattelis, jos vois mennä 10–15 vuotta taaksepäin, niin ehkä sit tajuis että onhan ne varmaan aika paljon muuttunut. Meidän hoitoajat vuodeosastolla on lyhentynyt tasaisesti ja monet käytännöt on muuttunut pikkuhiljaa, et kyllä se asiat muuttuu, mutta et onko se sitten... Onks se laatuprojektin aikaansaannosta, vai onks se sitä että täällä on ulkopuolisia toimijoita jotka tuo sitten uusien, toisten talojen toimintatapoja, kulttuuria? Täällä on ollut paljon(…) toimintaa, et sieltä on tullut tiettyjä ehkä tämmösiä toimintakulttuurin muutoksia sieltäkin.

Puhuja 3 [00:18:21]: Ja sitten tietenkin tulee mieleen aikaa, et mennään ajassa eteenpäin, seurataan perässä, vaikka me ollaan sitten vielä ehkä vähän niinkun jälkijunassa joissakin asioissa, mutta että... Niin.

Puhuja 2 [00:18:38]: Ja sit jotkut asiat menee myös huonommiksi, niinkun Kelan suorakorvauksen menetys esimerkiks, että aika tuo hyviä ja aika tuo huonoja muutoksia.

Haastattelija 2 [00:18:53]: Mut aika vähän selkeestikään näillä muutoksilla on mitään tekemistä tän laatujärjestelmän käyttöönoton kanssa.

Puhuja 2 [00:18:57]: En mä voi oikeestaan nähdä yhteyttä mitenkään.

Puhuja 3 [00:19:00]: Ja sit vielä se, että se vaikuttais meidän työhyvinvointiin.

Puhuja 1 [00:19:04]: Niin, tai työturvallisuuteen tai työmotivaatioon.

Puhuja 3 [00:19:07]: Niin, tai työympäristöön.

Puhuja 1 [00:19:09]: Niin.

Puhuja 3 [00:19:10]: Enemmänkin tässä nyt voitais miettiä tällai meidän, että mikä vaikutus näillä on tällai, et meillä on tämmönen Ukrainan sotapommijäljitelmä täällä niinkun, et mikä näilläkin on ollut, että asiakkaisiin se on ainakin vaikuttanut. Se ei nyt tähän, mutta se että asiakkaat kommentoi tätä härdelliä mikä tän talon ympärillä, ympäristössä ja kaikkee muuta, niin se on se mikä tässä nyt on eniten puhuttanut, niinkun jos se vaikuttaa laatuun. Se ei välttämättä vaikuta työn laatuun, mutta se vaikuttaa tämmöseen asiakasviihtyvyyteen ehkä, vaikka se ei oo meistä millään tavalla riippuvainen. Mut ne on ehkiä semmosia asioita mitkä puhuttaa tässä enemmän.

Haastattelija 2 [00:19:56]: Jos miettii sitä työhyvinvointia, niin näätteks te mitään yhtymäkohtia millä laatujärjestelmä vois vaikuttaa siihen työhyvinvointiin?

Puhuja 2 [00:20:06]: Mä luulen, tai se miten tuntuu et se yhteys oli siinä, että jos kaikki tietää miten hommat tehdään ja kaikki tekee ne samalla tavalla, niin se vähentää semmosta turhaa työtä tietenkin, niin sitten se vähentää myös niitä ihan oikeita, mahdollisia haittatilanteita ja haittatapahtumiakin. Ja sillon jos toimii ympäristössä jossa voi luottaa siihen että asiat menee niinkun on sovittu, musta se on työhyvinvointia lisäävä tekijä sillon.

Puhuja 3 [00:20:43]: Kyllä.

Puhuja 2 [00:20:45]: Et kyllä sillä on yhteyttä.

Haastattelija 2 [00:20:50]: Mitä te ajattelette, että mikä on tällä hetkellä teidän työhyvinvoinnin semmonen kantava voima täällä?

Puhuja 3 [00:21:01]: No siis, miks mä oon täällä töissä ja miks mä oon täällä näin kauan viihtynyt, niin näköjään ne on ne ihmiset täällä ja se välitön, niinkun että (…) nään tuolla niin mä sanon (…) auta, mitä mä teen sen asiakkaan... Ja (…) auttaa mua. Nää... Mä meen... Mä oisin [?? 00:21:17], niinkun et nää ihmiset ja sitten niinkun et mä meen hänelle sanoo tonne et hei, et yes, mä oon ihan pulassa, auta tän asian kanssa mikä härdelli täällä, [?? 00:21:27], niin se on se mun työ. Niinkun (…) sano äsken, et ihmiset hoitaa omat tonttinsa ja jokainen on valmis auttamaan. Ja mun mielestä se on se välitön mikä luo mulle työhyvinvointia ja semmosta. Ja mun mielestä se näkyy myös valtavasti siinä et me välitetään meidän asiakkaista. Ja se on sitä, et kun asiakas tulee, kaikki on mun mielestä valmiita, et hei, mihin sä oot menossa, vaikka se on välillä kun tää (…) -härdelli on tässä ja

yks on menossa silmään ja toinen [?? 00:21:56]. Ei tiedä ikinä mihin ne menee. Niin, silti mun mielestä pääsääntöisesti ihmiset, me ollaan... Ja se on se miks mä ainakin oon ollut täällä ja se on varmasti työhyvinvoinnin kannalta, jos nyt mietitään leikkaussalia, kaikki nää härdellit, mutta sen työhyvinvoinnin kannalta, et millä tavalla me huolehditaan niistä ihmisistä.

Puhuja 1 [00:22:16]: Ja toisistamme.

Puhuja 3 [00:22:18]: Niin, toisistamme.

Puhuja 1 [00:22:20]: Mistä apua saa ja...

Puhuja 3 [00:22:22]: Niin.

Puhuja 1 [00:22:23]: Et helppo toimia.

Puhuja 3 [00:22:25]: Helppo toimia. Välitön tunnelma. Ei oo sellasta hierarkia-asetelmaa, etteikö uskaltais kysyä toiselta mitään, riippumatta siitä mikä on hänen tittelinsä tässä talossa. Ja se kun säilyy, niin mun mielestä sillon homma myös sujuu. Semmonen niinkun tiimityöskentely ja se, se on sellasta mihin mä oon tottunut täällä talossa ja se on se kantava voima. Se on mun mielestä työhyvinvointia aika isossa määrin.

Puhuja 2 [00:22:54]: Se oli oikein hyvin sanottu. Siihen ei oo muuta lisättävää, kuin että sitä työhyvinvointia on vaikea edes laatujärjestelmän rikkoa.

Puhuja 3 [00:23:03]: Näin on.

Haastattelija 2 [00:23:03]: Kyllä.

Puhuja 3 [00:23:04]: Mä juttelin tässä viime viikolla siis (…) kanssa ja (…) kans sano että vuosia kun hän on täällä ollut, hän olisi varmaan palkkaa saanut muista taloista paljon enemmän, mutta niinkun todettiin yhteen ääneen että se raha ei ole se työmotivaation tärkein tekijä, vaan kyllä se on se missä ympäristössä me tehdään töitä ja minkälainen on se työyhteisö. Sillä on ihan käsittämätön merkitys. Ja tää talo on ollut vuosia siitä, että kun

munkin ystäviä ketkä on ollut täällä töissä aikasemmin ja nyt on muualla, niin kyllä se me-henki on se mikä sinne sydämeen on juurtunut, joka on semmonen ainakin mun ammatti-identiteetin isoin juttu. Mun ystävät ketkä ei oo ollut töissä missä on tämmönen moniammatillinen juttu ja muut, niin ei he, kun he on ehkä tehnyt pienissä yksityistöissä, niin ei siellä oo, niinkun... Ne on jäänyt isosta jutusta paitsi, minun mielestä. Varmaan te yhdytte ihan samaan. Et sit me voidaan miettiä työhyvinvoinnin kannalta mitä on ehkä puitteet missä me työskennellään ja kyllä tällä hetkellä tää missä minäkin työskentelen, niin ne näkyy... Raha on tiukassa. Kaikki tämmöset välineet millä me tehdään on niinkun ihan osa niinkun morjens - näkemiin. Ja sitten ympäristö, et tuolla on niinkun seinä pois, siinä on ulkoseinä vastassa, näkyy taivas ja sieltä tulee pöly sisään, niin ne on niinkun laatutyöskentely... Se on, niinkun mitataan laatua ja työhyvinvointia, niin siinä me mennään tällä hetkellä niinkun alta todellisen riman. Nää on mitä mulle tulee tässä nyt mieleen.

Haastattelija 2 [00:24:53]: Haluaako muut täydentää?

Puhuja 1 [00:24:57]: Tossa oli mun mielestä aika hyvin sanottu. Näinhän se on, että se on mikä auttaa jaksamaan sinä. Et pienen talon etu, mitä ei koskaan ollut isossa talossa missä mäkin oon ollut töissä. Se oli todella hankalaa saada asioita hoidettua ja hyvin byrokraattista ja näin, et se on se tää meidän puoli, että... Ja se on varmaan se mikä näkyy kyllä potilaisiin meistä.

Haastattelija 2 [00:25:27]: Miten te nyt aattelette tän laatujärjestelmän, kun tosta puheesta mä en saanut millään tavalla kiinni siihen laatujärjestelmään?

Puhuja 3 [00:25:33]: Joo, sen takia mä en osaa yhtään [päällekkäin puhumista 00:25:36].

Haastattelija 2 [00:25:37]: Ajatteletteko te, että olisko siinä mit... Et se, että ihmiset jokainen tietää mitä ne tekee, niin se on tietysti osa sitä laatujärjestelmää, et ne ohjeistukset on jotenkin ajantasalla, että jokainen, niinkun... Et jos ei tiedäkään mitä tekee, niin sit on joku mihin voi turvata, et joku on pitänyt huolta siitä. Aina joku on pitänyt huolta siitä, että ne ohjeistukset, et niihin voi luottaa mitä annetaan. Mutta mikä muu yhteys, jos ajatellaan että tällä laatujärjestelmällä vois olla tähän työhyvinvointiin tai tämmöseen? Kun mä mietin kun puhuitte tosta (…) olemisesta tai remontista, niin sehän nyt ei liity tähän kauheen paljon.

Puhuja 3 [00:26:16]: Mutta eihän siinä, niinkun siinä ohjeistuksissa ja sun muuta, kun miettii sitä, niin eihän täällä edelleenkään oo mitään semmosta että ihmiset tietäis että millä tavalla me toimitaan jossakin tilanteessa, et meillä ei oo semmosta yhtenäistä, niinkun (…) just sano. Eihän meillä oo mitään siis semmosta. Meille ei tuu mistään korkeammalta

taholta, niinkun täältä näin alakertaan työntekijöillä, lääkäreillä, kellä vaan, mitään semmosta että hei, että tämä asia hoidetaan näin, näin, näin. Et ei täällä oo mitään semmosta. Ei täällä oo ikinä ollutkaan, vaan me ite työntekijät itse asiassa sovelletaan jotain täällä. Me toimitaan niinkun parhaaksi nähdään, suurin piirtein.

Haastattelija 2 [00:27:00]: Mut pitäiskö sun mielestä olla, vai olisko se, niinkun...

Puhuja 3 [00:27:02]: Se olis työntekijöille... Mun mielestä se olis helpompi hengittää, kun sun ei tarvii puuttua semmoseen asiaan mikä ei todellakaan kuulu ehkä mulle. Se olis ihan vaikka jonkun toimitusjohtajan asia, ihan oikeesti, et ei mun tarviis päättää tämmösistä asioista. Ainakin me työntekijöinä ollaan siinä ihan siis työntekijöitä ja ihan siinä ensimmäisessä kontaktissa niiden potilaiden kans, niin kyllä se olis hyvä että siinä olis välillä semmonen ihan oikeesti, kun itekin oon ollut isossa talossa töissä ja tiedän että sieltä tuli vaan... Ja kaikki oli, niinkun vähän armeijatoiminta, mut tosi hyvin homma toimi. Siitä ei luistettu mihinkään suuntaan, et se vaan oli niinkun näin. Et totta kai, tää on niin pieni paikka ja täällähän yritetään aina parhaansa mukaan palvella meidän potilaita, et sehän on se ykkösasia ja nehän saakin [siitä? 00:28:04] sitten, mutta joskus on semmosta että jos olis se ohjeistus jostakin että millä tavalla me toimitaan jossakin tietyssä asiassa, niin mä voin olla ihan varma että se asiakaspalvelu ehkä olis vähän parempaa ja se helpottais kaikkea työtäkin, ettei olis siitä sitten stressiä, että... Että, tota... Siinä on monia asioita, mitä... Tää on vaan tää talo, että...

Puhuja 2 [00:28:33]: Tunnistan ton ajatuksen ja sitä alleviivaa minusta se, että meillä on aika paljon myös vaihtuvuutta. Siis meille on hirveen paljon uusia ihmisiä tullut töihin.

Puhuja 3 [00:28:46]: Nyt jo.

Puhuja 2 [00:28:47]: Polilla näkyy, ajanvarauksessa näkyy, leikkurissa näkyy, osastolla näkyy. Siinä olis ehkä semmonen, uuden ihmisen perehdytyksessä. Varmasti sitä on mietitty ja tehty perehdytyskansioita, mutta mä luulen että just toi että sulla olis hyvin, hyvin selkeä ohjelma. Ja sitten toisaalta siinä täytyy myös sitten olla se, hyväksyä se että kun siinä joku istuu vieressä, niin vaikka on kaks ihmistä töissä, niin siinä ei oo kahden ihmisen työpanosta vaan se on rahan menoa se uuden ihmisen kouluttaminen jotta siitä saadaan kunnon työntekijä. Mut tää on se... Ja niinkun Anu sano, niin tää niukkuuden kanssa eläminen on semmosta joka liian paljon haittaa tässä. Se antaa semmosia reunaehtoja nykyään, että ei oikein... Ei jaksa haaveilla.

Puhuja 3 [00:29:57]: Niin ja sitten niinkun laatuun tulee mieleen sekin, et kun ihminen tulee taloon niin minkälaista kuvaa... Meillähän tosi paljon on uusia potilaita ihan mielettömästi tullut nyt, varsinkin tän koronan aikana, niin minkälaisen kuvan ne ihmiset saa tästä koko jutusta. Kun ne tulee sisään näe ettii että missä me ollaan, niin mihin ne tulee ilmottautumaan. Me ollaan siellä jossakin nurkassa, suurin piirtein näin pöydän takana istumassa, kun meillä ei oo kunnon työpisteitäkään siinä. Et me ollaan kuitenkin ortopedinen sairaala, että, tota... Et kyllä välillä oikeesti hävettää, kun meillä on [lihalaatikon? 00:30:41] päällä printterit sun muut siinä ja sit tila on se, kun meet istuu sinne ja katot sitä koko tilaa, niin se on tosi sekava. Mut se on just, että kun ei sitten oo rahaa sitten tämmösiin, tai...

Puhuja 2 [00:31:01]: Eikä se... Mä oon vähän sitä mieltä, että tässä on paljon semmosta... Ihmiset ei oo... Päättävissä asemissa olevat ihmiset eivät oo sisäistäneet mitä se laatu tarkoittaa. Katsokaapa joskus... Kiertäkää talossa ja katsokaa meidän kylttejä ja opasteita. Ne on nyt pikkuhiljaa parantunut. Kattokaa meidän tuolla polin käytävällä ovien saranapieliä, [päällekkäin puhumista 00:31:30].

Puhuja 3 [00:31:29]: Mä katoin äsken. Joo.

Puhuja 2 [00:31:33]: Tuolit on nuhjuisia. Kaikkea sellasta.

Puhuja 1 [00:31:32]: Kaikkia tämmösiä, joo.

Puhuja 3 [00:31:36]: Seinät on rikki ja...

Puhuja 2 [00:31:39]: Mun mielestä sen pitäisi ottaa silmään sen verran että joku tajuaa että hyvä tavaton, täähän täytyy korjata. Tää täytyy pestä tää tuoli. Meillä ei oo sellasia ihmisiä. Meillä siivoojat kulkee ja osa huomaa ja osa ei huomaa. Ja sitten taas meidän markkinoinnista ja imagosta kiinnostuneet ihmiset ei tunnu tietävän yhtään miltä meillä näyttää. Että...

Puhuja 3 [00:32:10]: Ja toi on siis ihan... Mä tein tämmöstä laadullista vai määrällistä... Laadullista tutkimista. Kumpi se nyt on? Mä pudotin roskan tonne meidän yhteen kierreportaisiin ja mä seurasin et kauan se roska siinä on. Se oli siinä viikkotolkulla, joten sit mä hermostuin ja selvitin kuka tän siivoo. Ja kun täällä talossa on kolme eri siivoushässäkkää, kuka siivoo ton ja sit seuraava käytävä ei ookaan enää sen, vaan se on toisen. Sit selvis, että kukaan ei siivoa sitä kierreportaita ja se roska oli siinä ja muuta. Eli tää

on just sitä, että semmoseen, niinkun... Mulla on semmonen tunne, että jos ei kohta tapahdu jotain, tiiäks sä sellasta että mä vähän ravistan tästä näin ja katotaan et miten... Mä ihan tietoisesti tulin tänään, kävelin yhtä penkkiriviä, sitten joka ikinen penkki oli erivärinen. Ja sulla on tiedäts sä jotain, vuodelta -97 kun mä oon ollut täällä, niin jotain semmosia kangastauluja tuolla seinällä. What the fuck? Niinkun tiedäts sä, se on mun mielestä laatua että se näyttää laadulliselta, koska se mitä me täällä tehdään, se on ihan OK ja laadukasta, mut sen pitäis se ulkonäkö... Se asiakas tulee, niin sille tulee semmonen tunne että ihana, täällä on freesiä. Ja se ei kyllä välity ihan kaikkialta ja se on sitä... Mun mielestä sekin on laatuprosessi, jossa voitaisiin selvittää... Se ei varmaan näy tässä, mutta miten tämä tällainen tämän talon pirstaleisuus moneen eri juttuun näkyy niiden toimintojen, niinkun, et miten ne toiminnot on hajautettu monille toimijoille ja sit yhtäkkiä huomataan että ei kukaan pitänyt tästä asiasta huolta. Ja esimerkiks omassa ammatissa, fysioterapia, niin toi välineistö ja kaikki muu on, niinkun... Ja jos laatua on koulutus, niin meidän fysioterapian koulutus laahaa niinkun way back tuolla. Se on... Kukaan... Mä oon itse tässä nyt ammatinharjoittaja, mä oon pari vuotta sitten sanonut palkkatyöstä itseni irti ja tullut tähän taloon ammatinharjoittajaks, niin raha on niin tiukassa että koulutukset on ihan... Niinkun se on surullista. Ja se, et me hankitaan tonne joku satasen joku juttu, niin siitä kysytään että saadaanks tämmönen hankkia. Mitä? Niin, se vaan jossain vaiheessa se tulee jollain tavalla... Ihmisellä on vara valita meneekö se pölyiseen kuntosaliin vai meneekö se jonnekin missä on kahviautomaatti ja vesiautomaatti mistä saat valita - ahaa, mulla on kahviautomaatti. Vai ottaaks se rätin ja pyyhkii ensin pölyt pois? No, hei. Et tää on... Se ei varmaan näy näissä prosesseissa, mutta se on mun mielestä sitä työhyvinvointia sitä isommassa määrin.

Haastattelija 2 [00:35:10]: Onko toi taloudellinen niukkuus nyt uus ilmiö, vai onko se ollut pitkään?

Puhuja 3 [00:35:11]: Kyllä se on ainakin ollut [pidempään? 00:35:14]. Niin.

Puhuja 1 [00:35:11]: On se ollu.

Puhuja 3 [00:35:26]: Ja semmonen... Sitä ohutta ilmottautumistakin, sillon kun me muutettiin siihen... Kauanko me ollaan oltu? Neljä vuotta?

Puhuja 1 [00:35:26]: Mä oon ollut pitkään.

Puhuja 3 [00:35:29]: Neljä, viis vuotta. (…) jäi sillon eläkkeelle. Varmaan viitisen vuotta kohta, joo. Ne luvattiin... Sillonhan meille luvattiin, että tehdään remontti. Ihan täys remontti. Alkakaa suunnitella. Kesällä kun on sulkuaika, tehdään remontti. Tultiin kuule, ei

ollut mitään eikä tähänkään mennessä oo mitään eikä me saada edes kunnon työpisteitä sinne. Että ihmiset hakkaa päätään siihen pleksiin ja harva se päivä ja sit kun se tiski on niin lyhyt ja sit kun se pleksi on siinä, niin kaikki on siinä päät kiinni ja me muut pyyhitään koko ajan sitä. Se pitäis olla se [auto? 00:36:02] semmonen. [Mä sit pyyhin? 00:36:04] aina [?? 00:36:06]. Ihan surkeet on työpisteet, plus se et kun ihminen tulee ihan oikeesti, et millä tavalla... Mihin se kiinnittää huomioo?

Haastattelija 2 [00:36:13]: Ootteks te antanu näistä epäkohdista palautetta teidän esimiehiltä täällä talossa?

Puhuja 1 [00:36:21]: Kyllä niitä on.

Puhuja 3 [00:36:23]: Niitä on tosiaan.

Puhuja 1 [00:36:24]: Remonttitoiveita on esitetty hyvin usein, hyvin paljon. Ja sanottu, et tarvitaan uudet tuolit potilaille, et näyttäis edes vähän paremmalta.

Puhuja 3 [00:36:34]: Itellekin tarviis uudet työtuolit, kun pukki on työtuoli missä me istutaan. Mä oon joskus aikoinaan mennyt ihan edellisen toimitusjohtajan puheille hermostuksissaan ja... Nyt oon pikkusen laantunut siitä, vähän ottanut... Ajatellut että no... Mut joo. Kyl se mua ainakin, niinkun... Kyl mä oon siitä sanonut ja semmosena, niinkun tällä viikollakin, et tää näyttäytyy tämmösenä, niinkun... Jos... Niinkun mulla tulee se, että jos mä olisin vastuussa tietyistä jutuista, että toi on niinkun likainen, noi jutut, niin mähän olisin siellä kun helvetin haukka, sanomassa että nää korjataan heti. Mä en tiedä onko... Mitä niiden eteen tehdään, mut semmonen, niinkun... En mä tiedä. Onko ne kilttejä? Eiks ne uskalla räjähtää? Tai ei tartte räjähtääkään, vai eiks sillä oo, niinkun...

Puhuja 2 [00:37:39]: Tää on just se, että laatu kasvaa tämmösistä. Laatu syntyy pienistä asioista ja niistä välittämisestä. Ja sillon jos useista pienistä asioista välitetään se kokonaisuus kasvaa. Mut sä et saa laatua tehtyä niin et se jotenkin tiputetaan täältä ja oletetaan et se tunkeutuu joka paikkaan.

Puhuja 3 [00:38:01]: Niin.

Puhuja 2 [00:38:02]: Se on varmaan tän tämmösen laatujärjestelmän käyttöönoton, voisin kuvitella et se on aika tämmönen universaali vaikeus, et se tarvii ensin jollakin tavalla kokea

että tässä on jotain mitä pitää parantaa ja et se on nyt niinkun meidän tehtävä parantaa. Tässä on se paradoksi sitten, että mä ajattelen että jos (…) saadaan tää laatujärjestelmä käyttöön, niin mieti minkälaiset otsikot siitä revitään ja miten sitä mainostetaan. Siitä huolimatta ne ihmiset jotka tänne tulee samaan sit hoitoa kun ne ei saa sitä (…), niin ei se niiden kokemus siitä laadusta, ei se oo yhtään sen parempi.

Haastattelija 2 [00:38:42]: Niin, et jos ne ei saa sitä hoitoa. Niin.

Puhuja 2 [00:38:43]: Niin, et se tavallaan, niinkun vaikka sulla olis kuinka hienosti rummutettu laatujärjestelmä ja sertifioitu, niin jos ei se vaan tuu, niin se on ihan turha homma. Että miten se nyt olis? Puhutaan sydämen sivistyksestä. Täytyis olla sydämen laatua, josta tää homma lähtee.

Puhuja 1 [00:39:07]: Kyllä.

Haastattelija 2 [00:39:07]: Niin ja semmonen joku yhteen... Kun kuuntelen sua, niin [?? 00:39:12] yhteisvastuullisista asioista, et jos nähdään että joku repsottaa nyt oikeesti, niin et niinkun jokainen antaa, rupee niinkun kiinnit... Tai vie sitä asiaa eteenpäin, et jotenkin et miten niihin voi sitten puuttua. Mutta jos ei oo taloudellisia resursseja, ei korjata mitään asioita, niin sit se on, niinkun...

Puhuja 3 [00:39:26]: Niin, tai semmosena se ainakin näyttäytyy.

Haastattelija 2 [00:39:31]: Niin.

Puhuja 2 [00:39:33]: Mut se niinkun mindset, se ei oo kiinni taloudesta.

Puhuja 3 [00:39:36]: Ei.

Puhuja 2 [00:39:37]: Vaikka olis kuinka nuukaa ja niukkaa, niin aina voi pikkusen korjata ja laittaa nätimmäks.

Puhuja 3 [00:39:43]: Kyllä. Se lupaa paljon, kun sä tuut tohon pääaulaan.

Puhuja 1 [00:39:48]: Pääaula on hieno.

Puhuja 3 [00:39:50]: Se lupaa, mut sitten...

Puhuja 1 [00:39:51]: Lähet eteenpäin, niin...

Puhuja 3 [00:39:55]: Me lähetään eteenpäin, niin sithän se onkin vähän toinen juttu.

Puhuja 2 [00:39:57]: Missä vaiheessa se muuttuu Petroskoin keskussairaalaksi?

Puhuja 3 [00:40:02]: Niin.

Puhuja 2 [00:40:03]: Pääaulan jälkeen se muuttuu aika nopeesti.

Puhuja 1 [00:40:05]: Kyllä.

Puhuja 3 [00:40:06]: Mehän pyydettin sitä pääaulassa, se lasikoppi mistä muutti pois se liike, että me saatais ilmottautuminen kato siihen, niin sinnehän olis voinut laittaa ajanvarauksen kun olis väliseinän rakentanut ja siellä on vessa ja kaikki, kuule yhteiset, hienot tilat kun tullaan sisälle ainakin ja helppo ohjata ihmisiä siitä.

Puhuja 1 [00:40:26]: Kyllä.

Puhuja 3 [00:40:27]: Kukaan ei oo tehnyt yhtään mitään. Sanottiin vaan, et se on jo vuokrattu. Ja siitä nyt on... Kauanko se on seissyt tyhjänä? Puolitoista vuotta kohta. Vuosi ainakin.

Puhuja 1 [00:40:38]: Joo.

Puhuja 3 [00:40:37]: Ei oo tapahtunut eikä [me saada? 00:40:40].

Puhuja 1 [00:40:41]: Ei.

Puhuja 3 [00:40:42]: Liian kallis. Mä oon hoitanut noista useita ja jokaisen työterveyshuollon lähetteeseen mä oon kirjoittanut, että puutteellinen ergonomia, ehdottomasti tehtävä jotain. Ei siihen kukaan puutu ikinä varmaan, vaikka kuinka moneen juttuun laittanut. Ne... Niinkun edelleen mä oons itä mieltä, että laatuhyvinvointi lähtee ihmisten... Se lähtee ympäristöllä, ihan ilman... Meillä on ihanat ihmiset täällä ympärillä ja mun mielestä se semmonen, tämmönen toiminta toimii. Että varmaan sitten just tämmöset ehkä jotkut, niinkun että on selkeet sävelet et miten joku homma toimii... Mä uskoisin, että eiks varmaan se [?? 00:41:24], se et kun ihminen tulee leikkaukseen, niin eiks se polku oo aika selkee.

Puhuja 2 [00:41:31]: Se on aika selkee kyllä ja siinä on saatu paljon selkiytettyä, et se on kyllä. Se on myönnettävä. Mut se ei oo tän JCI:n ansiota, vaan se on ihan et se on ollut erillinen toimeksianto ja projekti.

Haastattelija 2 [00:41:48]: Mitä siinä konkreettisesti siis tehtiin?

Puhuja 2 [00:41:51]: Tavallaan katottiin se leikkauspotilaan polku, se prosessikuvaus, että mitä tässä tapahtuu ja mitä pitäis tapahtua ja miten se menee. Ja sitähän nyt on sit jollain tavalla jopa seurattukin. Musta se on mennyt [päällekkäin puhumista 00:42:04].

Haastattelija 2 [00:42:02]: Ja onhan sekin semmonen laatuprosessi sitten, että katotaan se.

Puhuja 2 [00:42:08]: On, nimenomaan.

Haastattelija 2 [00:42:10]: Kyllä. Miten te muuten näette, kun puhuitte paljon työhyvinvoinnista teidän kannalta, mut entäs mites yhtymäkohta potilasturvallisuuteen ja potilaiden näkökulmasta tää laatujärjestelmä? Miten tää vois näkyä teidän mielestä enemmän, muuten kun seiniä maalaamalla ja vähän freesaamalla tätä, mutta, että...

Puhuja 2 [00:42:30]: [?? 00:42:31] viimesijaisia tarkoituksia sille, että laadukkaassa toiminnassa ei asioita unohdu eikä tehdä virheitä niin paljon, että... Se on ilman muuta se tarkotus, mut sillon nekin asiat oikeastaan on tää rapid recovery, leikkauspotilaan polku, se on nimenomaan semmonen joka vähentää näitä ongelmatilanteita, mut että se pitäis

varmaan tehdä... Meillä on toinen sellanen potilaspolku. Suuren yrittämisen ja ponnistuksen jälkeen me saatiin puudutuspotilaiden polku toimimaan jotenkin vähän selkeämmin, hermojuuri- ja selkäpuudutuksiin tulevat potilaat. Niillä on nyt semmonen tietty kaava, jonka mukaan toimitaan ja musta se on selkeyttänyt asioita.

Puhuja 3 [00:43:26]: Joo, on.

Puhuja 2 [00:43:28]: Kyllä, mut et nääkin on sellasia että nää täytyis melkein aina ottaa yksi kehityskohde aina kerrallaan ja tehdä se. Sitten jos näitä polkuja on, semmosia polkuja, jotka aktiivisesti on ylläpidettyjä, niin jos niitä alkaa olla useita, niin sillon tavallaan vois ehkä niputtaa niitä jonkun tämmösen isomman laatusateenvarjon alle. Musta sensuuntainen liikenne menis jotenkin luontevasti.

Haastattelija 2 [00:43:59]: Kyllä, joo.

Puhuja 1 [00:44:02]: Onhan meillä näitä nyt, kuule... Ne ei oo varmaan nyt tän JCI:n myötä tai siellä työryhmissä rakennettuja, mutta onhan näitä rakennettu täällä. [Palse? 00:44:15]-potilailla on oma selkee, mun mielestä skeema millä ne menee läpi. Ja just nää puudutuspotilaat ja...

Puhuja 2 [00:44:25]: Joo, mut tää on hyvä esimerkki siitä, et kun sulla on ongelma, sä pistit ihmisiä miettimään ratkaisua siihen, niin yleensä se löytyy. Konkreettisia asioita tekemällä laatu paranee.

Puhuja 1 [00:44:41]: Joo. Et kyllähän niitä on tehty, mutta se että ne on tehty työn... Työstä nousseena ongelmana, tai semmosena et saadaan niinkun homma toimimaan. Kyl ne varmaan sitten... Ehkä ne jotenkin sitten nivoutetaan tai sisällytetään sit siihen laatukäsikirjaan, näitä.

Haastattelija 2 [00:45:07]: Mitäs jos te aattelette tätä tulevaisuutta, niin näättekö että tulevaisuudessa laatujärjestelmä olis kuitenkin käytössä ja sillä olis jotain vaikutusta jatkoon, vai ajatteletteko että tää on tämmönen harjoitus minkä voi unohtaa sitten pitkällä tähtäimellä?

Puhuja 2 [00:45:25]: Tää harjoitus pitäis unohtaa, mutta laatu on tulevaisuutta. Se on ilman muuta niin, et ilman sitä laatua ja ilman sen laadun mittaamista ja sen esittelyä ei oo mitään

jakoa tulevaisuudessa. Mutta tää nimenomainen juttu, tää on lähtenyt minusta väärästä päästä ilman mitään selvää suunnitelmaa ja sellaisenaan niinkun se sillon ajateltiin, niin ehkä se oli kaunis ajatus, mutta kun ei se toimi, niin musta ainakin se täytyis sit kattoo että mikä on se tavoite mitä me tässä tavoitellaan, et se ei saa nyt olla se että meillä on joku tietty systeemi jossa sitten saadaan joku sertifikaatti. Se on, niinkun... En osaa oikein nähdä hyötyä siitä. Se voi olla tämmönen hallinnollinen toive, että me voidaan sit mainostaa jollain tämmösellä tavalla, mutta... Et ei taida nyt kovin nopeesti toteutua.

Puhuja 3 [00:46:33]: Just tuli mieleen sellanen, kun ne työryhmät... Varmaankin yks ongelma on sekin, että millä tavalla ne on muodostettu sitten ja ketähän niissä sitten loppupelissä on, kun se taitaa olla vähän semmosta että menetkö sä sinne vaikka ihmisillä ei oo minkäänlaista valmiutta, vahvuutta, ei mitään tietoa, ei mistään asiasta mikä siihen liittyis. Tätä se ongelma on kanssa, että on paljon semmosia asioita, elikkä meille vain sanotaan, että teetkö sitä tai meetkö siihen kokoukseen, otatko kantaa, laitatko viestiä. Ja on aivan niinkun et no, mistä ihmeestä mä tiedän. Eli siis se just, että nää vahvuudet mitkä ihmisillä on, niin ne otettais käyttöön enemmän ja hyödynnettäis niitä ja siis ylipäätänsäkin, kaikki koko se työntekö, niinkun siis sillä, et sehän olis... Sehän... Kaikki sujuis paljon joustavammin ja sehän antais semmosen hyvän kuvan myöskin sinne asiakkaille, meidän potilaille ja sitten kaikki vastaavasti taas henkilökunnasta tietäis että mulla on ongelmia vaikka fysioterapian kans, niin mä tuun sun luo, mulla on selkäjuttu mä tuun sun luo, mulla on haavahoidot, sun luo. Laskutusta, tule mun luo... Tiedäts sä silleen, että olis, niinkun... Kaikki nyt on niinkun vähän silleen hajallaan ja sitten siinä... Se on kuin muurahaispesä välillä, siis oikeesti. Sen yhden ympäri kaikki juoksee jonkun papereiden kanssa. Siis siitä tulee semmonen helvetin kaaos ja se näkyy myöskin mun mielestä potilailla aina välillä, et se... Se kaaos, et sen, niinkun... Et vaikka kuinka kiire olis tai jotain olis, mut että jos meillä olis semmoset tietyt asiat ja tietyt säännöt ja tietyt asiantuntijat, niin se loppuis, niinkun semmonen. Ja sit onhan tässä haasteena. Ja sit onhan tässä haasteena tässä talossa myös se, että on hyvin... Kun tää on joskus ollut näitä osastoja miljoona, niin tällä hetkellähän tää talo, niinkun me ollaan fyysisesti yks tuolla, toinen täällä ja muuta, niin se semmonen logistinen, et kaikki on vähän niinkun hyvin hajallaan ja sitten oote, mä haen sen. Meni viis minuuttia, kun mä käyn kipittää sen jostain tuolta. Et kyllähän se semmonen että asiakkaat sanoo että onpas tämä sokkeloinen tämä meidän talo. No joo, mut... Niin. Mut kun se on muuttunut isosta ja sitten niitä on... Sillehän ei voida mitään. Mutta se on varmasti niinkun, et yhtäkkiä (…) ilmoittautuminen onkin meidän ilmoittautumisemme. Ja semmonen, et sekin aiheuttaa varmaan vähän semmosta...

Puhuja 1 [00:49:28]: Joo, tää on tosi sekava ja siitä oikeasti tulikin mieleen... Siitä ei oo kauheen kauan aikaa sitten, kun hirveä huuto kuului ja askeleet tuli lähelle. Joku vanha ihminen huusi, että onko täällä ketään ja se tuli lähemmäs kato koko ajan siihen sun huonetta ja lähemmäs. Mä ajattelin, että mä en nyt tajua, että mistä täällä on kyse, kun talo on täynnä ihmisiä. Hän tuli sieltä pääaulasta ja kun mä hamppasin sieltä ovesta ulos, sit mä huomasin sen et se oli opaskoiran kans sokea mummo. Kun mä sitten, niinkun... Mä, että on

täällä, mihin tää rouva nyt on menossa ja se oli et no, onhan täällä joku ja minä oon tulossa nyt sairaalaan. Mä tietenkin automaattisesti, niinkun siis silleen, että mä oon että niin, että te meette silmäpuolelle, (…) silmäklinikalle. En mene. Mä, että joo, no mihinkä. No, (…) olen tulossa. Mä, että no, sinne silmäklinikalle te ootte menossa. No, kun ei ole, se mummo. Ja mä olin väkisin sitä... Silmäklinikalle, totta kai. Niin. Sit se oli... Mä, että onko teillä polvissa vika vai selässä vika vai lonkassa vika vai missä se vika on. No, minulla on selkä kipee. Ihan, niinkun, se oli [huominen? 00:50:45] sit ihan, mut siinä just on semmosta. [?? 00:50:45] oli sen pitkän käytävän tullut ja huutaa koko ajan et onko täällä ketään, et voisko joku auttaa.

Haastattelija 2 [00:50:54]: Voi ei.

Puhuja 1 [00:50:57]: Niin kukaan ei auttanut häntä. Ja sit pääaulasta, kun hän oli sanonut että hän menee (…), hänelle oli vaan sanottu että menkää suoraan, niin se mummo sano et jumalauta, eikö ne ihmiset tajua että mä oon sokee. Menkää suoraan. Siellähän tuli seinä vastaan. Et ihan niinkun tämmösiä just, että...

Puhuja 3 [00:51:22]: Mä oon joskus miettinyt, et tänne vois tulla jonkun sisustusarkkitehtisuunnittelija kuka vois miettiä et voidaanko vaikka väreillä tiedäks sä vaikuttaa siihen et näkee et nyt vaihtu (…) tai jotain. Semmoseen pitäis, niinkun... Se on se, et... Mä oon sanonut tän aikasemminkin ja mä sanon tän uudestaan - me ei voida loputtomasti menneen ajan maineella ratsastaa. Ja se on mun mielestä semmonen, että se on myös semmonen et niinkun sä sanoit, et laatua pitää seurata. Laatu on se minkä takia ihmiset tänne tulee, mistä mistä ne tunnetaan ja meidät tunnetaan ja jos siinä rupee tapahtumaan jotain heikkenemistä niin moido. Mutta se, että se pitäisi myös näkyä se laatu myös vähän ulkoisesti.

Haastattelija 2 [00:52:11]: Sanoit aikasemmin että jonkun verran on ollut ihmisten vaihtuvuutta, tullut paljon uusia ihmisiä, niin onks täällä ollut jotenkin enemmän nyt kuin aikasemmin sitä vaihtuvuutta.

Puhuja 1 [00:52:20]: On.

Puhuja 2 [00:52:22]: On ollut nyt.

Haastattelija 2 [00:52:23]: Onks sitä selvitetty että mistä se johtuu että ihmiset vaihtaa työpaikkaa, koska sehän on nyt ihan selkeesti työhyvinvointiasiakin, tai tämmönen.

Puhuja 2 [00:52:33]: Meille on varmaan tullut ihan konkreettisesti lisää.

Puhuja 3 [00:52:35]: Lisää, joo.

Haastattelija 2 [00:52:37]: Niin, ettei oo lähdetty...

Puhuja 2 [00:52:39]: Ei oo silleen pysyvyysongelma.

Puhuja 1 [00:52:43]: Ei.

Puhuja 2 [00:52:44]: Tässä on toimintaa ollut enemmän, yksinkertaisesti.

Puhuja 1 [00:52:49]: Mutta on meillä polilla kyllä ollut, et on tullut ja mennyt...

Puhuja 3 [00:52:52]: [päällekkäin puhumista 00:52:53] mennyt, mutta [päällekkäin puhumista 00:52:54].

Puhuja 1 [00:52:55]: Muutaman kuukauden päästä niinkun pari ihmistä, vaikka kolme lähti ehkä saman tien... Tai ei nyt ois... Jos oliskin mennyt saman tien olis ollut parempi, mutta et me käytettiin hirveesti aikaa niihin ja oikeesti, niin... Mut siinä on vaan ongelma, kun on ollut vähän liian sekavat kuviot, niin ne ei vaan, niinkun...

Puhuja 3 [00:53:15]: Siellä on vaikea päästä sisään.

Puhuja 1 [00:53:19]: Vaikea päästä sisälle, jos sä tuut ihan täysin ulkopuolisesta maailmasta, semmosessa missä sä et oo ollut ikinä, tehnyt mitään vastaavanlaista työtä, niin siis tosi vaikee nappaa kiinni siitä, kun tää on ihan... Siis se tietopankki mikä on, niin se on tässä ja se levy alkaa olla itelläkin ihan täynnä välillä kun kaikki pitää olla niinkun päässä. Ihan eurosta lähtien pitää olla päässä kun ei tuu koneelta, niin on se ihan...

Puhuja 3 [00:53:47]: Joo.

Haastattelija 2 [00:53:48]: Tohon jos keksis jonkun ratkaisun, ettei oo ihan...

Puhuja 1 [00:53:52]: Niin, ettei tarviis niinkun silleen. Ja se kaikki mikä sulla on päässä sun pitäis [viedä? 00:53:57] sen toisen ihmisen päähän, että kun ei tuu sitä meidän tietokannasta mistään, eikä oo mustavalkoisella mitään.

Haastattelija 2 [00:54:09]: Meillä alkaa hiljalleen aika loppua. Onko teillä jotain mitä haluaisitte lisätä, kommentoida, tai?

Puhuja 1 [00:54:14]: [?? 00:54:14].

Haastattelija 2 [00:54:26]: Mun mielestä hyvä pointti oli, että se laatu on myös tämmösiä fyysiseen toimintaympäristöön liittyviä asioita, että se ei oo vaan jotain tiettyjä prosesseja, vaan se on myös kokemusta siitä laadusta.

Puhuja 3 [00:54:43]: Kyllä ja laatu siitä, että sinusta... Tietyllä tavalla mä nään sen myös, että se laatu tulee... Tai semmosena että työnantajan kautta välittyy se että meidän työtä arvostetaan, niin se näkyy myös semmosena että se työympäristö pidetään viihtyvänä. Me jokainen voidaan vaikuttaa työympäristömme viihtyvyyteen. Sehän on ihan sitä, että me myös ollaan iloisia ja me ollaan... Sekin vaikuttaa, mutta myös ne semmoset fasiliteetit, että ne on OK, niin sekin on työn laatua ja lisää työviihtyvyyttä.

Puhuja 2 [00:55:19]: Hoksaamista. Nyt tuli äitienpäivä ja tuolla osastolle... Siellä oli jokuisia potilaitakin sillon sunnuntaina sisällä ja hoitajat siellä pahoittelivat et oli se vähän keljua kun ei tullut äitienpäiväkakkua, että olis voinut tarjota. Siellä oli äitejä ja isoäitejä. Se on niinkun semmonen hoksaamisesta ja nohevuudesta kiinni, et tulee mieleen ja ymmärtää tämmösiä asioita. Mutta jos ei tuu mieleen, niin sit ei tuu mieleen ja musta tuntuu että se laadun hierominen väkisin päähän, ei se sillä tavoin tapahdu, onnistu. Ihmisellä pitäis olla vähän semmosta fiinimpää järkeä.

Puhuja 3 [00:56:07]: Joo, niin.

Haastattelija 2 [00:56:06]: Niin, et joku tajuais että okei, tulee äitienpäivä, että tilaanpa tähän nyt yhden kakun, että se nyt ei tää talo mee siitä konkkaan.

Puhuja 3 [00:56:12]: Sehän ei oo juuri mitään.

Puhuja 1 [00:56:16]: Niin.

Puhuja 3 [00:56:17]: Se ei oo rahassa mitään, mutta se osoittaa sitä...

Puhuja 1 [00:56:19]: Arvostusta.

Puhuja 3 [00:56:19]: Se on juurikin näin.

Puhuja 1 [00:56:21]: Te olette huomioineet tämmöisen asian.

Puhuja 3 [00:56:26]: Ja se, että just vaikka naistenpäivänä, niin oikeasti, vaikka se nyt ei oo mitään, mutta naisiahan me ollaan. [?? 00:56:30] tuut aamulla töihin [?? 00:56:32] jonkun ruusun, tiiäts sä. Orava ite vaikka kävis antaa tiiäts sä silleen, et olepa kuule hyvä ja kiitos. Sekin ois...

Puhuja 2 [00:56:39]: Onneksi meillä on Aleksi tässä talossa.

Puhuja 1 [00:56:43]: Niin, Aleksi ei kyllä viime naistenpäivänä muistanut meitä. Hän ei enää työskentele kato polin puolella, niin hän ei sitten huomioinut meitä. Et sit me aina huolehditaan toinen toisesta, tai siis silleen että porukalla aina keksitään jotain juttuja, että tuo munkkia ja sitä ja tätä ja tota, että siinä sit ollaan. Mut työnantajan puolella vois tulla semmonen, että ihan pieni juttu. Tosi pieni. Ei sen tarvii olla mikään isokaan. Eihän me mitään timanttisormuksia haluta eikä mitään semmosta.

Puhuja 3 [00:57:13]: Saadaan (…) puisia pöllökelloja.

Puhuja 1 [00:57:17]: Niin.

Puhuja 3 [00:57:16]: [?? 00:57:17].

Puhuja 1 [00:57:19]: Onks niitä vielä?

Puhuja 3 [00:57:21]: Älä multa kysy. Sä sanoit et sä halusit semmosen.

Puhuja 1 [00:57:24]: [?? 00:57:24].

Puhuja 2 [00:57:25]: Ehdotetaan. Kesäjuhlissa.

Puhuja 3 [00:57:31]: (…) pöllökello. Joo, mut hei ja mun mielestä kaiken kaikkiaan... No, tietyllä tavalla kun mä en oo ite nyt ollut pitkään aikaan missään kokouksessa, kun oon ammatinharjoittaja, niin ehkä tästä myös herää semmonen mulle ajatus että kuinka paljon näistä asioista myös tietyllä tavalla puhutaan, et onko meillä semmosia, niinkun mä muistan et joskus meillä oli niitä yhteisiä palavereja, jotain viikko... Auditoriossa, muistatte, jossa asioista puhuttiin ja siellä sai kaikki tuoda niitä asioita julki. Et kuinka paljon tänä päivänä, kun asia on muuttunut sellaseks että työ mitataan siinä että kuinka monta suoritusta sä teet päivässä. Työkulttuuri on muuttunut. Joskus istuttiin kahvilla ja rupateltiin ja tänään eihän siihen oo ihan hirveesti aikaa. Mutta se, että kuinka paljon meillä on sitä, että meillä on aikaa keskustella ja kehittää niitä omia prosesseja tai ihan pieniäkin juttuja. Niinkun (…) sano että ei tartte kuuta taivaalta, vaan se voi olla joku yks, pieni asia. Kuinka paljon siinä on henkilökunnalle annettu aikaa? Kuinka paljon niistä käydään keskustelua? Ja tääkin missä me tehtiin tää, niin tässä nyt heräs monta semmosta ajatusta et onko semmosta. En minä tiedä.

Puhuja 2 [00:58:52]: Ne ovat hävinneet ne tilaisuudet, pikkuhiljaa. Kaikki on kuihtunut pois.

Puhuja 3 [00:58:57]: Niin.

Puhuja 2 [00:58:59]: Jotenkin arkipäivän työ on uinut joka paikkaan.

Puhuja 3 [00:59:03]: On.

Puhuja 1 [00:59:03]: Mutta esimiestasolla sitten jos jotakin ehdottaa, niinkun meillä esimerkiks, tai jostakin asiasta puhuu tai puhuu vaikka niistä sähköpöydistä tai ihan mistä

vaan, niin aina on vastaus se että no, tämä nyt on tämmöistä kuin tämä on. Piste. Siitä ei vaan niinkun puhuta. Piste. Terve. Joo, meepä takaisin sinne kuule sinne [päällekkäin puhumista 00:59:21].

Puhuja 3 [00:59:22]: Tai sitten sen kirstun päällä istuu joku semmonen ihminen kuka määrittelee, saadaanko me mihinkä rahaa, joka ei ehkä nää sitä... En tiedä näkeekö se sitten aina sitä kokonaisjuttua, että mihin sitä asiaa tarvittaisiin, jotta se homma sujuis. En tiiä. Mutta hei...

Haastattelija 2 [00:59:39]: Mutta hei, kiitos teille. Me ollaan yliaikaa. Kiitos tosi paljon. tää on tosi mielenkiintosta.

[recording ends]

Name of recording: Haastattelu 3_2022.mp3

Length of recording: 00:48:31

Information:

[?] = Word was not understood completely but meaning is almost correct. Recording point is written to the text e.g. [word? 00:15:44]

[??] = Word could not be understood and therefore could not be written. Recording point is written to the text e.g. [?? 00:15:44]

[text] = Sounds or not transcribed parts are written with square brackets e.g. [laughs] or [interview paused due to a phone call]

[recording starts]

Puhuja 1 [00:00:01]: Meit on muutama henkilö tässä.

Puhuja 2 [00:00:04]: Et sä oo yhtään sen turhempi kun minäkään.

Puhuja 1[00:00:05]: Okei. Joo. Joo.

Puhuja 3 [00:00:09]: Se onkin varmaan yks pointti täs tutkimuksessa, et se JCI varmaan hylätään, koska (…) on sen nyt tällä hetkellä hylännyt, ja sitten nehän ottaa jonkun tämmösen ISO, onks se ISO 5000 tai joku tämmönen siihen tilalle. Ja tää JCI on osoittautunutkin aika raskaaks.

Puhuja 2 [00:00:27]: Kyllä.

Puhuja 3 [00:00:27]: Siinä niin pikkutarkasti käydään kaikkia aspekteja läpi, mutta sikälihän on hyvä, että on joku tämmönen laatujärjestelmä, koska nehän on vähän niin kun manuaaleja. Vähän niin kun lentokoneen manuaali, ku tulee uus perämies sinne, se voi siit manuaalist kattoo, että missä järjestyksessä lähetään lentoon ja...

Puhuja 2 [00:00:48]: Toki se on nyt [?? 00:00:48], koska se on niin pikkutarkkaa, niin eihän oo mitään mahdollisuuksia ollu tehä sen eteen osastollakaan mitään, ku ei oo, tommoseen työhön, siihen pitäs irrottaa ihminen.

Puhuja 3 [00:01:01]: Plus sit...

Puhuja 2 [00:01:02]: Ei pysty oman työn ohella niinku tekemään.

Puhuja 3 [00:01:04]: Joo. (…) ne pääs paljon pitemmälle, mutta ne on koko aika huomannut, et se on varsin raskas.

Puhuja 2 [00:01:08]: On ja siel on varmaan ihan nimetyt, omasta työstään irrotetut ihmiset tekemässä sitä. Käsittääkseni. Oli ainakin sillon alkuun, niin.

Puhuja 3 [00:01:18]: Mut eihän tää hukkaan mee tää työ, koska siinä ISO-standardissa on ihan samat jutut, se on vaan vähän pelkistetty versio siitä. Niin keskitytään ehkä enemmän

oleellisuuksiin. Se on vissiin vähän se päätös viel kesken, että hylätääks se JCI kokonaan, vai et yritetäänks sit vielä siinä.

Haastattelija 1 [00:01:43]: Ni täällä vai (…)?

Puhuja 3 [00:01:45]: Sekä että. Mä en tie, onks (…) tehty sitä päätöstä, että se, mut toi (…) sanoi, että luultavasti (…) hylkää sen. Siel on vähän viidakko puhunut siitä. Mutta tota siis, siirtyy sit siihen ISO 5000 jotain -järjestelmään.

Haastattelija 1 [00:02:06]: No ootteks te mitenkään osallistunut tähän JCIhin tai minkään laatujärjestelmän rakentamiseen?

Puhuja 1 [00:02:11]: Kyl me tähän JCIhin, niin kun mul on ollu se potilaan arviointi, niin mä oon ollu siinä ryhmäs mukan, mut se on hyvin, me ollaan hyvin satunnaisesti nähty ja ihan muutama kerta, pari kertaa, että. Joo, ja mä oon sit nimenomaan sitte ton meiän kipupoliklinikan toiminnan kannalta niitä niin kun sit, sitä puolta tuonut siihen. Sit on ollu erikseen just Elina Peltoniemi, joo, niin on tota osastoo ja sit on ollu Kristiina Sarkkinen tota kuntoutuspuolta siin miettimässä.

Puhuja 4 [00:02:56]: Innostuin tekemään sitä potilasturvallisuusosuutta, mutta mäkin olen jäänyt pois siitä sen takia, että tätä tehään työn ohella, ni mun työtilanne ei nyt mahdollistanut sitä, et mä olisin tehnyt mitään ylimäärästä, niin kun Jaana sano, niin täs pitäis olla ihminen, joka keskittyy kahdeksan tuntia päiväs tähän, koska sit se on niin laaja kokonaisuus, et sit kun sä rupeet jotain tekemään, ni siihen ei riitä puol tuntii tai tunti sillon tällön, että siin pitäs olla niin kun päiviä varata tälle. Ja sitten, jos tietty semmonen pikkutarkka tota kokonaisuus, että se että mitkä kuuluu meiän työnkuvaan ja siel on kaiken maailman elinluovutukset ja elinsiirtotodistukset ja sun muut, et mikä on niin kun meille realistista. Niin mitä me tarvitaan. Aika raskaan olonen koneisto on tämänkin kans kii, siin on kaiken maailman niinku sertifikaatit ja mitä auditoidaan ja pitää olla niinku, koko talo henkilökunta on jotenkin tietonen siitä, että jos tulee auditoija ja kysyy, niin jokaisen talon henkilökunnan, jokaisen pitäis osata vastata, et mistä se vastaus löytyy. Ei tällä liikkuvuudella. Ehkä vähän kömpelö.

Puhuja 2 [00:04:11]: Joo, mä oon jopa niis alkukokouksis ollu, mut sit meil on kaks niinku sairaanhoitajaa nimetty. Nimetty, (…) on just, mut se on ollu tän, alottanut tän kuntoutuspuolen ja sitten eli (…), mutta hänkään ei oo ehtinyt enää pitkään aikaan tekeen sen eteen yhtään mitään. Pahotellut, mutta.

Puhuja 3 [00:04:35]: Mä olin siinä alussa, ku oli se JCI-koulutus, sillon kun se esiteltiin talolle, ja otettiin niin kun projektin alle. Ja sit mä oon ollu niissä etäkokouksissa. Nyt koronan aikaan ne on ollut kaikki etänä. Aika vähän on ollut osallistujia sit niihin JCI tämmösiin katsauksiin, mitä on käyty aina kahen kuukauden välein. Ja sit mun myöskin, mä olin data tai tämmösessä tietoryhmässä. Mä en muista, mikä osuus se on. Mutta meil ei oo viel yhtään kokousta ollut siitä ja mä en tiedä, ehkä Klea ja Satu on siin keskenään vähän katellut juttuja, mutta siin yhes vaiheessa ainakin oli tietojärjestelmä niin suuressa remontissa, et ne sano, et ne ei nyt ehtis sitä jCI:tä miettiin yhtään.

Haastattelija 1 [00:05:22]: No miten te koitte ne JCIhin liittyvät koulutukset ja nää? Kahen kuukauden välein. Oliks mitään hyötyä tai?

Puhuja 3 [00:05:28]: Koulutus oli mun mielest tosi hyvä. Siin sai käsityksen siitä. Sillonkin tuli jo mieleen, että onpas tää aika pikkutarkka ja tosiaan käydään kaiken maailman varatilanteita läpi, jotka jossain toisessa sairaalassa varmaan onkin aiheellisia. Ja sitten, vaikeuksia vois, ja joka tapauksessa täs ISO-järjestelmässäkin tuottaa se, että kun me ollaan vuokralainen toisen toimijan alla ja sitte se on vielä muuttunut. Se oli ennen [?? 00:05:57] ja nyt se on toi eQ Kiinteistöt, vai mikä se on. Niin esimerkis tämmöset turvallisuusjärjestelmät, ni talo vastaa vaan osasta ja sit se taustajärjestelmä vastaa osasta ja se on hankalaa, et jos se taustajärjestelmä ei oo sit siin JCIssä, mut me ollaan. Eli kuka vastaa sit mistä. Se osoittautu hankalaks siinä ihan alussa. Sit ku tuli tuo korona tohon, just sit JCI oli ehkä puol vuotta ehtiny olemaan voimassa tai siis se projekti. Niin sitte siirryttiin etäkokouksiin ja sit se vähän lopahti. Kaikki tämmönen, meiltä se into. Mutta kyllä jotkut ryhmät mun mielest aika hyvin päässy niitä ohjeistuksia kirjottamaan auki ja vanhoja ohjeistuksia päivittämään uuteen aikaan.

Puhuja 2 [00:06:56]: Joo semmoseen se on ehkä saanu vauhtia, että on semmosta taustatyötä ruvettu tekemään ja näin.

Puhuja 3 [00:07:04]: Ja kyl mä sillain mä näen sen aika tärkeeks, et just, jos tulee vaikka uus työntekijä, ni sil ois ihan selvä manuaali, että mitä tämmösissä tietyissä tilanteissa tehdään. Siihen voi sit vedota, jos se tekee työvirheen taikka...

Puhuja 4 [00:07:23]: Nii ja semmonen perehdytyskansio-tyyppinen, mis on koko talon ja sit on tietyst osastoittain on omat, mutta että jokuhan, jo tää on iso talo ja tääl monta toimialaa ja paljon ihmisiä, jotka tekee ihan jotain muuta kun mitä itte tottunut täällä

tekemään, ni se, että se sais jonkun kuvan siitä, et mitä esimerkiksi (…) pitää sisällään ja miten täällä toimitaan, mutta tämä kyseinen JCI on kyl vähän turhan ehkä...

Puhuja 3 [00:07:47]: Sehän on tehty amerikkalaisia sairaaloita ja vakuutusyhtiöitä varten, että vakuutusyhtiöt sit herkästi haastaa taikka lakkaa korvaamasta sairaaloille jotain, jos ei se oo toiminut sen JCI-ohjeistuksen mukaan.

Puhuja 4 [00:08:01]: Ja paljonhan siinä on, kun sitä teki sitä työtä, niin oli se, et mikä on Suomen laki. Laki määrää, et totta kai Suomen lain mukaan mennään, vaikka se JCI määrää mitä tahansa, ni sitte en tiedä, et onks se niin kun meidän leipätyötä ottaa selvää Suomen laista ja verrata sitä, että mikä on niin kun totta. JCI määrää jotain ja sitten meiän pitää tällä koulutuksella ottaa selvää, mitä Suomen laki sanoo asiasta ja sitten niin kun kirjata se sinne ja se on ehkä niin kun, me voidaan kirjata se, et mitä me täällä tehdään ja, mutta se, mä laittasin työryhmän ja siihen, et kuka tätä työtä tekee, että se on, pitäs olla ihan joku siihen palkattu ihminen, joka ymmärtää myös mitä on tekemäs.

Haastattelija 1 [00:08:45]: Ootteks te muut käyny niitä koulutuksia? Kävitteks te alus, ei.

Puhuja 1 [00:08:49]: Mä en oo käynyt.

Haastattelija 1 [00:08:52]: No mitä te muuten ajattelette tämmösest, täs tuli jo puhetta siitä, et se on liian raskas toi JCI, mut jos mietitään yleisemmin näitä laatujärjestelmiä, niin musta tuntuu, että se on vähän kun perehdytyksenä uuteen työntekijälle ja että se on vähän kun manuaali lennonlähtöön.

Puhuja 1 [00:09:08]: Ja onhan se vähän semmonen jonkunnäkönen standardi, sit varmistus, et se työ on jossain määrin laadukastakin sit, et menee jonkun protokollan mukaan.

Puhuja 4 [00:09:19]: Aika moni firman, esittelee sen oman niinku laatutyökirjansa tai minkä mukaan on standardit asetettu, että kyllä joka paikassa, kilpailutukses kysytään aina, että mitkä on ne meiän laatutyövälineet tai mikskä niit kutsutaan. Mut se, et...

Haastattelija 1 [00:09:37]: Mitä te ootte tähän mennes vastannu, jos teil ei oo ollu?

Puhuja 4 [00:09:40]: Mä en tie.

Puhuja 3 [00:09:43]: Nyt on pari vuotta voitu sanoo, et me ollaan menossa tähän JCIhin.

Puhuja 2 [00:09:48]: Sillä on ratsastettu.

Puhuja 1 [00:09:51]: Vielä voi pari vuotta sanoo, et me ollaan nyt luopumassa.

Haastattelija 1 [00:09:55]: Siirtymässä ISOon jos niin käy.

Puhuja 4 [00:09:58]: Mut on ain, niinku kaikilla, esimerkiks Helsingin kaupungilla, ni tietyt laatustandardit, että on palveluseteli ja sit jos asiakas käy laittamas sinne merkit, että hyvin meni, niin se kasvattaa jotain pistepottia, sitten kun on tarpeeks, niin se tuleekin sit sinne palvelusetelijärjestelmään niin kun laatumerkintänä. Et totta kai kaikkien, tän alan työtä seurataan ja sil on, kyllä meillä joku pitää olla. Mut se, että mikä, niin se on eri juttu.

Haastattelija 1 [00:10:26]: Aatteleks te sen laadun, sen laatustandardin kannalt se suurimmat hyödyt tulee. Vai mitä te aattelette? Miten se niinku, mitkä on sen mahdollisuudet auttaa teiän arkipäivän työtä?

Puhuja 4 [00:10:40]: Se tekee hyvää välillä laittaa paperille se mitä täällä miettii.

Haastattelija 1 [00:10:44]: Niin kuvata sitä omaa toimintaansa. Prosessia.

Puhuja 1 [00:10:49]: Ja sit just se manuaali, et vois tarkistaa jotain. Et kyllähän niinku tämmösii on aina, sovitaan jostain yksittäisestä asiasta. Mut ei oo sit semmost ehkä kokonais, just semmonen perehdytyskansio-tyyppinen. Siinä ehkä hyödyttäis.

Puhuja 4 [00:11:08]: Ja on yksikkökohtasia perehdytyskansioita, semmosii niinku pienempimuotosia, mutta sitte ehkä semmonen yhteiskoonti. Tai olis ainakin selkeesti kaikilla tiedossa, et mistä se tieto löytyy. EIhän kaikkien tarvii sitä kansiota omistaa, mut se, intras on kohta mistä löydät vastaukset kysymyksiin.

Puhuja 3 [00:11:27]: Ja sit on, onhan nois standardit ja tommoset audioinnit sikäli hyviä, et jossakin on monta kertaa mietitty näitä samoja asioita, ja se on iteroiden se ohjeistus menny läpi jo monet muut sairaalat. Et turha keksii pyörää uudestaan, että jos me otetaan tämmönen valmis ja sit yritetään mahollisimman paljon sitä noudattaa, ni se voi olla helpompaa, kun keksii kaikki ite. Tietysti tää on vanha sairaala, tääl on omat toimintaohjeet ollu pitkään ja niitäkin on iteroitu. Mut ehkä on jäänyt jotakin niin kun miettimättä, mitä muualla on mietitty. Et tommonen standardi aina ni, se on aika, siin on paljon työtä takana. Ja siin on mietitty kaikkii asioit. Et siinä mieles ymmärtää sen arvon ja miks sitä yritettiin sitä JCItäkin. Se JCI tuli tietyst sitä kautta, ku (…) oli siihen menossa, ja sit (…) osti (…), niin tavallaan se tuli automaattisesti. Ja nyt kun, jos (…) on luopumassa siitä ja siirtyy tähän ISO-standardiin, ni sekin tulee automaattisesti sitten meille. Koska meil on niin paljon (…) kans yhteistyötä ja...

Haastattelija 1 [00:12:44]: No koetteks te, että se työ, mitä siihen JCIhin menee hukkaan, jos siirrytte siihen ISOon vai, tai sanoit jo aikaisemmin, et se kuitenkin pohjaa sit sillekin.

Puhuja 3 [00:12:52]: Periaatteessa se on pelkistetysti ihan sama asia. Samat ohjeet ja, niit on varmaan ehkä tehtykin ristiin silloin aikoinaan. Katottu oisko ISO-järjestelmä niinku nuorempi ja siin on varmaan sit JCIst otettu parhaat palat.

Haastattelija 1 [00:13:09]: NI ainakin sairaalaympäristöihin varmaan niin, koska voisi...

Puhuja 3 [00:13:13]: Ei varmaankaan menny hukkaan ja ainakin se työ, tavallaan rutiini, mitä sitä JCItä on nyt yritetty saada kokoon, ni se tavallaan jatkuu sit siin ISO-järjestelmässä. Mut mul ei oo sit kauheen hyvää käsitystä, mitä muut ryhmät on tehnyt, mut nä tiedän, et on aika paljon jotkut ryhmät saanu aikaan, että, sitä ohjeistusta, et niitä ei ehkä tarvi tehdä enää paljon mitään, muuta kun, et kirjottaa vaan sen puhtaaksi.

Puhuja 4 [00:13:44]: Ja ohan siel ollu paljon selkeitä lokeroita, mitkä me osataan täyttää. Mut sit siel on kyl...

Puhuja 2 [00:13:48]: Sepä se.

Puhuja 4 [00:13:48]: ... aika paljon myös sitä, mitä on täyttämättä. Ja sehän ei mee läpi ennen kun jokanen raksi on ruudussa.

Haastattelija 1 [00:13:58]: No puhuitte, et se vaatis jonkun yhen ihmisen tekemään sitä hommaa, et se on niin työläs tehä ainakin tän JCIn mukaan, mut...

Puhuja 4 [00:14:05]: Riittääkö yksi?

Puhuja 2 [00:14:07]: Ni ei. Edes se yksi, mutta...Eihän sekään, kukaan tota yksin pysty tekemään.

Puhuja 3 [00:14:13]: Joka osa-alueelle ryhmässä pitäs olla yksi, jolle riittävästi [?? 00:14:17] tunteja siihen, et se pystyy tekeen sen.

Puhuja 4 [00:14:22]: Kyllä meille on sanottu, että se aika pitää ottaa ja saa ottaa, mutta mistä sä otat sen, ni se on sit toinen juttu, ja sitte se, että kun on niin laajasta kokonaisuudesta kyse, ni sit sun pitäis oikeesti pyhittää se koko päivä, koska se homma hajoo sitten kun, jos sä oot niin kun vaikka pyhittäny puoli päivää, niin se, sä pääset käyntiin ja sit se niinku, homma rupee sujumaan, ni sit se loppukin se aika siihen. Pitäis olla ihan kokonaisii päiviä tai mieluummin pari, niin se on taas sit ehkä työajallinen kysymys.

Puhuja 2 [00:14:54]: Niin must tuntuu, et on ollu just semmosia jotain tunnin JCI-kokouksia sit niis pienemmissä, niin eihän ne etene niin ne asiat mihinkään.

Puhuja 4 [00:15:01]: Se että, se on, just todetaan mitä pitäs.

Puhuja 2 [00:15:05]: Niin ja ettei oo tapahtunu mitään. Sit se on se tunti menny.

Puhuja 1 [00:15:10]: Kyllä.

Haastattelija 1 [00:15:12]: Niin ja sit kuitenkin monet kohat on varmaan semmosia, missä pitää kuitenkin neuvotella ja jutellakin kollegoiden kanssa, et onks tää nyt näin vai näin ja sit se, et ei pääse ite siinä tekemisessä eteenpäin, kun menee siihen neuvotteluun.

Puhuja 4 [00:15:25]: Ja sit aika paljon sitä sisäistä verkostoitumista, et jos on se yks ala, ja sä et tiedä niihin vastauksia, niin sit mun pitää ottaa vaikka Jaanaan yhteyttä, että miten tää nyt teillä on ja sit mun tarttee ottaa johonkin muuhun yhteyttä talon sisällä, et miten tää

teillä menee ja... Se niin kun, sen tiedon saanti ja kerääminen on aika työlästä. Tottahan sie meet sun muut kaikki siihen haastattelurinkiin mukaan, niin hyödyllistä työtä, mutta en tiedä onko se semmost, mitä voidaan oman työn ohella tehdä.

Puhuja 2 [00:15:55]: Ei voida.

Haastattelija 1 [00:15:58]: Jospa se ISO ois sitten jotenkin, jos (…) päättää myös ottaa sen ISOn käyttöön. Jospa se ois vähän kevyempi, et se ois jotenkin hallittavampi se.

Puhuja 3 [00:16:06]: No ainoo mitä mä siit tiedän, et se on kevyempi. Ja jollain tavalla pelkistyneempi, et siin on keskitytty enemmän niin kun eurooppalaisiin ja, eurooppalaisiin ja kansainvälisiin sairaaloihin, ei niinkään tää JCI, joka aika paljon perustuu siihen, ettei vakuutusyhtiöt pääse niin kun huijaamaan sairaaloita. Jos ne heti huomaa, ettsairaala on rikkonut jotain JCI-ohjetta, ni sit ne alkaa vetäytyyn vakuutuskorvauksesta, ja sehä, sit käydään pitkii lakitaisteluita. No se JCI on vähän enemmän keskittyny siihen puoleen. ISO-standardi on varmasti, muuta mä en siitä viel tiedä, koska päätöstä ei oo tehty. Toi (…) tulee lomalta vielä vähäks aikaa töihin, ni sit se varmaan tekeen sen päätöksen sit muitten kanssa.

Haastattelija 1 [00:17:07]: Sä puhuit siitä laatustandardista, niin miten te aattelette sit tämmönen laatujärjestelmä vaikuttaa eniten siihen laatuun?

Puhuja 1 [00:17:16]: Miten se vaikuttaa, no ehkä se et, se yhdenmukaisuus sinne työhön, ettei tuu tavallaan mitään semmosii poikkeemii.

Haastattelija 1 [00:17:27]: Mites muut? Miten teiän työ paranis teiän mielestä? Mitä te aattelette, onks sillä vaikutusta teiän siihen työn laatuun?

Puhuja 4 [00:17:36]: No kyllä se tuo läpinäkyvyyttä myös sen, että mitä tehään. Ja miten, et se on. Ja totta kai joka talol on omat tavat tehä, mutta se, että niin kun, tuleehan laatu ja standardeista kaikki ammattieettiset hommat sun muut myös niin kun kirjattua ylös. Niin se on myös sitä niin kun, et tehään sitä, mitä meiän pitäiskin.

Puhuja 2 [00:18:01]: Potilasturvallisuutta. Totta kai paranee sen myötä, kun on selkeet ja yhtenäiset ohjeet.

Haastattelija 1 [00:18:12]: Jos nyt mietitään JCIn käyttöönottoa, niin missä vaihees teil ees se nyt on niinku tälleen käyttöönotto, et on tehty ja nyt ryhmät on kokoontunut, mut aatteletteks se on niin kun puoles välissä vai alle puolen välin vai ihan alkutekijöissä vai?

Puhuja 3 [00:18:29]: Mä veikkaan, et niitten ohjeitten kirjottaminen on ehkä puolessa välissä. Mut vielä ei oo mietittykään sitä, et tulis joku auditointi tänne ja et kaikkien pitäis osata ne ohjeet. Ei oo viel yhtään mietitty, et jos JCIssä jatkettais, menis varmaan ainakin kaks vuotta vielä ja jos korona jatkuu, ni sit se hidastaa edelleen sitä. Koska tota, jotkut kokoukset menee etänä paremmin, mut ainakin JCIssä sen on huomannut, et se ei etene juurikaan. Koska ihmisiä ei velvoiteta osallistumaan siihen, edes näitä ryhmien vetäjiä. Et tietyst jos velvotettais, et nyt sun pitää kertoo seuraavas kokoukses mihin saakka te ootte päässy. Ja pitää olla jotain semmosii välietappeja, mihin pitää pyrkiä, niin sitten ehkä se edistyis.

Puhuja 4 [00:19:28]: Vahvasti päätelty, et perustyö menee tän edelle. Jos sul on sitä perusduunia, ni sit sä et tee tätä. Ja ehkä talo on ihan onnellinenkin siitä, et perusduunii on ollut niin paljon, et ei oo edennyt, mutta ehkä puhun [?? 00:19:44] mutta se, että rivityöntekijän sormien läpi tää ei ehkä tuu koskaan valmiiks.

Haastattelija 1 [00:19:56]: No mitä te. onks tämmösel laatujärjestelmäl jotain vaikutust teiän työmotivaatioon? Et menee aina kaikki muut menee tietysti perustyö menee edelle, mut muuten, ni ajatteleks te, et sil on niinku...

Puhuja 1 [00:20:07]: Laatujärjestelmällä vai sen teolla?

Haastattelija 1 [00:20:10]: Varmaan täs vaihees sillä teolla, mutta valmistuttua sillä järjestelmällä.

Puhuja 3 [00:20:18]: Kyl se nyt tavallaan tuo semmosta turvallisuutta, et jos on joku laatujärjestelmä ja sä tiedät, kun sä teet sen työn laatujärjestelmän mukaan, ni sit se on riittävästi. Eikä sun tarvi miettii sitä, et pitääks tehdä jotakin ylimäärästä vielä tai, ja ja...

Haastattelija 1 [00:20:33]: Niin et se niinku työturvallisuuteen sitä kautta myös, että... Potilasturvallisuutta.

Puhuja 3 [00:20:40]: Varmastikin, jos tulee uus työntekijä tosiaan taloon ja sit sil ois tämmönen manuaali, laatujärjestelmä, niin se pystyy siitä sitten paljon tarkistamaan sen perehdytyksen aikana ja sen jälkeen, että mitä häneltä odotetaan, tekevän ja. Et kokonaisuudessa mä aattelen, et se on hyvä systeemi. Ja jonkinlainen pitää joka tapauksessa olla. Jonkinlainen ohjeistus ja sovitut toimintatavat ja, hyvä, et jos me saadaan paperille, ni sillon se on semmost jatkuvaa, et ihmisii jää eläkkeelle ja omistajat vaihtuu ja näin, ni sit on jotain kirjallisestikin.

Puhuja 4 [00:21:28]: En mä tie onks siit suoraa vaikutusta työmotivaatioon, et kyl mä teen töitä ihan jonkun muun puolesta kun laaturekisterin puolesta, mutta voihan siin ol tietysti sekin, et jos laatustandardi on jotenkin kauheen näkyvä ja ollaan kaikki pisteet ja hymynaamat ansaittu, niin tuoko se sitten lisää asiakkaita, sitä mä en tiedä, mutta. En mä ainakaan näin rivityöntekijänä tunnista itessäni semmosta, et mä tekisin jonkun standardin eteen omaa työtäni, et jokainen yhtä tasavertaisesti kohdataan.

Puhuja 2 [00:21:59]: Joo, en mä nyt oikeet vastaan, motivaatiota eti sitä kautta.

Haastattelija 1 [00:22:05]: Mä mietin, näetteks te sillä mitään yhteyttä niinku työhyvinvointiin? Kun puhuit siitä, että jos on...

Puhuja 2 [00:22:10]: Joo, siihen varmaan ennemmin.

Haastattelija 1 [00:22:13]: Niin.

Puhuja 2 [00:22:15]: Sitä kautta ajatellen, toki, kun on selvät ohjeet ja, on tuotu jo moneen kertaan esiin tästä tää uuden työntekijän perehdyttäminen ja näin, jos kaikki ohjeet on olemassa, että näette, se oo sit sen varas, että oonkohan muistanut tolle kertoa tota ja penkoo tuota laatikon pohjast tuota ohjetta, että, toki siihen.

Puhuja 1 [00:22:40]: Riskinä varmaan tulee vaan mieleen, että sit jos täs luodaan liian raskas tämmönen ohjeistus ja koneistus, niin sit, jos sä joudut toistamaan sen useesti jokaisen potilaan kohdalla ja se ei, tuntuu turhalta, et tää ei tarvitsisi tätä, voi sitä kautta, tai siis laskea motivaatiota.

Puhuja 3 [00:23:02]: Tai sit jos niist tulee liian raskaita lukea. Vähän niinku nää tämmöset lakitekstit. Kun otat jonkun ohjelmiston käyttöön ja sun pitää suostuu siihen litaniaan ja se

on lakiteknisesti, ei kukaan niitä lue. Ni ei tämmöstä laatujärjestelmästä ja standardista saa tulla niin raskasta ja yksityiskohtaista, et sitä ei kukaan jaksa lukea. Edes se uus työntekijä, joka tulee.

Puhuja 2 [00:23:28]: Niin kuitenkin toi käsikirja heti, kun tuli se ensimmäinen. Motivaatio, et kyllä näin, tavaa sitä läpi, että aattelin, että mikä työ täs tulee olemaan, ni.

Puhuja 3 [00:23:38]: Niin se on huomattu, esimerkiks lentokoneitten ne manuaalit, ne on hyvin selkeet ja pelkistettyjä. Nää on hyvin yksityiskohtasii. Jos tää on rikki, niin teet nämä vaiheet tässä järjestyksessä. Eikä siel oo mitään ylimäärästä, siel ei niinku käytetä kapulakieltä ja muuta. Kun kerrotaan, mitä se asia on ja teet tämän. Niin se ongelma ratkeaa. Siel on ongelmia ja ratkaisuja. Ongelmia ja ratkaisuja.

Haastattelija 1 [00:24:05]: Vähän semmonen, et niin kun kysymyksiä ja vastauksia, jotenkin.

Puhuja 4 [00:24:11]: Nii ja sitten johan siis, vähän lukijakohtaisesti se, että me olemme tottuneet lukee aika semmosta niin kun faktatietoo, lyhennettyä, että se, että mein mielenkiinto, tai ainakin henkilökohtaisesti meikäläisen mielenkiinto häviää ensimmäisen 10 rivin jälkeen, jos siit ei oo mitään tolkkuu siinä.

Haastattelija 1 [00:24:28]: Niin, elikkä se pitäs olla selkokieltä, mikä selkeesti koskettaa niin kun sen kohderyhmän.

Puhuja 4 [00:24:32]: Enemmän keittokirjamainen tyyli, ja sit varmaan, siin vois olla ISO-standardissa olla vähän selkeämpi se, kysymys ja vastaus -tyyppinen. Et meidän ei kaikkia kommervenkkejä tarvi tietää.

Haastattelija 1 [00:24:54]: Mutta jos mietitään sitä työhyvinvointia vielä, ni miten te aattelette, et mihin siihen työhyvinvoinnis tämmönen laatujärjestelmä, mikä mahdollisuus sillä on niinku muuta kuin se kaikki, se työturvallisuuden kautta kaikki tietää ja on yhteiset ohjeistukset, mut onks sillä, miten te näette sen yhteyden? Onks sitä yhteyttä oikeesti olemassa? Tai mikä siihen työhyvinvointiin sitte vaikuttaa, jos aatellaan, että täältä jotenkin, kun kuuntelen teitä, ni se, että se jotenkin on se ohut kuitenkin se laatujärjestelmäyhteys siihen työhyvinvointiin.

Puhuja 4 [00:25:27]: Jos ajattelee niin päin, et se laatustandardi tasapäistää niin kun ammatillisesti, että tää on se, mitä me tässä ammatissa tehdään ja kaikki on, niin kun samanarvoisia siinä oman ammattinsa sisällä, et se on sit persoonakohtaista, et kuka kuormittuu mistäkin ja mitä kukakin kokee. Mutta se, että tässä ammatissa, tässä talossa toimitaan näin. Niin tuohan se sit just sitä turvaa ja rajoja siihen omaan työskentelyyn, että jotkut, ne on taas niin kun tapaus- ja persoonakohtaisia ne, että miten ihmiset kokee ja reagoi asioihin, mut niil on aina taustalla sit kuitenkin se tämän talon ohjeistus. Se voi tuoda työhyvinvointia siinä, että on joku mihin peilaa.

Haastattelija 1 [00:26:14]: Vähän semmonen minimitaso siitä niin kun laadusta, et nää ainakin on kaikilla kunnossa.

Puhuja 4 [00:26:17]: Niin.

Puhuja 3 [00:26:18]: Parhaimmillaan toi JCI-prosessi tietysti ois toiminut niin, että nää ryhmät ois keskustellu siitä omasta työyhteisölokerostaan, että miten hommat on tehty ja mitä vanhoja ohjeita meil on ja mitä tehdään uusia ja sitte kaikki on kiinnostuneita siitä ja osallistuu siihen keskusteluun, että mitä me tehdään tässä osastossa. Niin ei tietysti nyt käynyt, mutta kyl se varmaan jotain on, kun ne ryhmät on kokoontunu muutamii kertoi, niin ne on ehkä niit vanhoja ohjeita vähän miettinyt uudestaan ja ettinyt niit pöytälaatikoista niitä, että ai niin tääkin ohje on täällä. Sillai se voi parantaakin sitä työyhteisöä parhaimmillaan. Mutta korona aika paljon sotki tätä JCI-prosessia, et en tie miten se ilman koronaa ois siitä mennyt.

Puhuja 4 [00:27:13]: Toinen hyöty varmaan semmosest toimivast laatustandardist ois se, että kaikki tietää mistä sen löytää. Jonka, se on yhes paikassa ja sinne on koottu kaikki, et jos mul on joku kysymys, niin mä voin itse käydä katsomassa sen sieltä intrasta tai jossain, et se on niin kun se tämän talon käsikirja. Ja nythän siel on kaikkien esimiesten tai muitten yksiköitten, pomojen siellä pöytälaatikoissa ne sovitut jutut, että talon kannalta on kyl hyvä, että ne kootaan yhteen ja on tämmönen yhtenäinen käsikirja, että miten, mitä me tääl puuhaillaan. Mutta, työtä se vaatii.

Haastattelija 1 [00:27:51]: No miten te ajattelette, jos ajattelette leikkaussaleja tai leikkaussalitoimintaa, ni miten se vaikuttaa siihen laatujärjestelmään tai sen rakentaminen? Siis sen potilasturvallisuuden kautta ja...

Puhuja 3 [00:28:06]: No siel on ennestään ollu aika paljon ohjeita, yksittäisii ohjeita, niin ainakin siellä ne nyt kootaan kaikki yhteen. Ja meillekin on tullu sillai tipoittain, esimerkiks

tämmönen leikkauksen alussa tehään tämmönen check list, joka taas on otettu ihan lentoliikenteestä, et ennen kun lähdetään lentoon, niin on check list, vaikka nää on semmosii turhanpäiväsii kysymyksiä, onko tämä asia hoidettu ja onko tämä asia hoidettu, mutta se vain käy ne läpi järjestyksessä ja sit vasta se lentokone on lentoon lähdössä. Samoin leikkaussaliin otettiin mukaan. (…) sen toi, se oli jossain nähny ja sit ajatteli, et tää on hyvä. Sit myöhemmin se on tullu vissiin pakolliseks kaikissa leikkaussaleissa Suomessa, et siinä ihan käydään tämmösiä asioita läpi, että onko tarkistettu kumpi puoli leikataan potilaasta. Onko anestesiassa jotain erikoisjuttuja. Onko kaikki työvälineet paikalla. Onko kaikki henkilöt paikalla. Tunteeko kaikki henkilöt toisensa. Ni se on ihan hyvä check list, joka on käytössä nyt leikkaussaleissa. Ja tää on tietysti yks tämmönen [?? 00:29:23] joka tulee sit tähän standardiin. Sit siel on anestesian puolella omansa ja välinehuollossa.

Puhuja 2 [00:29:31]: Se alkaa jo polilta, että kaikilla osioilla on se oma check.

Haastattelija 1 [00:29:38]: Mut voisko teiän mielestä semmonen check list niinku korvata sitä laatujärjestelmää osittain tai, sanoit, että se voi olla osa sitä.

Puhuja 3 [00:29:46]: On se korvannut. Niin kauan kun se leikkaussalissa ollut käytössä, niin se on ollut hyvä ja se on tavallaan korvannut sitä varsinaisempaa, sitä muuttuvaa standardia. Ja jospa siihen JCIhin kuuluu tää check list. Leikkaussalin puolelle.

Puhuja 1 [00:30:08]: Mut eiks se nyt oo tuonut myös laatua meiän leikkauspuolelle, että ne on, menee niin kun se on suunniteltu ne leikkaukset, että.

Puhuja 3 [00:30:15]: JOo, koska kyl niit on tapahtunut. Ei meillä ehkä, mutta on leikattu joskus väärää puolta (…) ja...

Haastattelija 1 [00:30:20]: Niitä lukee aina välillä lehissä, että leikattu väärä polvi ja...

Puhuja 4 [00:30:25]: Mut kyllä se pitää oma henkilöllisyys todistaa aika monta kertaa ennen kun sä unessa olet, kun sä lähet huoneesta, ni kysytään, et kuka oot ja ku sä meet, ni kysytään kuka oot ja ennen kun oot unessa ni kysytään vielä kerran, et kuka oot. Et varmasti on oikee tyyppi. Niin tämmöset niinku, vaikka tuntuu semmoselt, et johan tää on kyselty, mutta ei varmaan näis asiois voi liikaa varmistella.

Haastattelija 1 [00:30:50]: No mitä te muuten aattelette, et potilaskohtaamista ja potilasturvallisuutta ja sen, tai jos mietitte teiän työtänne potilaitten kanssa. Ni nää check listit, ni miten muuten se laatujärjestelmä jotenkin, mikä laatujärjestelmän kohta on siinä, ku te potilaitten kanssa. Miten se muuttas teiän potilaitten kohtaamista tää laatujärjestelmä? Tai muuttaaks se mitään?

Puhuja 4 [00:31:24]: En mä ainakaan koe, et mä muuttaisin mitään.

Puhuja 1 [00:31:27]: Voihan se muuttaa jossain määrin, et kun meilläkin on tuol kipupoliklinikalla on tietyt esilomakekyselyt ja tietty prosessi, et kun ne tulee vastaanotolle, ni jos se jotenkin päivittäis sitä. Sitten. Jos se sitä kautta muuttuis, mut et kyl meil on jo vähän tolleen hiottu se toiminta. En mäkään näe, että siit tulis mitään kauheesti muutoksii.

Puhuja 4 [00:31:55]: Ehkä se liittyy, vois liittyy siihen tiedonkeruuseen ja siihen niin kun ennakko... No kuntoutuksen puolella, niin voi, mulle voi tulla asiakas, mä tiedän tasan vaan nimen. Mä ajattelen, että se on ainoastaan se vala, hänen kertoman tiedon mukaan liikenteessä. Ni tietysti, jos on mahollisuus saada sitä informaatiota etukäteen, ni se on niin kun ain positiivista, niin sit se, et jos on tommoset, no poliklinikalla onkin selkeemmät sabluunat, mut kuntoutus on vaan tommosta, [?? 00:32:25] ajoiltaan, niin se että, siel ei oo niin selkeetä sitä, et miten jokainen asiakas otetaan vastaan, et jokainen asiakas otetaan vastaan sillä tyylillä, millä sattuu. Että se voi joillekin tän talon osa-alueille tuoda tarkkuutta ja semmosta järjestelmällisyyttä, mutta se, että kohtaanko mä asiakkaan eri tavalla, niin kun siin vastaanottotilanteessa, niin en usko, että se vaikuttaa mun työhön siinä kohtaamisessa, että.

Haastattelija 1 [00:32:58]: No toivoisiks sä, et kuntoutuksen puolella ois enemmän jotain yhteisiä tapoja tehdä sitä työtä tai kysyä...

Puhuja 4 [00:33:05]: No onhan niit, tiedetään osaston jaksoilla, ni heillä on esitietokyselyt ja esisoitot sun muut, mutta sit kun meil on ajanvaraus, nettiajanvaraus, niin esimerkiks, siel voi olla vain nimi. Ja totta kai näitä yritetään näitä saada niitä työkuvia niin, että kaikki asiakkaat tulee esimerkiksi kassan kautta ensin ilmoittautumaan. Sit jos se onkin sun oven takana, niin sit sitä aikaa ei käytetä siihen, että nyt mennään sitten ilmoittautumaan, että onhan siel aina niitä semmosia aukkoja toiminnassa, mutta semmoset laatukäsikirjat ja ylipäätään perehdytyskansiot varmaan tuo selkeyttä siitä, et miten sitä työtä tehdään, mutta sitten ehkä tää talon maine on myös sekin, että löytyy aika paljon joustoo meillä sit. Mennään just kaikkien, tai et löytyy joustoo tähän touhuun, et tääl on, ratkasut löytyy sit vähän luovemmin, jos ei.

Haastattelija 1 [00:34:07]: Noin. No miten te ajattelette, et tämmönen laatujärjestelmä vaikuttaa tulevaisuuteen? Onks se tein mielest semmonen juttu, mikä pitää olla vaikka kahden vuoden tai viiden vuoden pääst kunnossa vai miten te aattelette sen? Puhuitte siitä rahoitus tai noista...

Puhuja 2 [00:34:22]: No kyllä kai se nykyaikaa on, että pitää olla.

Puhuja 1 [00:34:25]: Ainakin sen suhteen mitä puhuttiin, että kaikilla muillakin on. Yksityisil toimijoilla, ni sitte voihan se jossain määrin, no en oo kyl koskaan ite, kun vaik lapsille oon varannut aikaa (…), en oo kattonut, et mikä standardi tällä paikalla on. Et lähinnä asiantuntijoiden mukaan. Et en mä tiedä sitte. Vaikee sanoo, mut tuntuu kuitenki, et voihan sillä olla merkitystä.

Puhuja 4 [00:34:52]: No ei kai se vaikuttaa noihin kilpailutuksiin ja noihin kuntien kanssa. Joitten kans yhteistyötä, ja niil on jotkut määritelmät, niitten omat standardit, mitkä pitää sitten hakijan täyttää, veikkaisin, et laatustandardi pitää jonkinlainen olla, et ylipäätään on edes kilpailus mukana. Niin se on vaan yks vaan tämmönen niinku, mikä pitää tän kokosella yrityksellä olla.

Haastattelija 1 [00:35:17]: Ja tosiaan mietin ihan samaa, et enpä oo ikinä, vaikka mäkin oon kiinnostunu näistä laatujärjestelmistä, en oo kyllä ikinä lääkäriä varatessani miettiny mikä...

Puhuja 1 [00:35:25]: Ei ei. Ehkä kuulostaa hassult, jos sitä ruvettais markkinoimaan. Olemme ISO 5000 standardin mukainen toimija. Osta nytte.

Puhuja 4 [00:35:36]: Mut sen mä tiedän, et esimerkiks Helsingin kaupungin palvelusetelijärjestelmäs on niin, et jos sä saat niit hymynaamoja paljon ja sä saat hyviä arvosteluja, ni sä pomppaat sinne, tarjotaan. Niin kun kärkipaikoilla, et kyl tietysti semmoseen niinku potilasvirtaan voi vaikuttaa se, että jos palaute on positiivista ja tehdään työmme hyvin, niin sillon se näkyy myös tämmösissä ihan riviasiakkaallekin. Et ainakin joku on tykännyt siitä, miten täällä hommia tehdään. Kyl se varmasti vaikuttaa siihen tulevaisuuteen, siihen, et mitä asiakaskuntaa tääl jatkos pörrää.

Puhuja 3 [00:36:15]: Ja meiän omistaja varmaan tulee vaatimaan, koska ne, (…) itse jonkun laatujärjestelmän varmaan vie loppuun saakka jossain vaiheessa. Ja koko aika tekee sitä

työtä siellä, ni kylhän ne varmaan meiltäkin tulee vaatimaan, että me ollaan sit samassa järjestelmässä mukana. Ja juuri, kun ne sitten ohjaa potilaita tänne palveluseteleihin, niin siin vaiheessa ainakin niil on valmis se standardi, ni kyllähän ne sit varmaan vaatii, et sinne mihin ne ohjaavat potilaita, ni siellä pitää olla sit kanssa jonkinlainen laatujärjstelmä.

Haastattelija 1 [00:36:53]: Näetteks te muuten tulevaisuuden kannalta sillä merkitystä. Kun puhuit tosta markkinoinnista, että se ei oo kauheen todennäköst, ehkä.

Puhuja 1 [00:37:03]: Niin. Niin.

Puhuja 4 [00:37:07]: Mä en kuluttajana ajattele niin, että jos mä haen jotain palvelua jostain, niin mä vaan niin kun luotan siihen, et ne asiat on kunnossa. Ni se, että tietyt asiat on, mitä yritysten pitää hoitaa ja sit mä luotan siihen, et ne on hoidettu. En mä oikein tie, kuinka paljon se vaikuttaa sit niin kun, jos tämmöseen niin kun...

Puhuja 1 [00:37:28]: Ehkä sitä kautta, mitä säkin varmaan tarkotit, et sit jos se tois niin kun sitten laatua siihen työhön, ni se näkyy tykkäyksis sitten. Sil tavalla varmasti.

Haastattelija 1 [00:37:40]: Kaikilla hotelleillahan ne asiakkaiden pränkkijutut tuntuu pikkasen mun mielest vähän kuitenkin hassulta, jos (…), tai silleen, että työ on kuitenkin, niin voi olla, et se menee siihen.

Puhuja 4 [00:37:51]: Mut sit toisaalt, jos se tarkottaa sitä, et se, me täytetään se laatustandardin kaikki osaalueet ja se kans tarkottaa sitä, et meil on tietyn tasonen niin kun laatu tässä talossa. Ja se riittää jo siihen, että meil on se laatustandardi ja me täytetään sillon ne terveysalan standardit.

Puhuja 3 [00:38:11]: Kun miettii sit, et ois ulkomailla pitemmän aikaa ja siellä sairastus ja sit pitäis valita se sairaala. Ni kyllä mä menisin semmoseen, mikä on jonkun laatustandardin täyttänyt ja, joko JCI- tai ISO-systeemin, kun johonkin toiseen, joka ei oo. Ennen kun mä tiedän niist sairaaloist mitään.

Puhuja 1 [00:38:29]: Se on ihan totta. Tossa se toimis. Toises maas.

Haastattelija 1 [00:38:32]: Niin. Tos kun ei tuntis olleenkaan niitä.

Puhuja 4 [00:38:36]: Kuin paljon meil on niit ulkomaalaisii, esimerkiks leikkauspotilaita, et vaikuttaako se?

Puhuja 2 [00:38:40]: Kyl kai niit, no...

Puhuja 3 [00:38:44]: Ja ainakin venäläiset mielellään tänne tulis leikkauksiin.

Puhuja 2 [00:38:48]: Pohjautuuko ne laatustandardiin vai tietyn kirurgin mukaan?

Puhuja 3 [00:38:54]: Ehkä maineeseen, että. Nykyäänhän ne ei nyt tuu, ko ne ei pysty maksamaan sitä. Mut kyllä siellä keskustelupalstoissa ja suusta suuhun mennyt, ku meiltä on joku saanut tekonivelet ja muuta, niin ne kertoo, et siel on hyvä palvelu ja kotiutuu nopeesti ja ei oo kipuja ja kaikki toimii, ni.

Puhuja 1 [00:39:22]: Ja JCI tulossa.

Puhuja 4 [00:39:27]: Kuitin lopussakin vielä. Tekee se meiän laatua sit näkyvämmäksi sen. Alkuperäisestikin tehään hyvää työtä ja näin, mut sit vaan joku niin kun lisäarvo sille, et se tulee näkyväksi se, et miten täällä tehdään töitä. En tiedä.

Puhuja 3 [00:39:48]: Niin ja siin on just se, että hyvä, että välillä työntekijät miettii vanhoja sääntöjä. Pitäiskö niitä päivittää ja käydä läpi, et ne löytyy tosiaan. Tällasia. Se varmaan nostaa sitä laatua ihan itsestään.

Haastattelija 1 [00:40:05]: Niin se yhteinenkin.

Puhuja 4 [00:40:06]: Sitä on niin paljon hiljasta tietoo. Tääl on niin paljon ihmisiä, jotka on siis, voi sanoo, että vuosikymmeniä talossa. Nähny aika monta muutosta. Ja aika monta uutta tilannetta. Mutta sen hiljaisen tiedon saalistaminen olis ehkä kaikista hyödyllisintä tälle talolle, että. Nää, jotenki arvostettais näitä pitkään talossa olijoita ja niitten tietotaitoa ja, sen jos sais jotenkin kirjoihin ja kansiin, niin se ois.

Haastattelija 1 [00:40:40]: Niin, myös tää yhteinen keskustelu, että jotenkin tulis niin kun yhteinen käsitys, että kaikki tietäis ne asiat.

Puhuja 3 [00:40:47]: Parhaimmillaan se ois tosiaan toiminut sillä tavalla. Nyt, et noissa ryhmissä se, vaikka kaks ihmist puhunut keskenään ja ettinyt niitä ohjeita ja miettiny, että tätä hiljasta tietoa, et miten me saatais tää paperille. Ja voi olla, et ne on sit puhunut muittenki kanssa. Keskusteltu siitä, et mein pitäis nyt saada tää JCIn kaa tehtyy, että. Löytyyks teillä vinkkejä ja muuta. Kyl se varmaan, vaikka se nyt epäonnistui ja sitä ei viedä loppuun sitä JCItä, ei se hukkaan nyt kyllä mennyt sekään työ.

Haastattelija 1 [00:41:26]: Hei, mutta mitäs muuta te, tässä on oikeestaan mitä mä halusin kysyä, ni onks jotain, mitä te haluutte lisätä tai kommentoida tai jotenkin tähän laatujärjestelmään tai tän rakentamiseen tai työhyvinvointiinkin liittyen, että...

Puhuja 1 [00:41:45]: Ei tuu mitään mieleen.

Puhuja 4 [00:41:47]: Se on varmaan se, niin kun, jos ja kun seuraava laatustandardi tulee, sitä ruvetaan tekemään, niin se on selkeemmin se työryhmä mietitty sillä tavalla, että se on se työajallisesti, se on rivityöntekijän kauheen vaikee oman työn, työaikaansa jakaa, jos sanotaan, että perustyö menee kaiken edelle, mut silti tää pitäis saada tehtyä. Ota tähän niin paljon aikaa kun tarvitset, mutta mistä sä otat sen ajan, jos sitä ei ole. Niin, ja jos sit sanotaan, että kun suoraan kysytään, että saako tähän käyttää vaikka päivän, ni totta kai, jos työ antaa, työt antaa siihen mahdollisuuden. Mut ethän sä voi vaan sulkee sieltä päiviä sieltä sun kalenterista tehäksesi JCItä tai jotain muuta laatustandardii, koska se perustyö kärsii siitä. Niin se, että jos se on talolle tärkee juttu, ni siihen ehkä resurssoidaan sitten myös ihminen tekemään sitä. Joka ois, sitten vastaa tietyllä...

Haastattelija 1 [00:42:41]: Mut pitäiskö niinku sanoo, että pitäiskö se olla joka näissä ryhmässä erikseen se ihminen.

Puhuja 4 [00:42:45]: No niit tarvitaan varmasti niitä ryhmäihmisiä, mut siel pitää olla yks tai kaks, jotka kantaa vastuun. Et ei sielt esimerkiks mein henkilöstöpäällikkö kaiken oman työnsä ohella yrittää kasata tätä, niin eihän se, ei keltään, niinku. Ei vaan onnistu kenenkään oman työn ohella.

Puhuja 3 [00:43:01]: Tää on hyvä esimerkki nyt, kun mein pitää tehdä uus pelastussuunnitelma sairaalalle. Ja tääl oli palotarkastaja, joka anto rukkaset, kaikki, monille

osa-alueille. Ja sit se anto takarajan, et juhannukseen mennessä pitää olla pelastussuunnitelma tehtynä. Me todettiin, et ei kellään oo aikaa alkaa sitä niin paljon miettimään eri osa-alueita, et sit me palkattiin Securitaksesta konsultti tekemään sitä. Vetää kaikki langat yhteen ja osastot kyllä miettii keskenään sen, et miten, on niillä evakuointisuunnitelma siinä osastolla. Palo-ovien taakse ja, on se puol tuntii aikaa toisella palo-osastolla pitää potilaita, ennen kun ne pitää siirtää seuraavaks. Mut tää oli aika tärkee tää Securitaksen konsultti, joka on tehny tällasii muihin sairaaloihin ja tietää kaikki kaasuasioita, miten kaasut pitää hoitaa palotilanteessa ja miten palo-ovet pitää olla automaattisesti kiinni meneviä ja...

Haastattelija 1 [00:44:06]: Kyllä. No toi on just semmonen esimerkki, must hyvä esimerkki siitä, et kun tulee tommonen viranomaismääräys, et se on hoidettava kuntoon, ni sit se on vaan, niin kun se rahahan löytyy sillon jostakin. Niin se on vaan löydyttävä, koska muuten tää sairaala menee kiinni, jos tääl ei oo pelastussuunnitelma voimassa.

Puhuja 3 [00:44:21]: Niinpä. Ja nyt ens, ei kun siis tällä viikolla on nyt tota, se tapaa keskiviikkona, torstaina sit näit eri ryhmiä. Ja ne yhdessä laatii sit sen osaston pelastussuunnitelman valmiiks. Ei ois kyl ilman tämmöstä konsulttia varmaan menny läpi tässä ajassa. Riskinä ois ollu se, et palotarkastaja sanoo, että toiminta loppuu, ennen ku saa valmiiks. Ja sellasia on edelleen riskinä syksyllä, kun meillä varmaan tulee olemaan sitte harjotus, jota palotarkastaja tulee katsomaan. Ni sillon jos on pahoja puutteita, niin se voi antaa jonkun ukaasin ja sulkee jotain toimintoja, jos oikein mokataan. Sen takia se on ihan hyvä, et se nyt tulee tehdyksi.

Puhuja 4 [00:45:12]: Ni se on varmaan myös siin laatustandardissa tää.

Puhuja 3 [00:45:14]: Onhan se.

Puhuja 4 [00:45:15]: Yks osa-alue.

Puhuja 3 [00:45:17]: Kyllä.

Puhuja 4 [00:45:18]: Yks palikka on sit hoidettu. Mutta se, että jos aatellaan, et noinkin isoja kokonaisuuksia on monta.

Haastattelija 1 [00:45:22]: Niin mitkä on, puuttuu.

Puhuja 4 [00:45:24]: Niin.

Puhuja 3 [00:45:26]: Ja tossakin tulee mietittyä asioita, jotka on tiedetty, et on olemassa. Et esimerkiks, jos on osasto täys potilaita ja on vähän hoitajia töissä öisin, vartija on jossain, ties missä. Ehkä ei oo ees paikalla. Ni miten saadaan potilaat sit siirrettyä. Toivois, et koskaan ei semmost tilannetta tuu, mutta se pitää olla se suunnitelma ja sit kun on harjotus, niin periaatteessa se pitäis tehdä. Niillä hoitajilla, mitä on käytettävissä.

Haastattelija 1 [00:45:57]: Onneks nää harjotukset ei oo ikinä yöaikaan. Tai yleensä ikinä, että. Mut silti, onhan se ponnistus.

Puhuja 3 [00:46:08]: Ennalta jo tiedetään, et se on, aika, jos ois kuntoutusosasto ja vuodeosasto ois nelosella ois ihan täynnä, niin henkilökunta ei yöaikaan riittäis evakuoimaan. Tarpeeks nopeesti. Tai ainakin nyt alussa tuntuu siltä. Mut se selvii ens keskiviikkona ja torstaina, mitä pitää tehdä.

Haastattelija 1 [00:46:35]: Kyllä.

Puhuja 4 [00:46:35]: Mut se on askel eteenpäin. Sekin. Saadaan tehtyä, niin se on sitten raksi ruutuun.

Puhuja 3 [00:46:45]: Ja sitten, et lehdistöhän voi repii sit suuria otsikoita välillä tämmösistä, et täs sairaalas ei oo minkäänlaist pelastussuunnitelmaa tai.

Haastattelija 1 [00:46:53]: Niin ja varsinkin jos tulis oikeesti tulipalo, ni sitte kun huomattais, että ei, siihen ei kyetä.

Puhuja 3 [00:46:59]: Niin. Kyllä kaikki ymmärtää sen tärkeyden. Se pitää miettiä noi.

Puhuja 4 [00:47:04]: Ja sit se, et ko täs talos on niin monta toimijaa. Niin se, et jokainen on sitte, evakuoi samalla tavalla ja toimii samalla tavalla, et.

Puhuja 3 [00:47:11]: Joo.

Puhuja 4 [00:47:12]: Et sit kun palohälytin soi, niin mein asiakkaat raijataan pihalle ja (…) istuu tuolla odotustilassa. Niin se on kans niin kun, vähän se, että hoidetaanko me vaan omamme vai se tulee mukaan, että, taas kun kellot soi, että pitäs poistuu talosta. Niin sekin on sitte taas, niin kun sanoit, eka johto ois hallussa. Se on sitten niin kun omistajan taustalla vaikuttaa, kun pitäs olla yhteneväiset nekin.

Puhuja 3 [00:47:37]: Samanaikaisesti tämä omistaja tekee koko talon pelastussuunnitelmaa. Jossa, sit se yrittää koordinoida just nää eri toimijat yhteen. Ja myöskin sit jos yöaikaan nyt tapahtus tämmönen onnettomuus, niin pyritään niitä muita toimijoita käyttämään sitte vaikka apuna, jos sielt löytyy henkilökuntaa tai, riippuen vähän missä se palo sitten on. Et millä osastolla ja missä siivessä tässä talossa.

Haastattelija 1 [00:48:11]: Hyvä, mut hei kiitos teille. Tää oli tosi mielenkiintosta ja jotenkin, tää on aina hyvin silmiä avaavaa, et miltä se näyttää aina eri henkilöstöryhmiltä. Mut kiitos kun tulitte ja katotaan, mitä tässä nyt käy. Ihan niin kun, mitä se (…) päättää tän kanssa. Mut kiitos kovasti.

Puhuja 3 [00:48:30]: Kiitos.

Puhuja 1 [00:48:30]: Kiitos.

[recording ends]

Name of recording: Haastattelu_4_2022.mp3

Length of recording: 01:00:04

Information:

[?] = Word was not understood completely but meaning is almost correct. Recording point is written to the text e.g. [word? 00:15:44]

[??] = Word could not be understood and therefore could not be written. Recording point is written to the text e.g. [?? 00:15:44]

[text] = Sounds or not transcribed parts are written with square brackets e.g. [laughs] or [interview paused due to a phone call]

[recording starts]

Haastattelija 1 [00:00:03]: Nyt on nauha päällä.

Puhuja 1 [00:00:08]: Mä voisin kans sanoa, että viestinnän ja intranetin kautta olen osallistunut ainoastaan sillä tavalla että rakentanut sinne jonkunlaista intranet-sivustopohjaa, mutta mä oon ollut siinä uskossa et se koko hanke on nyt jotenkin jäissä, tai onko se edes edennyt, koska sinne intraan piti tulla niitä jotain hirvee kasa erilaisia tiedostoja, mutta ymmärtääkseni ne ei oo edennyt, tai en oo ainakaan saanut tietoa siitä. Mutta ei nyt mitenkään muulla tavalla ole ollut tässä myöskään mukana.

Haastattelija 1 [00:00:51]: Just näin. Eli sä (…) oot ollut silleen osallisena, et sä oot ollut siinä intran rakentamisessa, oisit pitänyt olla mukana, vai.

Puhuja 1 [00:01:02]: Siis minä olen tehnyt tälle JCI-porukalle tai laadunhallintateemalle. Mä oon meillä muutenkin se intran pääarkkitehti ollut, rakentanut sen Sharepoint-ympäristön meille, eli olen tehnyt sinne [sivupohjat? 00:01:17] ja jotain semmosta yleis diipa-daapa, tämmöstä juontoa sinne, alustusta. Ja tehnyt tavallaan niitä haluttuja kategorioita, siis tällasiin aihealueisiin, mihin oli kai tarkotus että sit tulee niitä selkeitä uusia ohjeita tai protokollia, että miten meillä toimitaan tässä ja tässä ja tässä asiassa, niin näille epäselvä, kaikuva niitä rakenteita tein. Mut se, että sinnekin piti sitten eri ihmisten, tässä laadunhallintahankkeessa mukana olevien ihmisten piti tuottaa materiaalia ja tuoda ne sitten sinne intraan, mutta koska sieltä ei ole mitään kuulunut et keiden kaikkien, ketkä tarviis oikeudet siihen, niin oletan että niitä tiedostoja ei olla vielä saatu valmiiksi tai ryhdytty tuomaan intraan.

Haastattelija 1 [00:02:15]: Just näin. Miten (…)? Säkö sanoit, et sä et oo ollenkaan osallistunut tähän?

Puhuja 2 [00:02:16]: En, mutta nyt kun mä ajattelen tätä, niin siellähän on paljon meillä tehty, niinkun... Ei varmaan intrassa, vaan ne on meidän omilla sivuilla.

Haastattelija 1 [00:02:27]: Tervetuloa.

Puhuja 3 [00:02:28]: Hyvää päivää.

Haastattelija 1 [00:02:29]: Hyvää päivää.

Puhuja 1 [00:02:30]: Päivää.

Puhuja 3 [00:02:32]: Anteeksi, että olen vähän myöhässä.

Haastattelija 1 [00:02:34]: Kiva, kun tulit. Mä oon siis (…) ja mä teen laadullista selvitystä siitä että miten tää laatujärjestelmä on teillä otettu käyttöön.

Puhuja 3 [00:02:44]: Hyvä. Minä olen [….].

Haastattelija 1 [00:02:47]: Kiva kun tulit.

Puhuja 3 [00:02:51]: Joo. Piti vielä saada leikkausosastolla kaikki semmoseen kuntoon että uskalsin tulla tänne.

Haastattelija 1 [00:02:59]: Kyllä. Asiat tärkeysjärjestykseen. Me oltiin puhumassa sitä laatujärjestelmää, että miten te ootte osallistunut sen laatujärjestelmän rakentamiseen. Just Päivi oli kertomassa jotain. Jatka vaan.

Puhuja 2 [00:03:08]: Joo, eli just sitä sanoin että en oo ollut niissä kun on kun on kutsuttu ihmisiä tekemään, niin en oo niissä ollut mukana, et sillä lailla vähän ulkopuolinen, mutta nyt kun mä mietin sitä meidän työtä, niin siellähän on tehty just palsepotilaiden, Heleniuksen Riinan ja Sari on tehnyt aika isonkin työn, et se on tavallaan laitettu ylös kaikke ne tärkeet asiat. Meistä pystyy aika monet tekemään nyt sitä, että sitä on niinkun ajatellut, kun täällä on aina tehty sitä [laatua? 00:03:36], niitä on laitettu ylös. Mä oon jotenkin ajatellut et se on ihan normaalia, mitä me normaalistikin tehdään, mutta itse asiassa sehän voi liittyä myös tähän, että mä en oo vaan sitä ajatellut sillä tavalla.

Haastattelija 1 [00:03:45]: Kyllä.

Puhuja 2 [00:03:46]: Tosi iso työhän siellä on tehty, et ne on kaikki laitettu ylös. Ja kun mä esimerkiks tein eri linjalla töitä, niin nyt sitten teen lauantaita osastolla, niin mulla on ollut ihan älyttömän helppoa sen pohjalta, kun mä kerkesin ne lukee ensin ite ja sit oon käynyt katsomassa muutamat ohjaukset ja oon pystynyt tuuraamaan jos joku on poissa. Et siinähän se kyllä näkyy, et varmaan ehkä se on liittynyt jotenkin tähän.

Haastattelija 1 [00:04:08]: Entäs (…)? Näkyykö sun työssä? Miten sä oot osallistunut tähän laatujärjestelmän rakentamiseen, vai ootko osallistunut?

Puhuja 3 [00:04:17]: Tarkoitatko nyt siitä lähtien kun meille tuli tämä...

Haastattelija 1 [00:04:20]: JCI.

Puhuja 3 [00:04:21]: Idea, että JCI?

Haastattelija 1 [00:04:25]: Kyllä.

Puhuja 3 [00:04:26]: No, mut vähän niinkun nakitettiin siihen alusta lähtien, että kun oli tuolla ensimmäinen koulutustilaisuus Haagassa jossakin hotellissa, Jenkeistä oli tullut siihen JCI:n edustaja. Sit mut valittiin siihen, että (…), mene sinä sinne mukaan. Sillon oli vielä ihan semmonen rivityöntekijä, että en vielä ollut (…) silloin. Eli siitä lähtien olen ollut kyllä mukana ja sitten meillä oli yksi, tai kun siihen JCI:n kuuluu näitä työryhmiä, minä olin vähän niinkun se sihteeri siinä ASC-ryhmässä, eli onks se anestesia and surgery - jotakin. Mikä se C nyt olikaan? Apua. Mutta siinä ryhmässä olen ollut sitten mukana ja istunut melkein kaikissa näissä JCI:n kokouksissa.

Haastattelija 1 [00:05:51]: Miten sä oot kokenut ne kokoukset ja koulutukset ja sen orientaation siihen JCI:n?

Puhuja 3 [00:06:02]: Vaihtelevaa ollut. Vaihtelevana olen kokenut. Se ihan se ensimmäinen, monen päivän koulutus oli tietysti semmonen hyvin strukturoitu paketti ja kyllä sai aika

hyvän käsityksen siitä mitä haetaan ja miten myös laaja se on. Ja sitten meidän ensimmäiset yhteiset JCI-palaverit olivat myös mielestäni ihan OK vielä. Ja sitten alkoi meidän alaryhmän kokoukset ja siinäkin alussa mielestäni päästiin eteenpäin. Saimme aikaan semmosia alustavia tekstejä ja... Mutta sitten tapahtui nitkahdus aika monella tavalla.

Haastattelija 1 [00:07:17]: Mistä se johtu?

Puhuja 3 [00:07:20]: Mulla on muutama idea, mutta en nyt osaa sanoa että miten paljon mikäkin tekijä on vaikuttanut. Yksi on ollut se, että mä jouduin, tai minusta tuli (…) ja sit minulle tuli paljon enemmän tavallaan vastuita, niin että semmoseen taas ei ollut ehkä niin paljon aikaa ja energiaa. Sitten...

Haastattelija 1 [00:07:56]: Mut oliks se sitten sun vastuulla siihen asti, et sä sitä veit eteenpäin alaryhmässä?

Puhuja 3 [00:08:01]: Kyllä, näin voi sanoa.

Haastattelija 1 [00:08:05]: Niin, eli sä rupesit tekemään enemmän (…) hommia, niin sitten kukaan ei ottanut sitä vastuuta.

Puhuja 3 [00:08:10]: Joo, näin voi sanoa. Näin mä olen kokenut, että mä olin se vastuuhenkilö siinä meidän alaryhmässä. Ja alussa oli ehkä myös vielä [suht koht? 00:08:25] helppo saada alaryhmän edustajia mukaan, mutta sitten oli myös vaikeampi ja vaikeampi löytää yhteisiä hetkiä. Sitten tuli korona, et se kokoontuminen oli sitten... No, osittain mahdoton ja kielletty, jopa. Sitten jos tämmöstä yrittää hoitaa vain netin kautta, se on kyllä hankalaa. Ja sitten mä... Se on nyt mulle henkilökohtainen mielipide. Suurin ongelma on ollut alusta lähtien että meillä ei ollut projektipäällikköä. Mä, kun oli Haagassa se koulutus, mä kyllä otin asian puheeksi ja ehdotin, yritin siihen suuntaan saada et tulis semmonen, mutta siihen ei menty. En mä tiedä oliko se rahakysymys. Haluttiin ehkä säästää semmosta palkkaa, palkkiota siinä hetkellä ja sitten talon johdolla oli se ajatus että tää hoidetaan semmosena että johtaminen jaetaan muutamalle henkilölle, eli siinä oli (…), meidän koko firman toimitus... Anteeksi, ei toimitusjohtaja vaan lääketieteellinen johtaja. Sitten (…). Apua, nyt sukunimi puuttuu, joka oli erittäin tärkessä roolissa siinä mut hän lähti äitiyslomalle. Ja sitten kolmantena (…), joka on sen tutkimusyksikön vastuuhenkilö. Ja kyllä, alussa oli se ehkä myös sujunut, mutta sit kun (…) lähti pois se vähän niinkun rakoili. Ja... No, (…) on nyt lähdössä eläkkeelle ja (…) varmasti on yrittänyt parhaansa, tavallaan ottaa kopin siitä, mutta... Mä luulen, että se on se suurin ongelma. Ja sit, jossain vaiheessa tuli vielä noi (…) aikeet mukaan ja myös koko (…) nyt otetaan se JCI ja sitten sinne satsattiin

niinkun (…) on tapana - tehdään isosti. Siellä oli sitten erilaisia koulutuksia ja palavereita ja webinaarejakin ja ihan hyvä näin. Ja sit ajateltiin, et me saadaan sieltä jotakin hyviä ideoita, mut se mielestäni ei kuitenkaan... Ei syntynyt semmosta synergiaa, tai en... Ainakin henkilökohtaisesti en kokenut että siitä olis ollut meillä hyötyä. Ja nyt mä kuulin, että (…) harkitsee [luovuttamista? 00:12:25]. Mä en tiedä onks tämmönen teilläkin tiedossa, mutta olet kuullut myös sellasta.

Haastattelija 1 [00:12:30]: Oon. Mä oon kuullut sen. Mä oon [?? 00:12:33].

Puhuja 3 [00:12:37]: Joo.

Puhuja 1 [00:12:36]: Mä voisin täältä kompata (…), vaikka en oo itse ollut näissä kriittiseltä kannalta mukana tässä, ainoastaan sitä intrahommaa rakennellut ja siinä jonkun verran tehnyt (…) ja (…) kanssa yhteistyötä, niin on kumminkin jäänyt semmonen kuva että jos ei tämmöseen näinkin isoon hankkeeseen, jotta se saatais vietyä tehokkaasti läpi, jos ei siihen allokoi tätä resurssia, elikkä sit sitä projektipäällikköä tai selkeesti sitten vähennetä niiltä vastuuihmisiltä muita töitä pois, niin eihän se... Se jää niinkun aina sit vähän silleen, et sit kun keretään ja nyt otetaan aikalisä ja näin. Jää nyt semmonen mielikuva tästä sivusta katsellessa, että moni asia venyy sen takia että kun on niin paljon kaikkea muuta, et ihmiset puuhastelee ja tekee jos jonkinlaisia asioita, niin se on ehkä yks miinuspuoli. Sitten toinen tuli, tässä samalla kävin katsomassa sitä intrasivua minkä mä nyt oon rakentanut siihen, niin kiinnitin nyt vaan huomiota kun tässä on nää aihealueet, niin kumminkin jos puhutaan potilaiden kokemasta laadusta, niin minun mielestä sen laaduntarkastelun pitäis kattaa ihan se koko ketju, alkaen siitä potilaan ensimmäisestä yhteydenotosta. Tästä esimerkiksi toi asiakaspalveluprosessi näiden otsikoiden valossa näyttäisi puuttuvan, et mikä on se asiakaspalvelun rooli eri vaiheissa. Kaikki ne kontaktit mitkä siinä kohtaa syntyi, ennen kuin potilas todella on siellä leikkauspöydällä tai vuodeosastolla tai kuntoutumassa, niin tämmönen huomio.

Haastattelija 1 [00:14:44]: Sillon tätä JCI:tä oltiin ottamassa teille käyttöön, niin minkälaisia odotuksia teillä oli? Oliks teillä kovat odotukset? Miks te rupesitte tähän hommaan? Oliks esimerkiks joku mahtikäsky ylhäältä että oli pakko? Ajattelitteko te ite, että siitä olis hyötyä?

Puhuja 3 [00:15:07]: No, kyllä siihen viimeiseen voi vastata kyllä.

Haastattelija 1 [00:15:11]: Niin, minkälaisia odotuksia?

Puhuja 3 [00:15:14]: Ehdottomasti hyötyä, että saa tämmösiä prosessikuvauksia paperille, että kuvataan sitä hyvää työtä mitä me kuitenkin tehdään ja sitä voi käyttää hyväksi kun tulee uusi työntekijä. Voi näyttää että näin meillä on tapana. Ja sitten samalla kun niitä laatii huomaa ehkä myös kohtia missä on kitkaa, missä toimintatavat ovat eriävät. Sit voi yhdessä miettiä, että minkä takia ja olisiko ehkä potilasturvallisuuden näkökulmasta parempi et me yhtenäistetään tai hakee sitä uutta tietoa että mikä on nykyisin ehkä se paras tutkimustulosten valossa. Ja sitten kun lääketiede menee eteenpäin ja joku hoitomenetelmä, tapa muuttuu, sit se on myös helppo muuttaa siinä jo olemassaolevassa ohjeessa. Et kyllä tämmösiä tavallaan toivottiin ja semmosesta... Kyllä semmosia potentiaalisia hyötyjä tässä on varmasti.

Haastattelija 1 [00:17:06]: (…) sanoitkin tossa kun sä olit mennyt uuteen tehtävään, niin se oli helppo kun oli olemassa jo ne.

Puhuja 2 [00:17:11]: Kyllä.

Haastattelija 1 [00:17:12]: Haluatko kertoa sitä enemmän?

Puhuja 2 [00:17:15]: No, tavallaan meillähän on kyllä pyritty tekemään aika paljon aikasemminkin vuosien aikana, silleen että on pyritty laittamaan papereihin raamia et miten jotkut asiat menee, tai vaikka ryhmien sisältö, et mikä vois olla semmonen, et jos joku joutuu tuuraa yks-kaks, niin se helpottaa hirveesti et se pystyy tutustuu etukäteen. Et sitä on kyllä tehty ja silleen, mä niinkun... Siinä vaiheessa kun tää tuli, et en ite oo siinä ollut mukana, mutta sehän kuulosti tosi hienolle ja mä sillon ajattelin että paljonhan tätä on tehty jo nyt, että on tavallaan, että se ei oo sillä lailla uutta, mutta se kuulosti kyllä tosi isolta ja laajalta ja tosi iso työhän siinä on, että sillon mietin just että miten se mahtaa onnistua sit, että mistä siihen otetaan se aika. Kuitenkin tietää, että on aika hektistä välillä kaikilla varmasti, niin, tota...

Haastattelija 1 [00:18:03]: Koitteks te sillon alussa kun tätä otettiin käyttöön, että siitä ois jotakin muuta haittaa kuin se aikameno? Näitteks te nurjia puolia tässä? Huonoja puolia?

Puhuja 1 [00:18:20]: Vaikee ottaa minun mitään kantaa tohon, koska oma rooli on ollut täällä enemmän tän pelkästään tän intranet-ympäristön ja myöskin ehkä viestinnän kannalta, et jos ois jotain kivaa, uutta kerrottavaa tästä JCI:stä, et missä mennään, et olemme nyt tässä vaiheessa ja millon käyttöönotto ja mitä tää nyt oikeesti tarkottaa, et mä mielelläni viestijänä, viestinnän ammattilaisena tekisin vaikka intraan sit uutisia tästä ja ehkä

ulospäinkin, kun me oltais tää hienosti lanseerattu. Mutta ei mulla muuta. Samalla kysyn... Mä huvikseen kokeilin tossa jakaa näyttöä. Näkyyks tää intran sivu?

Haastattelija 1 [00:19:06]: Hyvin näkyy.

Puhuja 3 [00:19:11]: Näkyy.

Puhuja 1 [00:19:13]: Eli tätä tarkotin, kun mä katoin tässä näitä otsikoita et mitä kaikkee tässä otsikkotasolla on, niin mun mielestä tästä puuttuu se asiakaspalvelu. Se on osa sitä kokonaista potilaan palveluketjua. Ja koska tiedän että siellä asiakaspalvelussa on erinäisiä ongelmia jotka varmasti välittyy myös esimerkiks puhelinpalveluissa sitten potilaalle tai tällä tavalla, niin olis hyvä että sekin huomioitais sitten. Ja sitten tommonen niinkun perehdytyspuoli sitten.

Haastattelija 1 [00:20:03]: Puhuit (…) tossa aikasemin sitä, että kun saadaan ne yhteiset ohjeistukset ja sitten tiedetään paremmin, et sitten se toiminnan laatukin tai potilaan kohtaamisen laatukin siinä paranee, niin haluaisitko jatkaa siitä. Onko sulla siitä enemmän näkemystä, että miten se vaikuttaa eri tavalla siihen toiminnan laatuun? Puhuit siitä potilasturvallisuudestakin, et kun ihmiset tietää mitä tekee, kaikki tekee suurin piirtein samalla tavalla, uusimpien lääketieteen tutkimusten mukaisesti.

Puhuja 1 [00:20:36]: Kysyiks sä multa vai (…)?

Haastattelija 1 [00:20:39]: Mä ajattelin, et jos (…) aloitti tosta vähän.

Puhuja 3 [00:20:42]: Mä en ymmärtänyt kysymystä.

Haastattelija 1 [00:20:45]: Puhuit äsken siitä, että kun jos huomataan semmosia kohtia missä toimintatavat on erilaisia ja sitten kun ne saadaan yhdistettyä, yhtenäiset ohjeet, niin sitten onks siinä... Sittenhän siinä luulisi olevan myönteistä seurausta siihen potilaan kohtaamiseen ja potilasturvallisuuteen.

Puhuja 3 [00:21:06]: Joo, siis jos...

Haastattelija 1 [00:21:08]: Ja sitä kautta se toiminnan laatu paranisi, tai jollakin tavalla ainakin yhtenäistyisi.

Puhuja 3 [00:21:18]: Joo. Kyllä ehdottomasti uskon tähän ja mulla on nyt myös muutama esimerkki tavallaan mielessä missä me olemme tavallaan tällä tavalla myös lisänneet potilasturvallisuutta.

Haastattelija 1 [00:21:45]: Haluuks sä kertoa niitä?

Puhuja 3 [00:21:48]: Yks on toi, kun yleisanestesian yhteydessä annetaan lihasrelaksanttia, ihan Suomen Anestesiayhdistykseltä löytyy semmonen selkeä ohje, että jos käytetään niin sitten täytyy olla myös senmukainen monitorointi. Eli sitä lihasten relaksaation astetta pitäis ihan mitata laitteella, seurantalaitteella. Jos esimerkiksi ennen herätystä potilas on vielä liian relaksoitunut, sit pitäis antaa relaksantin vasta-aineita potilaalle, ennen kuin herättää potilaan. Ja siinä meillä oli selvästi erilaisia tapoja sekä anestesialääkäreiden että hoitajien osalta. Ja kun tää asia tuli tavallaan nyt esiin, tai asia joka on ollut tavallaan tiedossa, mut kun nyt tämän JCI:n takia siihen jouduttiin kiinnittämään huomiota, sitten tavallaan tuli myös se oivallus että näin ei sais olla. Ja sitten laadittiin ohje siitä ja esitettiin osastotunnilla ja siitä lähtien meillä on kaikilla... Kaikki hoitavat nyt samalla tavalla. Se on hyvin, hyvin konkreettinen, tää esimerkki.

Haastattelija 1 [00:23:54]: Kyllä, h yvä. Mitä muuta te ajattelitte, minkälaisia muita muutoksia teillä arjen työssä on ollut tän JCI:n takia? Toi oli musta tosi hyvä esimerkki tuo, mutta onks muita tommosia konkreettisia muutoksia. Sä sanoit että kun meet uudelle osastolle niin tiedät mitä tehdä siellä, kun siellä on ne olemassa.

Puhuja 2 [00:24:14]: Ehkä just se mitä mä oon tehnyt pitkään kipuasiakkaiden kanssa, missä ei ehkä ole sellaista että mennään tietyn protokollan mukaan, vaan jokainen on niin yksilö ja joutuu hirveen paljon aina miettimään että mitä tän kohdalla, mistä lähdetään eteenpäin, niin mä oon kokenut ihan hirveen hyvänä ja itelleni tavallaan työssä jaksamiseen vaikuttavana sen, että mä oon pystynyt välillä tekemään semmosta missä on selkeä protokolla ja [jos tarvii? 00:24:37] mä pidän vaikka jonkun pre-operatiivisen ryhmän. Mä tiedän tasan tarkkaan mitkä on ne asiat. Tavallaan mä pystyn olemaan läsnä niille asiakkaille, ehkä siihen mitä (…) tuossa sitä laatua niinkun ihmisille, että he pystyy kysymään ja on, niinkun... Että se on tavallaan sillä lailla helpompaakin kun siinä on joku protokolla.

Haastattelija 1 [00:24:56]: Kyllä, voi saan seurata sitä eikä tarvii keksiä.

Puhuja 2 [00:25:00]: Niin, niin siihen omaan työssä jaksamiseen aika iso merkitys kyllä.

Haastattelija 1 [00:25:06]: Mitä te muuten ajattelette, kun puhuitte tossa työssä jaksamisesta tai työhyvinvoinnista, niin miten te näätte sen työssä jaksamisen tai työhyvinvoinnin yhteyden tähän JCI:n tai laatujärjestelmään?

Puhuja 3 [00:25:25]: Siis tarkotatko lisääkö laatujärjestelmä ja sen läpivienti...

Haastattelija 1 [00:25:34]: Työtä, vai helpottaako se sitä?

Puhuja 3 [00:25:42]: Joo. No, kyllä se jollain tavalla tekee työstä myös helpomman, että kun on sovitut tavat. Ja...

Haastattelija 1 [00:25:59]: Mihin asioihin siihen työhyvinvointiin tällä laatujärjestelmällä teidän mielestä on eniten vaikutusta, kun ajatellaan sitten kun tää olis valmis?

Puhuja 1 [00:26:14]: No, mulle, jos täältä linjoilta huutelen... Katotaas tota listaa tossa noista osa-alueista, niin ihan kiinnostais tietää mikä tää hallintojohtajuus ja ohjaus, et kun jos on jossain päin tiettyjä ongelmia... Siellä asiakaspalvelussa tiedän että erinäistä ongelmaa on ollut, niin kyllä mua ihmetyttää johtajuuden kannalta että miksi ei sit mennä juurisyihin, et jos me... Sanon nyt tässä. Täähän on luottamuksellista.

Haastattelija 1 [00:26:52]: Tää on luottamuksellista, niin.

Puhuja 1 [00:26:56]: Olen markkinointihenkilönä ja viestijänä joutunut törmäämään siihen, että meillä jatkuvasti lähtee asiakaspalvelusta uusia työntekijöitä pois. Osa ei jaksa kun viikon pari ja taas vaihtuu, eli mä saan tehdä niitä rekryilmoituksia someen ja meidän nettisivuille tuon tuosta ja tästä. Tietenkin päättelen että jokuhan siellä mättää, jos ihminen ei siellä jaksa olla, lähtee heti pois. Ja sitten kuuluu sellasia huhuja. Nää on tietysti tällasia osin huhupuheita, mutta että ei ole kunnollista perehdytystä tai on töykeää kohtelua ja oletetaan että uuden ihmisen pitäis osata. Vaikka tää ei millään tavalla minun työhyvinvointiin liity, mutta tavallaan kuitenkin välillisesti ärsyttää. Siis meillä on selkeesti ongelma siellä jonkun tason johtamisessa, että miksi ei sitten, niinkun... Eihän siinä oo mitään järkeä että jatkuvasti vaan että no, pannaan uutta hakuilmoitusta tulemaan ja

rekrytoidaan sitten uudestaan - katotaan miten niiden kans käy. Että pitäis miettiä niitä juurisyitä, että miten me voisimme parantaa tätä toimintaa täällä asiakaspalvelussa, miten työilmapiiriä, uuden työntekijän perehdytystä, niin et kaikilla olis hyvä olla. Et kyllä minun mielestäni myös tämä on osa sitä laadukasta toimintaa ja tavalla tai toisella se välittyy meidän asiakkaille. Eli tämä vaan huomiona tuohon kohtaan hallintojohtajuus ja -ohjaus, et mitäköhän siellä osa-alueella on sitten niinkun meneillään.

Puhuja 3 [00:28:45]: Oikein hyvä kommentti ja esimerkki. Kiitos.

Haastattelija 1 [00:28:55]: Onko teillä jotain perehdytys... Niinkun eri työtehtäviin minkäännäköistä perehdytysmateriaalia ollut?

Puhuja 2 [00:29:01]: Meillä on kyllä ollut, laajatkin, jo sillon kun mä oon tullut 20 vuotta...

Haastattelija 1 [00:29:06]: Niin, mut asiakaspalvelussa selkeästi ei vaikuta olevan, tai sit sitä ei käytetä.

Puhuja 1 [00:29:11]: Vaikee ottaa kantaa.

Puhuja 3 [00:29:14]: Joo, en osaa sanoa.

Puhuja 1 [00:29:15]: Tiedä onko siellä mitään tai eikö ole, että miten se menee.

Puhuja 3 [00:29:21]: Jos ajattelen omaa esihenkilöasemaa, tarkoittaa uusien anestesialääkäreiden perehdytystä ja ei ole mitään semmoista mustavalkoisena, mielestäni. Se tulee vähän sitten tavallaan selkäytimestä, että tuut nyt mun kanssa tänään ja sit mä näytän sulle paikat ja jutellaan ihmisten kanssa, työntekijöiden kanssa jotka ovat eri pisteissä. Muutama esimerkki, potilas ja tietotekniikka vähän, toi akut ja sairauskertomusohjelma. Ja jos on sitten kysymyksiä, tulee kysymään. Ja sit tietysti voi viitata niihin jo olemassaoleviin ohjeisiin ja että kyllä, onhan meillä joitakin tämmösiä esilääkitykseen liittyvää, tiettyjä potilasryhmiä koskien, päiväkirurgisia potilaita, kipulääkitykseen liittyen leikkauksen jälkeen. Tämmösiä ohjeita kyllä löytyy erilaisia ja eri vuosilta ja ajalta ennen JCI:tä, että tavallaan emme lähteneet ihan nollasta kun JCI-projekti tuli. Mutta siis konkreettisesti anestesialääkäreiden perehdytykseen ei ole olemassa mitään.

Haastattelija 1 [00:31:43]: Pitäiskö sun mielestä olla?

Puhuja 3 [00:31:47]: Jos nyt vastaan kyllä, sitten...

Haastattelija 1 [00:31:54]: Sit saat ite tehdä, niinkö?

Puhuja 3 [00:31:57]: Olen itse siinä hirressä, että... Joo. No joo, siis vaikea sanoa. Me olemme tietysti koko hoitoalalla vähän tämmönen siinä pyramidissa ehkä aika korkeassa päässä, eli meille ei tuu nyt ihan tämmönen joka on just tullut koulun penkiltä, tai et kyllä kollegat jotka tänne tulee, hänellä on jotakin myös kliinisen työn taustaa ja et se on... Joo. Hyvä olis varmasti joku... Joku ainakin tämmönen lyhyt lista, ranskalaiset viivat, sillä periaatteella et ne varmasti ne tärkeimmät asiat tulevat puheeksi.

Puhuja 1 [00:33:11]: Niin, semmonen otsikkotason perehdytyslista joka toimii muistilappuna perehdyttäjälle, olit se sitten sinä ylilääkärinä tai joku kollega sieltä.

Puhuja 3 [00:33:24]: Joo.

Puhuja 1 [00:33:24]: Et ihan vaan otsikkotasolla ja sit rasti ruutuun, että nää on käyty läpi, nää on käyty läpi.

Puhuja 3 [00:33:32]: Joo.

Puhuja 1 [00:33:33]: Ja myöskin ei vaan siihen kliiniseen anestesitoimintaan, mutta myöskin semmonen että sitten mitä on meillä työntekijänä oleminen yleisesti ottaen, niin olis hyvä olla siinä.

Puhuja 3 [00:33:45]: Joo, hyvä. Kiitoksia. Tässä itse asiassa tulee mieleen, kun mä sain reilu vuosi sitten perehtyä [Olof Kroneen? 00:33:55] ja silloin työnjohdolta tuli, tai siihen löytyi jotenkin netistä, intrasta joku tämmönen yleinen lista, että liittyen muun muassa siihen mistä saa avaimet. Työterveyshuoltoon, palkkatoimistoon. Täytyykö laittaa sinne verokorttia vai ei? Että tämmönen lista kyllä on olemassa ja olen sitä myös käynyt läpi Olofin kanssa. Eli sellanen yleisellä tasolla, mut se soveltuu jokaiseen työntekijään, että ei ollut anestesialääkärin spesifisti. Joo, hyvä lisäys.

Puhuja 1 [00:35:02]: Niin, tommosenhan pitäis tulla sieltä HR-puolelta, et ne yleiset työntekijyyteen liittyvät asiat, niin ne on tämmösenä check listana, että kaikki on käyty läpi. Ja sit jokainen esimies voi niitä soveltaa sen lisäks että perehdyttää niihin omiin substanssikohtaisiin asioihin.

Puhuja 3 [00:35:23]: Joo.

Puhuja 2 [00:35:27]: Sillonhan täällä oli kun mä oon tullut tänne, niin sillonhan meillä oli semmonen lista just missä luki kaikki ne, et se jäi itselle se nivaska. Ja oli myös semmonen perehdytyspäivä kaikille ketä oli uusia työntekijöitä, niin oli sitten, että oliks se puoli päivää tai jotain. Sitä mä en enää muista, mutta tohon mä voisin fysioterapian puolelta, kun mehän tehtiin aikaisemmin Kelan kuntoutusta, niin meillähän sillon Kela edellytti että meillä piti olla kaikki ne työnkuvaukset kirjotettuna auki tosi tarkasti ja ne teki auditointeja. Kipukuntoutusauditointi ja yksilökuntoutus, muistaakseni. Ne oli aika tosi tiukkoja ne, niin on jouduttu tekemään sitä jo tosi kauan sitten ja se on ehkä pohjana ollut nytkin, että ne on... Edelleen sieltä on jäänyt sitä, et vaikkei oo enää Kelan kuntoutusta, mut ne tietyt mallit on jäänyt niinkun jokaiselle päähän,et tekee... Et siitä on ollut kyllä iso apu, vaikka ne oli sillon tosi työläitä.

Haastattelija 1 [00:36:21]: Eikö teillä enää oo sellasia yhteisiä perehdytyspäiviä, kun tulee uus työntekijä?

Puhuja 2 [00:36:21]: Sillon ehkä tuli enemmän niitä. Nyt ei varmaan tuu niin paljon.

Puhuja 3 [00:36:26]: En ole kuullut semmosesta. Mut mä voin vielä lisätä, kun meillä on myös tämmösenä pakollisena tehtävänä uuden lääkehoitosuunnitelman laatiminen, luominen, ja siihen on myös oma työryhmä jossa mä olen myös silloin tällöin ollut mukana ja nyt mut on pyydetty siihen mukaan. Siinä puhutaan myös uusien työntekijöiden perehdytyksestä ja siinä myös tavallaanon se ehdotus että olis tämmönen ihan perehdytyslista, tai tämmönen muistilappulista. Ja mä tiedän, että anestesiahoitajille semmonen on jo luotu. Instrumenttihoitajille se on työn alla ja kuka oli sit se kolmas. Oliks se kipupoliklinikka, mut siis mä en mee vannomaan. Mut semmosia on nyt tavallaan työn alla ja... Eli vaikka se ei tullut JCI:n laatujärjestelmän suunnasta, se on kuitenkin asia jossa sitten on tämmösiä päällekkäisyyksiä ja myös synergiaa, että kun lääkehoitosuunnitelman nimessä luodaan, sitä voi hyödyntää myös suhteessa yksi-yksi JCI:ssä, tai vastaavanlaisessa. Joo.

Haastattelija 1 [00:38:45]: Jos mietitte sitä, tai varsinkin (…), sitä leikkaussalityöskentelyä, niin onko leikkaussalissa sinällään vaikuttanut tää laatujärjestelmä tai ohjeistukset mitenkään siihen työskentelyyn. Onko ollut vaikutusta mihinkään leikkausaikoihin tai mihinkään potilasturvallisuuteen tai muuten sen potilaan hoitoon liittyviin asioihin?

Puhuja 3 [00:39:15]: No, yhden esimerkin mä jo kerroin. Se oli se...

Haastattelija 1 [00:39:23]: Lihasrelaksantti. Joo.

Puhuja 3 [00:39:24]: Joo. Sitten... No, sillä leikkausosastolla oli tapahtunut semmonen mielestäni aivan käsittämätön kehitys, että ei pidetty enää osastotunteja leikkausosastolla, operatiivisella puolella, missä tehdään todella monimutkaisia asioita. Ja sitten ei enää ollut osastotunteja. Musta se on aivan käsittämätön asia. Aika pitkän väännön jälkeen me saatiin siihen osastotunti takaisin, että kerran kahdessa viikossa. Se on mielestäni työn laadun ja potilasturvallisuuden osalta erittäin tärkeä asia ja se kuuluis varmasti johonkin näistä. Mutta en nyt... Mulla on... Joo, en mä tiedä, se on liioiteltua sanoa että se oli nyt JCI-projektin ansio että se tuli takaisin, mutta se oli ehkä enemmän semmonen sitten... Johdon piti sitten myöntää, että jos meillä ei oo säännöllisesti osastotuntia emme koskaan saa JCI-sertifikaattia. Se oli ehkä enemmän semmonen mekanismi. Jos saat kiinni siitä.

Haastattelija 1 [00:41:40]: Kyllä. Miks sä uskot, että se osastotunti oli otettu pois? Oliko se ihan säästötoimenpide, vai? Miks se oli lakkautettu?

Puhuja 3 [00:41:52]: Siihen vaikutti m oni asia. Ensin meillä oli se säätiö taustana, mut sitten meistä tuli privaatti ja sitten ehkä enemmän tämmösiä... Kiinnitti enemmän huomiota semmosiin asioihin että täytyy olla tehokas ja täytyy olla kustannustehokas ja sitten taloudelliset paineet, koko maan talous vaikeuksissa ja sitten säästetään sieltä täältä ja sitten... No, ehkä sit on tärkeämpää että aloitetaan aina aamulla heti kello kahdeksan ja tehtiin semmosia sopimuksia myös sitten joista jätettiin tää mahdollinen osastotunti pois ja sitten kun tuli valituksia, sit sanottiin että joo, mutta näissä sopimuksissa lukee että täytyy aloittaa aamulla kello kahdeksan. Ja ehkä ei myöskään uskallettu sitten näissä neuvotteluissa vaatia, että... Että kyllä me tehdään täällä leikkauksia, mutta kerran viikossa meillä on ensin puoli tuntia [osasto poikki? 00:44:01]. Se oli liian... Ei uskallettu sanoa ääneen sille joka mahdollisesti maksaa meille sitten sopimuksesta. Ja sitten ehkä ei myöskään ymmärretty miten tärkeää se koulutus on, että... Ja mulla oli pitkään semmonen että ensin täytyi tapahtua jotakin pahaa ennen kuin sit myös tätä ymmärretään laajemmalla. No, onneksi meille ei oo tapahtunut mitään katastrofia, tai... Mut... Joo, onneksi semmoselta on vältytty. Ja sitten... No, johdossa on koko ajan tapahtunut muutoksia. Ylilääkäri... Siis toimitusjohtaja on muuttunut vuosien varrella. Operatiivisen puolen ylilääkäri on vaihtunut.

HR on vaihtunut. Leikkausosastolla on kaks kertaa ylihoitaja vaihtunut ja se joka oli siinä välissä lyhyen ajan, hän ei... Hän ei tavallaan... Hänellä oli niin paljon muita juttuja ja ongelmia, että hänelle se osastotunti tuli ihan semmosena, hännässä viimeisenä, ja sitten... No, hän ei tavallaan ollut sittenkään pitkään meillä, erilaisista syistä, että se oli hänelle ehkä liian iso pala ja liian paljon uusia asioita kerrallaan. Ja sit tuli se nykyinen ylihoitaja, joka tulee talon sisältä ja tuntee kaiken sillä lailla todella hyvin, mutta jostakin syystä hänelläkään se osastotunti ei ollut sillä lailla tärkeänä. Et tämmösiä tekijöitä. Ja sit kun se oli kerran tippunut pois, yli vuosi siihen meni et saatiin se takaisin.

Haastattelija 1 [00:46:57]: Okei. Oliko (…) tai (…) vastaavia kokemuksia, tai miten te ajattelette et se on vaikuttanut toi laatujärjestelmä johonkin?

Puhuja 2 [00:47:12]: En osaa kyllä nyt silleen sanoa. Sen verran vähän ehkä se on kuitenkin näkynyt meidän työssä tuolla.

Haastattelija 1 [00:47:17]: Onks (…) lisättävää?

Puhuja 1 [00:47:21]: No, eipä oikeestaan, että niinkun sanoin, kun on tällä tavalla vähän eri näkökulmalta täällä sivusta puuhastellut tän intran kanssa, niin eihän mulla noihin substanssijuttuihin oo mitään muuta kun mitä nyt tässä on näitä huomioita kertonut.

Haastattelija 1 [00:47:45]: Hyvä, nekin on tärkeitä. Miten te ajattelette, että laatujärjestelmä vaikuttaa sairaalan tulevaisuuteen? Oli sitten täällä...

Puhuja 1 [00:47:51]: Saanko yhden jutun?

Haastattelija 1 [00:47:53]: Sano toki.

Puhuja 1 [00:47:57]: Tää on vaan jälleen kerran tämmönen huomio, että minun mielestä kun tämmönen iso hanke on tekeillä ja en tiedä miten se nyt juuri tällä hetkellä käytännössä siellä etenee, tai eteneekö. Juuri katsoin tässä intrassa, et eihän näihin lokeroihin mitä täällä on tätä ohjeistusta... Tässä näätte, että me jaettiin ne tämmösiin kategorioihin, niin eihän tänne ole mitään tullut. Hirmu kiire oli saada tää sivusto tähän, mutta sit se on jämähtänyt tähän. Niin, et viestinnän ammattilaisena minusta aina tämmösistä hankkeista, jos halutaan sellasta henkilöstön sitoutumista... Nythän nykyään puhutaan tämmösestä employee engagementista, halutaan sitouttaa ihmisiä ja innostaa, niin pitäis tasaisesti, pitkin matkaa

pystyä kertomaan että me ollaan tällä polulla ja missä nyt ollaan, mihin ollaan menossa, mitä ollaan saatu aikaan. Että itse olisin, niinkun sanoin, kiinnostunut viestimään tästä juuri tältä kantilta että miten tää etenee, niin ei oikein mitään kättä pidempää oo tullut. Oon sitä nyt kerran-pari ehkä kysäissyt, että olisko jotain väliaikakerrottavaa tässä, mistä vois ihan pienenkin intrauutisen tehdä, niin se vastaus on aina vähän se että no, tää on nyt vähän jäissä kun tässä ei oo nyt aikaa ollut.

Haastattelija 1 [00:49:24]: Niin.

Puhuja 3 [00:49:26]: Siis, ei ole nyt kaikkia laittaa koronan tavallaan... Tai ei voi syytää koronaa kaikesta, mutta täytyy nyt olla myös sillä lailla lempeä, että kyllähän tämä koronapandemia on sekoittanut paketit niin, niin paljon ja oli tämmösiä, että... Että sairastumisten takia piti laittaa leikkauslistaa ja kaikki aikataulut uusiksi ja siirtää ja joustaa ja toivoa että firmat pysyy pystyssä, koska olihan tää nyt myös taloudellisesti semmoselle pienelle firmalle kuin me ollaan todella suuri riskiaika. Ja kaikki se stressi joka siihen liittyy, se et jokaisella työntekijällä sit tämmösiä varmasti tämmösiä pelkoja, että mihin tää johtaa ja milloin laiva uppoaa ja... Et kyllä sillä... Ja puhun nyt myös itsestäni. Kyllä, onhan tää myös vienyt energiaa ja jaksamista, että vaikuttanut siihen jaksamiseen. Että sit semmonen JCI on sitten vaan jäänyt tekemättä ja ei vaan ehditty, ei jaksettu hoitaa sitä eteenpäin. Siis mä olen ihan samaa mieltä kuin (…), että hyvä olis et olis semmonen, jatkuvaa pientä infovirtausta, että pidetään ihmiset ajan tasalla ja näytetään että eteenpäin mennään ja... Mutta se on kuihtunut. En muista milloin meidän alaryhmä olis tavannut viimeksi. Niin kauan siitä.

Haastattelija 1 [00:52:26]: Mitä te ajattelette tästä tän laatujärjestelmän tulevaisuudesta? Mitä tässä tulee tapahtumaan? Tuleeks se koskaan valmiiks vai luovutaanko tästä, vai siirrytäänkö johonkin muuhun järjestelmään niinkun (…) varmaan siirtyy, tai mitä te uskotte että tapahtuu seuraavaksi?

Puhuja 3 [00:52:54]: Kuka haluaa vastata ensin?

Haastattelija 1 [00:53:01]: Onks tähän mahdoton?

Puhuja 1 [00:53:02]: Vaikee vastata tai arvella mitään. Mut kyllähän muutaman kerran kun (…) oon kysellyt, niin on niinkun ehkä jäänyt päällimmäiseksi se ajatus että jos (…) luopuu, niin luovutaanko mekin, että onks tää nyt tän kokoiselle firmalle sitten liian iso kakku ollut alun alkaenkin. Mutta ei me tietenkään siellä nyt tällä hetkellä ajatella, että... Tai mistä tää nyt niinkuin esimerkiksi... Kysyn nyt sinulta haastattelijana, että mistä tämä tämmönen

jännä haastattelu, johon arvottiin osallistujat, mistä tää nyt poiki, että onko tässä nyt semmonen arviointivaihe menossa että mitä tän homman kanssa tehdään.

Haastattelija 1 [00:53:47]: Joo, varmaan kun saadaan nämä tulokset näistä eri haastatteluista ja saadaan tehtyä näistä joku kokonaisuus, niin varmaan sitä myös käytetään siihen että talon johto miettii että mitä täällä tehdään seuraavaks, että kannattaako tätä jättää, mutta ainakin nyt tuntuu, mitä mä oon nyt kuunnellut eri ryhmiä on se, että kaikki on ihan yhtä pihalla, niinkun että tosi... Niinkun oikeesti, että tosi vähän se on näkynyt muille kuin niille jotka on ollut niissä mukana ja ne jotka on ollut mukanakin, niin nekin on ollut tauolla, varmaan koronan tai muidenkin takia, et niinkun... Ja myös tää (…) -yhteys tässä sitten mietityttää kaikkia.

Puhuja 3 [00:54:20]: Joo. No, siis nyt menemättä yksityiskohtiin, toi JCI on aivan valtavan laaja ja todella kunnianhimoista. Sen ongelma on meillä ja suomalaisilla ehkä siinä, että se on enemmän tarkoitettu maihin missä ehkä kehitystaso on kaiken kaikkiaan vielä matalampi. Suomi on varmasti kuitenkin standardien osalta ja vaatimusten osalta jo aika korkealla ja sitten moni asia JCI:ssä tuntuu myös tavallaan turhalta tai puuduttavalta. Kyllä kun tää tuli, mulle oli ihan selvää että tähän kuuluu myös semmosia tekstien luomista missä paasataan tämmöstä yleisviisasta, että mikä on ihanteellinen leikkausosaston ja anestesian kulku ja miten vahditaan että kaikki menee hyvin. Tommosia yleispäteviä lauseita ja sit se printataan ja laitetaan mappiin ja sitten ei kukaan sitä enää lue. Et kyllä tämmösiä varmasti jonkun verran kuuluu tämmöseen, mutta... Mutta käytännön näkökulmasta ja potilasturvallisuuden näkökulmasta, kyllä tässä piileskelee paljon potentiaalia, paljon mahdollisuuksia ja hyötyjä. Et sillä lailla mä melkein myös toivoisin että tätä jatkettais. Eli jonkunlainen tämmönen laatujärjestelmä, et kävisimme koko järjestelmän läpi ja sit saisimmekin vaikka sen myös sen merkinnän ja jota voi sit myös vaikka taas käyttää markkinoinnissa ja... Ja ei olis haitaksi. Mutta se JCI on ehkä... Se on ehkä nyt vaan liian iso ja liian jäykkä. Et mä... Yhtenä esimerkkinä on tää työntekijöiden, niiden ammattipätevyyden tarkistaminen, et... Et varmasti Suomestakin löytyy valelääkäreitä ja valehoitajia, mut se on varmasti ihan tämmönen murto... Niin pieni ongelma Suomessa, että tuntuu turhalta satsata siihen satoja työtunteja, HR:n työtunteja, että se kerää ja tallentaa ja ylläpitää tämmösiä listoja.

Haastattelija 1 [00:58:00]: Meillä taitaa nyt aika loppua.

Puhuja 3 [00:58:01]: Jaa. [?? 00:58:02].

Haastattelija 1 [00:58:03]: On, joo. Tulee seuraava tähän tilaan.

Puhuja 3 [00:58:06]: Joku sanoi, että tähän tulee sitten.

Haastattelija 1 [00:58:07]: Joo, niin meidän täytyy lopettaa. Tää oli todella mielenkiintoista. Teille tosi paljon kiitoksia, kun tulitte. Tässä saatiin paljon uutta tietoa.

Puhuja 3 [00:58:15]: Ole hyvä. (…) puheenvuorosta tuli semmoinen kysymys mieleen, että oletko nyt viime aikoina pitänyt enemmän tämmöisiä haastatteluja.

Haastattelija 1 [00:58:29]: Mä oon pitänyt, joo. Tää on nyt neljäs.

Puhuja 3 [00:58:33]: Neljäs. Syksyn aikana?

Haastattelija 1 [00:58:35]: Kesän ja syksyn aikana.

Puhuja 3 [00:58:38]: Oliko teillä myös jo vaikka pari vuotta sitten vastaavanlaisia?

Haastattelija 1 [00:58:44]: Oli.

Puhuja 3 [00:58:45]: Joo, eli tämä nyt on tavallaan jatkoa siihen.

Haastattelija 1 [00:58:44]: Kyllä.

Puhuja 3 [00:58:45]: Kun (…) kysy että mistä tämä haastattelu nyt kumpuaa, että ehkä tää on myös osittain vastaus (…), että tämmösiä...

Puhuja 1 [00:58:56]: Niin, aivan.

Puhuja 3 [00:58:58]: Tämmösiä oli muutenkin suunnitelmissa, eiks niin.

Haastattelija 1 [00:58:59]: Oli, oli. Kyllä. Tää on ihan alunperin tähän [sovittu? 00:59:01], että pidettäis niinkun sillon alussa kun se otettiin käyttöön, sit vähän niinkun puolessa välissä ja sit vielä myöhemmin parin vuoden... Sit kun se oltais kokonaan otettu käyttöön, niin sitten vielä, jos niinkun päästään siihen tilaisuuteen. Et samalla kysyttäis vähän niinkun henkilökunnaltakin että miten se vaikuttaa teidän työhön ja elämään täällä.

Puhuja 3 [00:59:21]: Joo. Saako... Tietysti et voi kertoa yksityiskohtia kun ne ovat luottamuksellisia, mutta saako kysyä mitkä ammattiryhmät olivat edustettuina näissä aiemmissa.

Haastattelija 1 [00:59:37]: Ihan samalla tavalla kun tässäkin, että kaikista ammattiryhmistä. Että lääkäreitä, fysioterapeutteja, asiakaspalvelijoita... Ihan tosi monenlaisia.

Puhuja 3 [00:59:47]: Ja miksi meitä ei oo kutsuttu kaikkia samaan ryhmään kerrallaan?

Haastattelija 1 [00:59:53]: Ai että kaikki lääkärit ois kerrallaan?

Puhuja 3 [00:59:55]: Tai...

Haastattelija 1 [00:59:56]: Mun mielestä siinä oli joku pohjaidea siitä, että ihmiset myös kuuntelis vähän toisiaan, eri ryhmistä.

Puhuja 3 [00:59:59]: Joo. Ihan... Tää oli vaan...

Haastattelija 1 [01:00:02]: Joo.

Puhuja 3 [01:00:01]: Mielenkiinnon takia kysyin. Joo.

Haastattelija 1 [01:00:03]: Kyllä. Mut kiitos.

[recording ends]
