# Supplementary material for: Implementation and staff perceptions of a quality assurance system in a Finnish private hospital during the COVID-19 Pandemic: A qualitative study
Source: PLoS One. 2025 Dec 4;20(12):e0327271. doi: 10.1371/journal.pone.0327271 (PMC12677441; doi:10.1371/journal.pone.0327271)
Supplement: Attachment — Submitted filename: Comments to the Editor and reviewres.docx [file pone.0327271.s003.docx]

## Remarks for the Editor

*When submitting your revision, we need you to address these additional requirements.*

*1. Please ensure that your manuscript meets PLOS ONE's style requirements, including those for file naming.*

**We have carefully reviewed the provided guidelines and have made every effort to revise the document in accordance with your instructions.**

*2. Thank you for stating the following financial disclosure:
This work was supported by the Orton Research Institute through grants from the Ministry of Social*

*Affairs and Health in Finland, grant no. A2500/495. There was no additional external funding received for this study. Please state what role the funders took in the study. If the funders had no role, please state: "The funders had no role in study design, data collection and analysis, decision to publish, or preparation of the manuscript."
If this statement is not correct you must amend it as needed.
Please include this amended Role of Funder statement in your cover letter; we will change the online submission form on your behalf.*

**Thank you for you comment. We have updated our Funding statement at the row 71 as follows:**

**This work was supported by the Research Institute Orton through grants from the Ministry of Social Affairs and Health in Finland, grant no. A2500/495. There was no additional external funding received for this study.**

**We have added "The funders had no role in study design, data collection and analysis, decision to publish, or preparation of the manuscript." to our Cover letter.**

*3. Thank you for stating the following in your Competing Interests section: No*

*Please complete your Competing Interests on the online submission form to state any Competing Interests. If you have no competing interests, please state "The authors have declared that no competing interests exist.", as detailed online in our guide for authors at http://journals.plos.org/plosone/s/submit-now
This information should be included in your cover letter; we will change the online submission form on your behalf.*

**Thank you for your comment and the information change to the online submission form. We have added:**

**"The authors have declared that no competing interests exist.” to our cover letter**

4. When completing the data availability statement of the submission form, you indicated that you will make your data available on acceptance. We strongly recommend all authors decide on a data sharing plan before acceptance, as the process can be lengthy and hold up publication timelines. Please note that, though access restrictions are acceptable now, your entire data will need to be made freely accessible if your manuscript is accepted for publication. This policy applies to all data except where public deposition would breach compliance with the protocol approved by your research ethics board. If you are unable to adhere to our open data policy, please kindly revise your statement to explain your reasoning and we will seek the editor's input on an exemption. Please be assured that, once you have provided your new statement, the assessment of your exemption will not hold up the peer review process.

**Thank you for your clarification regarding data collection. We have now added the complete anonymized dataset to the Supplementary Materials 3.**

*5. Please include captions for your Supporting Information files at the end of your manuscript, and update any in-text citations to match accordingly. Please see our Supporting Information guidelines for more information:* [*http://journals.plos.org/plosone/s/supporting-information*](http://journals.plos.org/plosone/s/supporting-information)

**Thank you for your comment. We have added a “Supplementary Materials” section after the reference list and included corresponding references to it within the main text.**

*6. Please remove all personal information, ensure that the data shared are in accordance with participant consent, and re-upload a fully anonymized data set.*

*Note: spreadsheet columns with personal information must be removed and not hidden as all hidden columns will appear in the published file.*

*Additional guidance on preparing raw data for publication can be found in our Data Policy (https://journals.plos.org/plosone/s/data-availability#loc-human-research-participant-data-and-other-sensitive-data) and in the following article:* [*http://www.bmj.com/content/340/bmj.c181.long*](http://www.bmj.com/content/340/bmj.c181.long)*.*

**This has been completed. A fully anonymized dataset has been reviewed for compliance with participant consent and added to Supplementary Materials 3.**

*7. If the reviewer comments include a recommendation to cite specific previously published works, please review and evaluate these publications to determine whether they are relevant and should be cited. There is no requirement to cite these works unless the editor has indicated otherwise.*

**Thank you for your comments. We have addressed the reviewer’s feedback in the “Comments to the Author” section.**

**Review Comments to the Author**


1. Abstract:

• The phrase "impact on staff well-being and patient safety" is used, however the abstract doesn't go into detail on staff well-being later on. This produces a gap between the purpose and the findings. So, please make the research goal and the results more similar. You can add results about well-being to the findings.

**Based on your comments, we have extensively revised the manuscript. The specific modifications are detailed in the following sections.**

2. Introduction: The introduction is thorough and well-organised. It talks about the development of accreditation, the gaps in previous research, and the specific goal of the study. Nevertheless, certain amendments are required such as:

• Line 57: "Accreditations have been a part of traditional industries..." Could be reworded to immediately focus on healthcare instead of broad industries.

We sincerely thank you for your valuable feedback. In response, we have revised the sentences on line 57 to the following form:

Accreditations have been part of healthcare for a long time [1]. Accreditation systems have become integral parts [2-4] partly due to globalisation and the growth of medical tourism [5]. International accreditations have emerged alongside national systems to enable the assessment of hospital quality across borders, focusing on standardising protocols to maintain efficiency and safety [6].

• line 82: "This situation brings up an important question: Is accreditation worth pursuing…?” This is a strong rhetorical question; however, it would be better if it were tied directly to the research goal instead of being left hanging.

**Thank you for your kind comment. We have removed the question from line 82. Instead, we have now expanded the Discussion section accordingly.**

• Line 88, The text calls the difference between work satisfaction and well-being "negligible," nevertheless it is important. This could make it harder for readers to understand. Suggestion: This study asserts that well-being and satisfaction are interrelated dimensions of staff experience.

**Thank you for your thoughtful comment. The revision can be seen in the manuscript starting from line 11. We have modified the sentence to the following form:**

Well-being at work ~~can be considered a more comprehensive concept that includes work satisfaction, but the distinction between these two concepts is negligible~~ Well-being at work is a more comprehensive concept that includes job satisfaction, though the distinction is minimal [25, 26]

• The novelty is not distinctly indicated; the introduction resembles a conventional discourse on accreditation rather than providing a compelling rationale for this specific investigation. Through the incorporation of the key characteristics (Finnish private hospital, orthopaedic specialist, ownership transition during COVID-19) are just emphasised at the conclusion and appear to lack significance.

**Thank you very much for this insightful comment. We have thoroughly revised the latter part of the Introduction to highlight the novelty and context of our study more clearly. The modifications can be found starting from line 116.**

~~As mentioned,~~ Patient safety and employee well-being are correlated [27-31] (30) (31, 32, 33, 34).

~~In this study,~~ wWe investigate the effectiveness of hospital accreditation ~~in terms of patient safety and employee well-being through a qualitative study to obtain a comprehensive picture of the hospital during the introduction phase of the quality~~ system regarding patient safety and employee well-being using a qualitative approach, to provide a comprehensive picture of the hospital during the introduction of the quality system. ~~As~~ Øvretveit and Gustafson [32] (35)~~highlighted, the cost of accreditation can be kept at a minimum if the implementation of accreditation is effective. Effectiveness should be investigated more deeply but studies in Norway shows that management and physician involvement at all levels, good data systems, the right training, and effective project team management can be named critical factors of success~~ noted that accreditation costs can be minimised if implementation is efficient. Factors critical for success include management and physician engagement at all levels, robust data systems, appropriate training, and effective project team management [33](36). ~~In turn, the resources allotted to it can be used in other organisational activities. In other words, the money spent on accreditation needs to be evaluated as a long-term investment.~~ ~~Investments in accreditation should thus be considered long-term organisational investments.~~ By focusing on staff perceptions and experiences, this study directly addresses the research questions and ensures that all background information supports understanding the rationale for examining these perceptions

~~This qualitative study examines the perceptions of hospital staff regarding the implementation of a quality assurance system. It aims to describe the staff’s experiences and views on the quality assurance system established in a Finnish private hospital.~~ This qualitative study explores hospital staff perceptions of the implementation of a quality assurance system, aiming to describe their experiences and views on the system established in a Finnish private hospital. It contributes to international literature by examining accreditation in a Nordic private hospital, which is underrepresented in previous research. The focus on an orthopaedic specialist hospital adds relevance, as surgical care and rehabilitation require standardised processes and present specific patient safety challenges. Moreover, the simultaneous ownership transition and the COVID-19 pandemic create a rare opportunity to explore accreditation under conditions of organisational change and crisis. Together, these factors allow the study to shed light on how accreditation influences staff attitudes, well-being, and perceptions of patient safety in a highly dynamic environment—a perspective largely absent from prior studies.

The research questions are as follows:

What are the expectations of the staff regarding the implementation of the quality assurance system?

What advantages and disadvantages do hospital staff perceive in the implementation of the quality assurance system?

~~The research setting is a Finnish private hospital, however owned by a large public hospital. The private hospital is an orthopaedic hospital focused on joint replacements and back surgery and has a rehabilitation centre.~~

3. Materials and Methods: this section is clear and thorough; however, certain modifications are needed such as:
  • Make it clear whether these practitioners work full-time, part-time, or on a temporary basis, as this has a big effect on how people see them.

• Readers can't tell how representative the sample is because there are no percentage distributions for staff groups including doctors, nurses, physiotherapists, and administrative staff.

• The two rounds of data collection conducted in 2020 and 2022 are referenced but lack clear explanation. Were the same individuals involved in the follow-up, or were different groups utilised?

**Thank you for your valuable comment. The Materials and Methods section has undergone substantial revisions. To save space, we have not included the entire modified section here. The updated version of Section 2, Materials and Methods, can be found starting from line 157.**

4. Result: an extensive dataset, authentic staff perspectives, and a distinctive long-term outlook. However, some amendments are needed such as:

• The current section resembles an introduction rather than an analytical discourse. There are themes, for example: initial optimism, practical benefits, staff well-being, rigidity and workload, contextual constraints, ownership shift, and pandemic disruption; however, they lack clear differentiation. It is advisable to split the content into four to six main concepts, accompanied by subheadings. For example:

Initial excitement and optimism (2020)

Perceived benefits include guidelines, orientation, patient safety, and marketing.

Perceived issues include excessive effort, overly strict regulations, and lack of connection to the local context.

Impact of external variables, such as COVID-19, ownership transitions, and staff turnover

Evolution of perspectives over time (reflecting on 2022, progressing towards ISO)

**Thank you for your helpful suggestions regarding the Results section. We have added the following subheadings to improve clarity and structure:**

**Initial excitement and optimism in 2020 — line 285**

**Perceived benefits of the quality system — line 295**

**Perceived challenges and issues — line 344**

**Impact of external variables in 2022 — line 372**

**Evolution of perspectives over time in 2022 — line 442**

In addition to these changes, we have also added content related to patient safety starting from line 421.

‘From a practical perspective and in terms of patient safety, there is indeed a lot of potential here—many opportunities and benefits. In fact, I would almost hope that this would continue. So, some kind of quality system where we could go through the entire system, and then perhaps even receive some kind of certification, which could also be used in marketing, for example… and it certainly wouldn’t hurt.’ (Interview 4, 2022)

5. Discussion: Balanced and contextual, acknowledging both advantages and disadvantages; however, modifications are necessary such as:

• Strengthen the critique by comparing the instrumental and symbolic aims of accreditation, substantiated by citations. as the section says that accreditation may be more useful for marketing than for patient safety, however this might be critically expanded:
Does certification run the risk of becoming more of a symbol than a tool?
How does this stress effect the morale of the staff?

**Thank you for these thoughtful and thought-provoking follow-up questions. We have thoroughly revised the entire Discussion section to address these points in greater depth. To save space, the full updated text can be found starting from line 506.**

*6. Conclusion:

• The comparison between Arab countries and Finland is presented suddenly, without a grounding in the study's data or supporting sources. It could look like guesswork instead than facts that have been proven. It is prudent to undertake this comparison with enhanced clarity for example, "Existing studies demonstrate that in nations with less robust regulatory frameworks..."*

**Thank you for your comment. We have removed the entire paragraph starting from line 679.**

*• The conclusion talks about a number of things, such as resources, health, culture, teamwork, and training. Even though all the parts are important, the message seems a little unclear. Think about focussing on two or three main suggestions: making accreditation fit the situation, making sure managers are committed, and using employee satisfaction as a measure of performance.*

**Thank you for your valuable comments. Starting from line 688, we have added the following content to the Conclusion section:**

Strong managerial commitment is essential for successful implementation. The study showed that even when staff were generally positive about the quality system, achieving accreditation would have required more dedicated leadership, clear goals, and consistent support from management. Managers play a central role in allocating resources, facilitating processes, and fostering a culture that values quality improvement.

Supporting staff well-being and work satisfaction is critical. Ensuring that employees feel valued, engaged, and equipped with the necessary training and resources contributes directly to the effectiveness of any quality assurance system. A positive work environment, collaborative culture, and open communication between leadership and staff not only sustain morale but also enhance the overall performance and sustainability of quality initiatives.

Taken together, these findings suggest that the successful implementation of accreditation depends on balancing contextual fit, managerial commitment, and employee engagement. Future research should examine how these factors interact to influence the long-term outcomes of accreditation in healthcare settings with varying regulatory, financial, and organizational conditions

In Finland, national-level supervision is well established and thorough. In countries where such oversight is absent, accreditation plays a crucial role in standardizing hospital operations. While staff generally had a positive attitude towards quality systems accreditation in the present study, even stronger managerial commitment and significant resources would have been needed to achieve the accreditation. Ensuring quality despite limited resources remains a key challenge, requiring effective strategies and innovative approaches.

***Reviewer #2****:
This is an excellent piece of work addressing an important and relatively unexplored area of study. The topic is timely and relevant, particularly given its focus on the implementation and staff perceptions of a quality assurance system in a Finnish private hospital during the COVID-19 pandemic. Below are my detailed comments and suggestions for improvement.

Background
=As the opening chapter, the background requires important revisions. It should serve as the gateway to the problem under study and must remain focused on the research title. Some paragraphs appear tangential and not directly relevant to the topic; I recommend revising these to maintain focus.*

*= Notably, COVID-19 is not mentioned at all in the background, which is surprising given its centrality to the research. The pandemic context should be explicitly integrated to strengthen the rationale for the study.
= A synthesis of existing literature should be included to demonstrate familiarity with prior work and to clearly establish the research gap.
= The final paragraph of the background (“The research setting is a Finnish private hospital, however owned by a large public hospital...”) would be more appropriately placed under the Methodology section, particularly in the subsection describing the study setting.*

**Thank you for your constructive comments. We have now extensively revised the entire Introduction section. The updated version can be found starting from page 3.**

*Materials and Methods
= The discussion of data collection techniques and analysis approaches is clear and well-articulated.
= However, the methods section would be stronger if it began with the study design and study setting, to provide the reader with a clear orientation before delving into methodological details.*

*= The description of focus group discussions (FGDs) needs clarification:
o How many FGDs were conducted, and what was the exact procedure?
o Who participated? Were all 12 people plus the additional 16 part of the same groups, or different ones? Please clarify how the total of 28 participants was organized.*

*= Provide more detail on participant selection, including criteria, demographic characteristics, and any ethical considerations applied.
= Include a step-by-step description of the data collection process (e.g., how interviews were conducted, whether observations were used, duration, recording, transcription, etc.).
= Expand on the data analysis process: how was the qualitative data processed, coded, and interpreted? Specify the analytical framework or technique applied (e.g., thematic analysis, grounded theory).
= Outline the strategies used to ensure quality and credibility (e.g., triangulation, member checking, reflexivity).
= Provide a clear account of the ethical procedures followed, including consent processes and institutional approval, if applicable.*

**Thank you for your valuable comments. The Materials and Methods section has also undergone extensive revisions. You may review the updated version starting from page 7.**

*Results, Discussion, and Conclusion
= These sections are generally well-written and coherent.*

*= However, I suggest that the strengths and limitations of the study be presented immediately after the discussion. This is a common academic practice and allows the reader to critically assess the scope and validity of the findings.*

**Thank you for your helpful comments. We have moved the Strengths and Limitations section from line 476 to Section 5, now beginning at line 614.**

*Overall Impression
The manuscript is strong and promising, but it requires revisions to improve clarity, focus, and methodological rigor. Addressing the above points will enhance the credibility, scholarly value, and overall impact of the study.*

**We would like to express our sincere gratitude for all the comments and guidance provided throughout the review process. We view these insights as highly valuable contributions that have significantly enriched the quality and depth of our research work.**
